# A multiparty homomorphic encryption approach to confidential federated Kaplan–Meier survival analysis

**DOI:** 10.1038/s41746-026-02790-4

**Published:** 2026-06-08

**Authors:** Narasimha Raghavan Veeraragavan, Svetlana Boudko, Jan Franz Nygård

**Affiliations:** 1 Department of Registry Informatics, Cancer Registry of Norway, Norwegian Institute of Public Health, Oslo, Norway; 2 Norwegian Computing Center, Oslo, Norway; 3 Department of Physics and Technology, The Arctic University of Norway, Tromsø, Norway

**Keywords:** Computational biology and bioinformatics, Health care, Medical research, Mathematics and computing, Biomedical engineering

## Abstract

The proliferation of real-world health data enables multi-institutional survival studies, yet privacy constraints preclude centralizing sensitive records. We present a privacy-preserving federated Kaplan–Meier framework based on threshold CKKS (Cheon-Kim-Kim-Song) homomorphic encryption that supports approximate floating-point computation and encrypted aggregation of per-time-point counts while exposing only public outputs. Sites compute aligned at-risk and event tallies on a shared time grid and encrypt compact vectors; a coordinator aggregates ciphertexts; and a decryptor committee produces partial shares fused per block to recover aggregated plaintexts without releasing per-time-point tables. We prove correctness, stability, and slot-optimal vector packing, and derive scaling laws showing that communication grows linearly with the number of sites and predictably with the number of time points. Empirically, using synthetic breast-cancer data (*N* = 60,000) distributed across 500 sites, encrypted federated curves match the pooled oracle to numerical precision. In contrast, plaintext protocols permit trivial reconstruction by subtraction; our threshold-gated design precludes this attack under the stated threat model, enabling high-fidelity survival estimation with predictable overhead and substantially reduced privacy risk.

## Introduction

Survival analysis is central to clinical and epidemiological research, yet privacy and governance constraints hinder centralizing sensitive records. Federated analytics (FA) alleviates this by keeping data in place while coordinating computation across institutions. We focus on the Kaplan–Meier (KM) estimator and adopt multiparty homomorphic encryption (HE) to protect intermediate statistics throughout the pipeline. A concise comparison of representative leveled fully homomorphic encryption (FHE) schemes appears in Table 1; we adopt CKKS with threshold decryption for its native support of approximate real arithmetic and efficient single-instruction multiple-data (SIMD) packing.

**Table 1:** Comparison of representative FHE schemes used in privacy-preserving analytics

Scheme	Arithmetic	Precision	Bootstr.	Efficiency	Threshold	Use cases
BFV^13,14^	Integer (mod *q*)	Exact	Optional (leveled)	Moderate	Yes	Private queries; encrypted databases
BGV^15^	Integer (mod *q*)	Exact	Optional	Efficient with batching	Yes	Complex integer analytics; encrypted ML
CKKS^6^	Real/complex	Approximate	Optional	High (SIMD packing)	Yes	Floating-point analytics; encrypted ML
FHEW^16^	Boolean gates	Exact	Required	Fast per gate	Limited	Lightweight logic gates
TFHE^17^	Boolean gates	Exact	Required (per gate)	Less efficient overall	Limited	Encrypted control flow; logic circuits
CGGI^18^	Boolean gates	Exact	Required	Fast per gate	Limited	LUTs; conditional logic

“Threshold” indicates widely available threshold decryption in mainstream libraries.

Existing federated KM systems have advanced practice, but four gaps remain that we address.

**A key privacy gap (Gap 1)** lies in subtraction-based reconstruction in plaintext two-round KM, which has rarely been quantified for overlapping, multi-site settings. We formalize and empirically demonstrate its practicality and then eliminate the channel by never releasing per-time-point tables.

**An estimator-level theory gap (Gap 2)** persists beyond cryptographic security proofs and microbenchmarks, with limited analysis of how approximate HE perturbs the *KM estimator*. To address this, we provide (i) correctness equalities for both plain and HE-exact settings, (ii) a CKKS perturbation bound with a uniform-convergence corollary, and (iii) an identifiability result showing that publishing survival alone reveals hazard ratios but not per-time totals or any per-site split.

**A scaling-laws gap (Gap 3)** exists in the form of *communication* and *computational* scaling laws specific to KM. These include the effects of packing choices, the stepwise dependence on the size of the time grid, and the linear dependence on the number of sites and decryptors, none of which are stated in a way that enables cost prediction a priori. We address this by deriving simple laws that align with observed behavior.

**A packing design gap (Gap 4)** remains despite the widespread use of CKKS packing, as we are not aware of a formal *optimality* statement for KM’s two streams (*d*_*t*_, *n*_*t*_) under add-only aggregation. We prove a slot-count lower bound and show that interleaved co-packing is optimal under this model.

Federated Kaplan–Meier (KM) systems to date fall into three main lines: (i) *integer* multiparty/threshold HE (e.g., BFV/BGV) exemplified by FAMHE^1^, which guarantees exact arithmetic but requires fixed-point encodings for real-valued workflows; (ii) *approximate* HE with CKKS^2^, which enables floating-point analytics but, to our knowledge, does not center on estimator-level guarantees for KM nor enforce output gating of per-time-point tables; and (iii) *differential privacy* (DP) approaches^3,4^, which are lightweight yet can degrade curve fidelity in sparse-event regimes. Secure multiparty computation (MPC) solutions ^5^ offer strong cryptographic assurances but typically entail heavier interaction.

Our work is distinct in four respects (Table 2). First, we couple *threshold CKKS* with *output gating* and return only the public survival outputs ${\widehat{S}}_{{\rm{HE}}}(t)$ (and optional bands), never the plaintext (*n*_*t*_, *d*_*t*_) tables, thereby closing the subtraction-based reconstruction channel we quantify. When the combiner is operated by the decryptor committee (off-server), the coordinator observes only ciphertexts throughout; if the combiner is co-located with the coordinator, the coordinator may transiently view aggregated (*n*_*t*_, *d*_*t*_), but the system policy does not release these tables to sites and only publishes ${\widehat{S}}_{{\rm{HE}}}(t)$ (and optional bands).

**Table 2:** Representative federated KM systems across three families

Work	Primitive(s)	Arith.	Reveals (*n*_*t*_, *d*_*t*_)?	Thresh.
Froelicher et al.^1^	BGV/BFV (MHE)	Integer	Often yes (intermediate tables)	Yes
Geva et al.^2^	CKKS (HE)	Floating (approx.)	Varies by release	Optional
Rahimian et al.^3^	DP (+FA)	Real via DP	No (noisy outputs only)	N/A
Veeraragavan et al.^4^	DP (node-level)	Real via DP	No (private curves only)	N/A
**This work**	**CKKS + threshold**	**Floating (approx.)**	**No** (**outputs gated to**${\widehat{S}}_{{\bf{HE}}}({\bf{t}})$)	**Yes**

Integer-HE (BGV/BFV), approximate HE (CKKS), and differential privacy (DP). Our framework differs by (i) threshold CKKS with *output gating* to the public survival curve ${\widehat{S}}_{{\rm{HE}}}(t)$ (no per-time-point tables), (ii) KM-specific estimator-level guarantees, and (iii) closed-form *communication* and *computational* scaling laws.

Second, we provide *estimator-level theory* specific to KM: plain/HE-exact equalities, a CKKS *perturbation bound* with a uniform-convergence corollary, and an *identifiability* result showing that publishing ${\widehat{S}}_{{\rm{HE}}}(t)$ fixes hazard ratios but not counts. Third, we prove a *packing optimality* lemma for interleaving (*n*_*t*_, *d*_*t*_) under add-only aggregation and derive *KM-specific communication and computational laws* that predict costs and explain the observed interleaving speedups.

Fourth, we evaluate up to *K* = 500 sites on a large synthetic cohort (and up to 100 sites on NCCTG lung cancer in section S9 of the Supplementary Information; see Fig. S1 (Supplementary Information)-Fig. S3 (Supplementary Information)), showing numerical indistinguishability from the pooled oracle under both packings and an empirically eliminated reconstruction risk under threshold decryption.

Compared with prior CKKS-based systems, our framework combines threshold CKKS, output gating, estimator-level guarantees for Kaplan–Meier analysis, and explicit communication and computational scaling laws.

Our contributions are:

1. A complete multiparty CKKS framework for federated KM with threshold decryption and output gating that returns only public outputs (the KM curve and optional bands).

2. We ensure estimator-level guarantees: (i) plain federated KM equals the pooled oracle; (ii) exact HE reproduces the oracle; (iii) an explicit CKKS perturbation bound with uniform convergence as noise vanishes; and (iv) an identifiability proposition (hazards determined, counts not).

3. A packing optimality lemma for interleaving (*n*_*t*_, *d*_*t*_) and a *computational scaling law* that separates server additions, committee share generation, fusion, and the final KM pass.

4. We consider closed-form *communication scaling laws* for the two KM rounds and rules of thumb: encrypted-round bandwidth scales linearly with the number of sites and increases in predictable *steps* with the number of time points; decryption-share traffic scales linearly with the decryptor committee size.

5. An empirical study on a large synthetic cohort (up to 500 sites) and an NCCTG lung dataset (see Section S9 of the Supplementary Information; Fig. S1 (Supplementary Information)-Fig. S3 (Supplementary Information)) showing numerical indistinguishability from the pooled estimator under both packings and quantifying the reconstruction risk in plaintext vs. its removal under threshold HE.

We employ a two-round scheme: **R1** discovers the global time grid by unifying local survival times (plaintext); **R2** uploads encrypted per-time-point counts for aggregation and threshold decryption. We consider two packing modes: *interleaved*, which co-packs (*n*_*t*_, *d*_*t*_) pairs to minimize ciphertext count, and *separate*, which packs the two streams independently. Interleaving reduces bandwidth and decryption-share traffic whenever it fits more (*n*_*t*_, *d*_*t*_) pairs into the same slot budget; exact formulas and scaling laws appear in sections “Methods” and “Theoretical Results”. We evaluate deliberately non-IID (independent and identically distributed) client partitions via Dirichlet label splits (*α* = 0.2) and report all main metrics under both regimes, with figures cross-referenced in section “Results”.

Security and efficiency of HE schemes are well studied, but estimator-level KM results under CKKS are, to our knowledge, not formalized in prior federated work. We are not aware of (i) a KM-specific CKKS perturbation bound with a uniform-convergence corollary, (ii) an identifiability statement for publishing ${\widehat{S}}_{{\rm{HE}}}(t)$ without counts in a federated setting, or (iii) a packing optimality proof tailored to KM’s add-only aggregation. Prior systems typically report empirical throughput or asymptotic crypto costs; our scaling laws are end-to-end for the KM workload and align with measured trends. Packing and communication are key practical determinants of add-only Kaplan–Meier deployment because they govern ciphertext count, uplink and share traffic, and runtime at scale. These quantities also inform deployment choices such as packing layout, committee size, and expected bandwidth.

## Results

We evaluate the federated Kaplan–Meier (KM) pipeline along three axes: privacy, numerical fidelity, and system scalability, organized as six research questions (RQ1–RQ6). We adopt the following notation: *K* (number of clients), ∣*T*∣ (size of the event-time grid), *n* (CKKS ring degree; packing *B* = *n*/2 complex slots), ∣*Q*∣ (total modulus bitlength), *R* (committee size), and *θ* (decryption threshold in a *θ*-of-*R* scheme).

Unless stated otherwise, the pooled (centralized) KM curve serves as the *oracle* reference. For each metric, we report the mean and 95% confidence intervals (CIs) across repeated trials. An RQ is considered *met* when all pre-specified criteria in section “ Methods” are satisfied across the swept hyperparameters.

The six questions address privacy (RQ1), numerical and statistical fidelity (RQ2–RQ3), packing (RQ4), runtime (RQ5), and communication (RQ6), followed by analytical results.

*Can a site practically reconstruct other sites’ contributions when per-time-point counts are exchanged in plaintext, and does our CKKS+threshold design remove that attack surface?* (**RQ1**)

We simulate a federated KM setting with *K* ∈ {2, 10, 30, 50, 70, 100} sites under three overlap regimes (*none*, *small*, *large*) and two partitioning styles: IID and non-IID label splits (Dirichlet allocation with *α* = 0.2 over event labels to match section “Partitioning and heterogeneity”). In this reconstruction experiment, overlap refers to duplicate row copies shared across sites; these copies are treated as distinct records in the aggregate multiset. In the plaintext baseline, as commonly implemented in two-round KM, the coordinator broadcasts aggregated per-time-point counts $({n}_{t}^{({\rm{Fed}})},{d}_{t}^{({\rm{Fed}})})$ to all sites, or sites can independently inspect them at the coordinator. An honest-but-curious site *A* then computes the residual

$${n}_{t}^{({\rm{others}})}={n}_{t}^{({\rm{Fed}})}-{n}_{t}^{(A)},\,{d}_{t}^{({\rm{others}})}={d}_{t}^{({\rm{Fed}})}-{d}_{t}^{(A)},$$

thereby attempting to recover the combined contributions of the remaining sites.

**Fig. 1:**
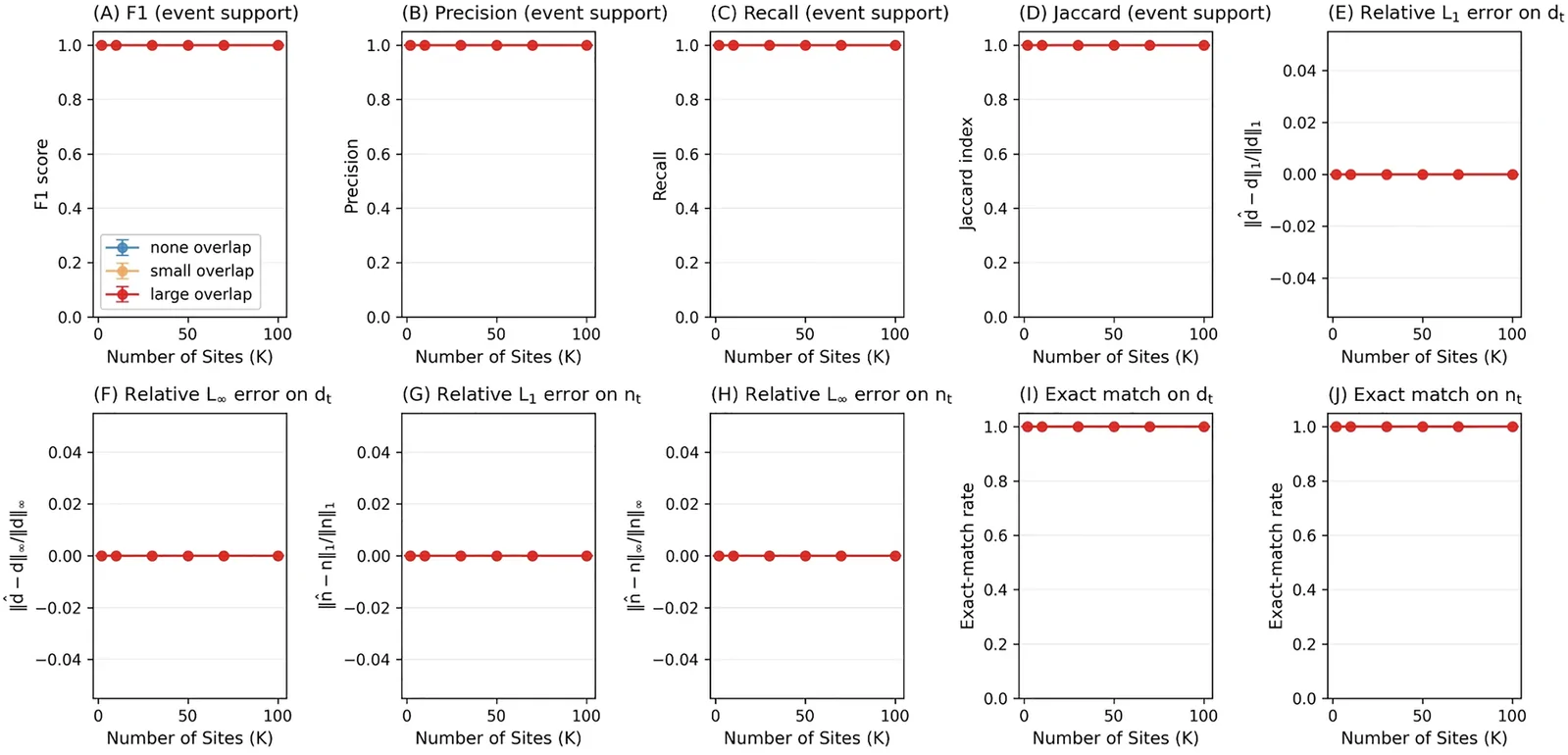
Reconstruction metrics vs. number of sites *K* for IID data partitioning on the Synthetic Breast dataset. Ten metrics are shown: **A** F1 score for event support, **B** precision, **C** recall, **D** Jaccard index, **E** relative *L*_1_ error on *d*_*t*_, **F** relative *L*_*∞*_ error on *d*_*t*_, **G** relative *L*_1_ error on *n*_*t*_, **H** relative *L*_*∞*_ error on *n*_*t*_, **I** exact-match rate on *d*_*t*_, and **J** exact-match rate on *n*_*t*_. Curves show means with 95% confidence intervals. Three overlap conditions are compared: none (blue), small (orange), and large (red). All conditions yielded identical results, demonstrating exact reconstruction regardless of overlap.

**Fig. 2:**
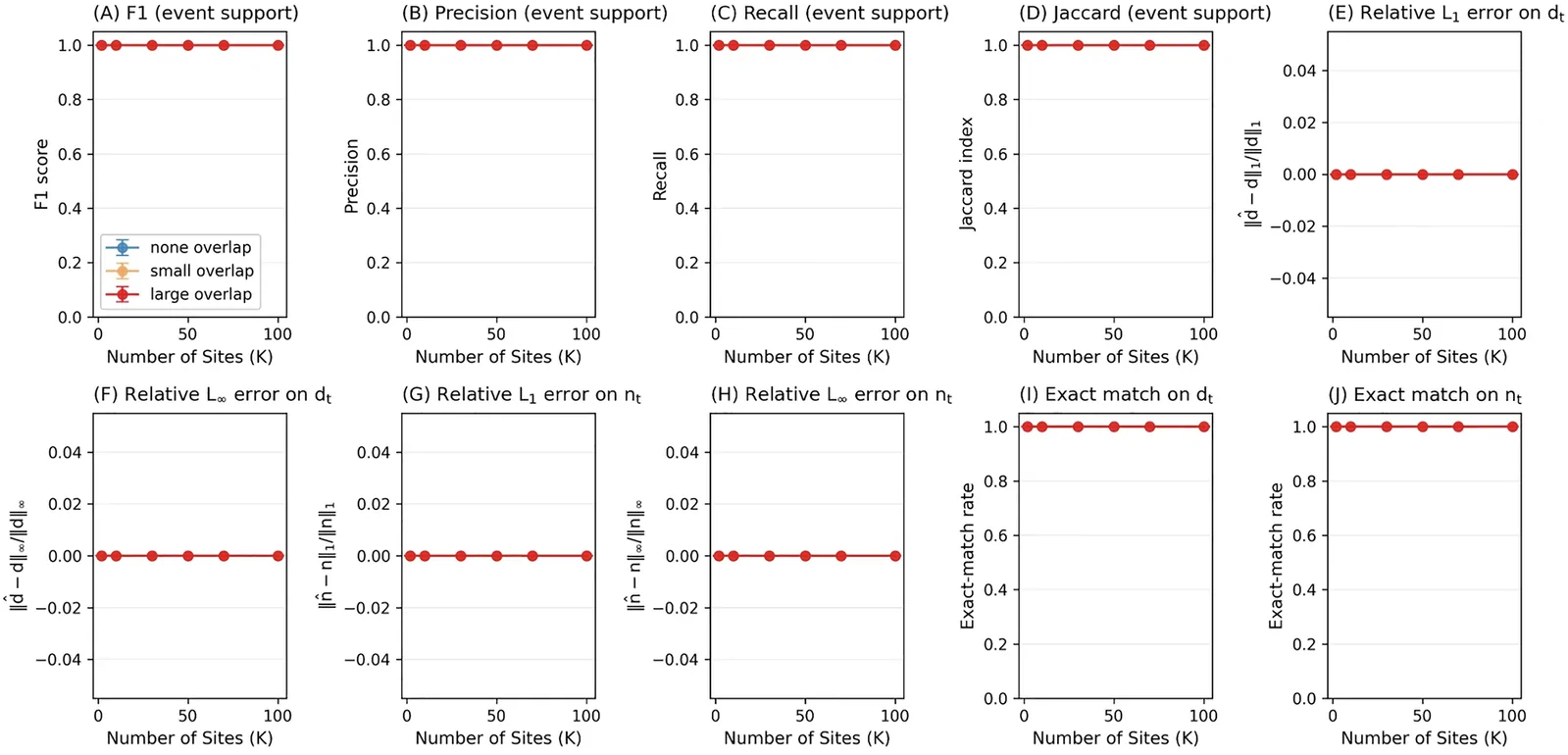
Reconstruction metrics vs. number of sites *K* for label non-IID data partitioning (Dirichlet *α* = 0.2) on the Synthetic Breast dataset. Ten metrics are shown: **A** F1 score for event support; **B** precision for event support; **C** recall for event support; **D** Jaccard index for event support; **E** relative L_1_ error on d_t_; **F** relative L_∞_ error on d_t_; **G** relative L_1_ error on n_t_; **H** relative L_∞_ error on n_t_; **I** exact-match rate on d_t_; and **J** exact-match rate on n_t_. Three overlap conditions are compared: none, small, and large. All overlap conditions yielded identical results, demonstrating exact reconstruction of the combined residual contribution of the other sites even under heterogeneous label non-IID data distributions.

**Fig. 3:**
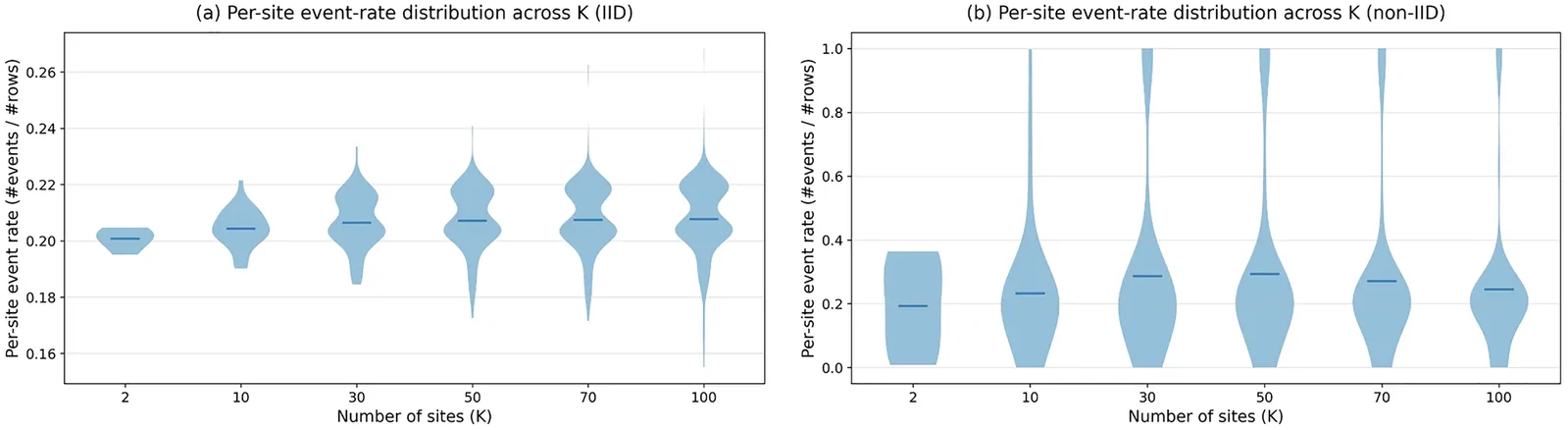
Per-site event-rate distributions for the synthetic breast dataset. Violin plots show the distribution of event rates (#events/#rows) across sites under **a** IID partitioning and **b** label non-IID partitioning. Wider, skewed violins under non-IID indicate heterogeneous site contributions but do not prevent exact residualization when plaintext per-time-point counts are disclosed.

We evaluate both per-time-point event counts *d*_*t*_ and numbers at risk *n*_*t*_ across *K* and overlap regimes, reporting two consolidated 2 × 5 grids (mean with 95% CIs): one for IID (Fig. 1) and one for non-IID label splits (Fig. 2). Panels (A–D) summarize support identification for event times (F1, precision, recall, and Jaccard on ${\mathcal{S}}=\{t:{d}_{t} > 0\}$; higher is *worse* for privacy). Panels (E–H) report relative errors (lower is better):

$$\frac{\parallel \widehat{{\bf{d}}}-{\bf{d}}{\parallel }_{1}}{\parallel {\bf{d}}{\parallel }_{1}+{\varepsilon }_{0}},\,\frac{\parallel \widehat{{\bf{d}}}-{\bf{d}}{\parallel }_{\infty }}{\parallel {\bf{d}}{\parallel }_{\infty }+{\varepsilon }_{0}},\,\frac{\parallel \widehat{{\bf{n}}}-{\bf{n}}{\parallel }_{1}}{\parallel {\bf{n}}{\parallel }_{1}+{\varepsilon }_{0}},\,\frac{\parallel \widehat{{\bf{n}}}-{\bf{n}}{\parallel }_{\infty }}{\parallel {\bf{n}}{\parallel }_{\infty }+{\varepsilon }_{0}},$$

with *ε*_0_ = 10^−12^ to avoid division by zero and norms restricted to indices where denominators are positive. Panels (I–J) show exact-match rates

$$\frac{1}{| T| }\mathop{\sum }\limits_{t}{\bf{1}}\{{\widehat{d}}_{t}={d}_{t}\},\,\frac{1}{| T| }\mathop{\sum }\limits_{t}{\bf{1}}\{{\widehat{n}}_{t}={n}_{t}\},$$

where higher values are worse for privacy. To contextualize heterogeneity, we also plot per-client event-rate distributions under IID vs. non-IID (two-panel figure, Fig. 3).

Across all *K* and overlap regimes, both IID and non-IID grids exhibit saturated support metrics (A)–(D) at 1.0, vanishing relative errors (E)–(H) near 0, and exact-match rates (I)–(J) at 1.0. In other words, once $({n}_{t}^{({\rm{Fed}})},{d}_{t}^{({\rm{Fed}})})$ are disclosed in plaintext, subtracting a site’s own $({n}_{t}^{(A)},{d}_{t}^{(A)})$ exactly recovers the combined “others” $({n}_{t}^{({\rm{others}})},{d}_{t}^{({\rm{others}})})$ on the common time grid, independent of *K*, overlap, or partition style. The flat lines with tight, often invisible, error bars reflect that this is an algebraic identity given exact counts and synchronized times.

The plaintext protocol reveals precisely the information the attacker needs. If the federated aggregator broadcasts $({n}_{t}^{({\rm{Fed}})},{d}_{t}^{({\rm{Fed}})})$, as is typical in unencrypted two-round KM, any site can compute the residual and thereby learn the groupwise contributions of the remaining sites; with *K* = 2 this equals the other site exactly. This conclusion does *not* rely on IID assumptions. Non-IID label skew or overlap only change how counts are distributed across *t*, not the identity *x*^(others)^ = *x*^(Fed)^ − *x*^(*A*)^ for *x* ∈ {*n*, *d*}.

The event-rate figure (Fig. 3) shows that IID splits produce tightly clustered per-client event shares, whereas non-IID splits produce broader, skewed distributions. These differences matter for *inference from the survival curve alone*, but they do not mitigate the plaintext subtraction attack, which operates directly on disclosed counts. Overlap increases redundancy in shared event times, but again does not impede exact residualization once totals are visible.

Our CKKS-based design removes the subtraction channel by withholding intermediate plaintext counts from sites and publishing only ${\widehat{S}}_{{\rm{HE}}}(t)$, plus optional derived outputs such as confidence bands. Sites send encrypted packed streams; decryption requires a threshold of parties; and when the combiner runs off-server within the decryptor committee, the coordinator observes only ciphertexts. If the combiner is co-located with the coordinator, the coordinator may transiently view aggregated (*n*_*t*_, *d*_*t*_) to evaluate ${\widehat{S}}_{{\rm{HE}}}(t)$, but per-time-point tables are not disclosed to sites. Because the attacker lacks $({n}_{t}^{({\rm{Fed}})},{d}_{t}^{({\rm{Fed}})})$ in plaintext, the subtraction step is unavailable, and reconstructing others’ counts from ${\widehat{S}}_{{\rm{HE}}}(t)$ alone is under-determined without additional side information.

When per-time-point counts are exchanged in plaintext, reconstruction of other sites’ contributions is trivially exact in our experiments, with perfect support and exact matches across all *K* and partition styles. Encrypting those counts with CKKS and revealing only the final survival outputs removes the subtraction channel and thus the demonstrated privacy risk.

*Do KM curves computed via our CKKS-based federated pipeline agree with the pooled (oracle) estimator as K grows?* (**RQ2**)

For each *K* ∈ {2, 10, 30, 50, 70, 90, 100, 200, 300, 500}, we run the full protocol twice: once with *interleaved* packing and once with *separate* packing. The oracle curve is computed on the pooled plaintext data under identical time grids. We report means with 95% CIs across 10 repeated runs; interleaved and separate lines are overlaid in each panel.

We pre-specified equivalence metrics to assess numerical agreement between the HE pipeline and the pooled oracle across survival, RMST, band, and hazard spaces (Table 8). For the empirical hazard-space comparisons, we use the negative-log survival transform, defined as the negative natural logarithm of the estimated survival curve. Table 8 lists each metric, its formula, and the pass threshold used to declare “no practical difference.” Continuous-time definitions are used unless noted; thresholds are chosen to sit well above floating-point epsilon while remaining below CKKS rounding noise for our parameterization.

**Fig. 4:**
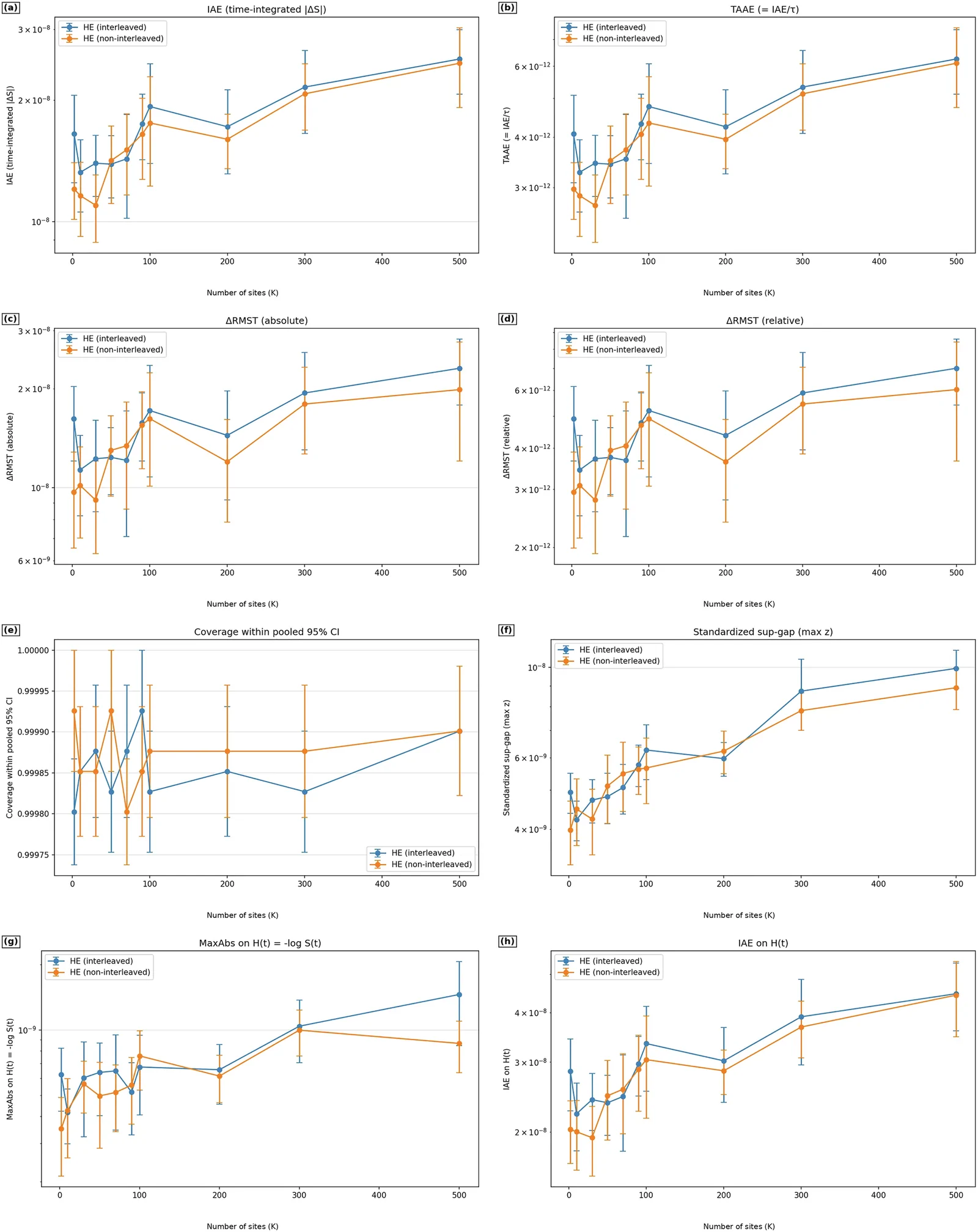
Numerical fidelity metrics for HE-federated Kaplan–Meier vs. pooled oracle across number of sites *K* on the Synthetic Breast dataset. Eight metrics are shown: **a** integrated absolute error on survival function IAE(*S*), **b** time-averaged absolute error TAAE(*S*), **c** absolute difference in restricted mean survival time ∣Δ RMST∣, **d** relative RMST difference Δ RMST_rel_, **e** coverage probability of 95% confidence intervals, **f** standardized supremum gap supZ measuring maximum standardized deviation, **g** maximum absolute difference in the negative log survival transform $\mathop{\max }\limits_{t}| {H}_{{\rm{HE}}}-{H}_{{\rm{oracle}}}|$, and **h** integrated absolute error in the negative-log survival transform IAE_*H*_. Each panel compares interleaved (blue) and non-interleaved/separate (orange) packing strategies with means and 95% confidence intervals. The two packing strategies are numerically indistinguishable across all metrics as *K* increases.

**Fig. 5:**
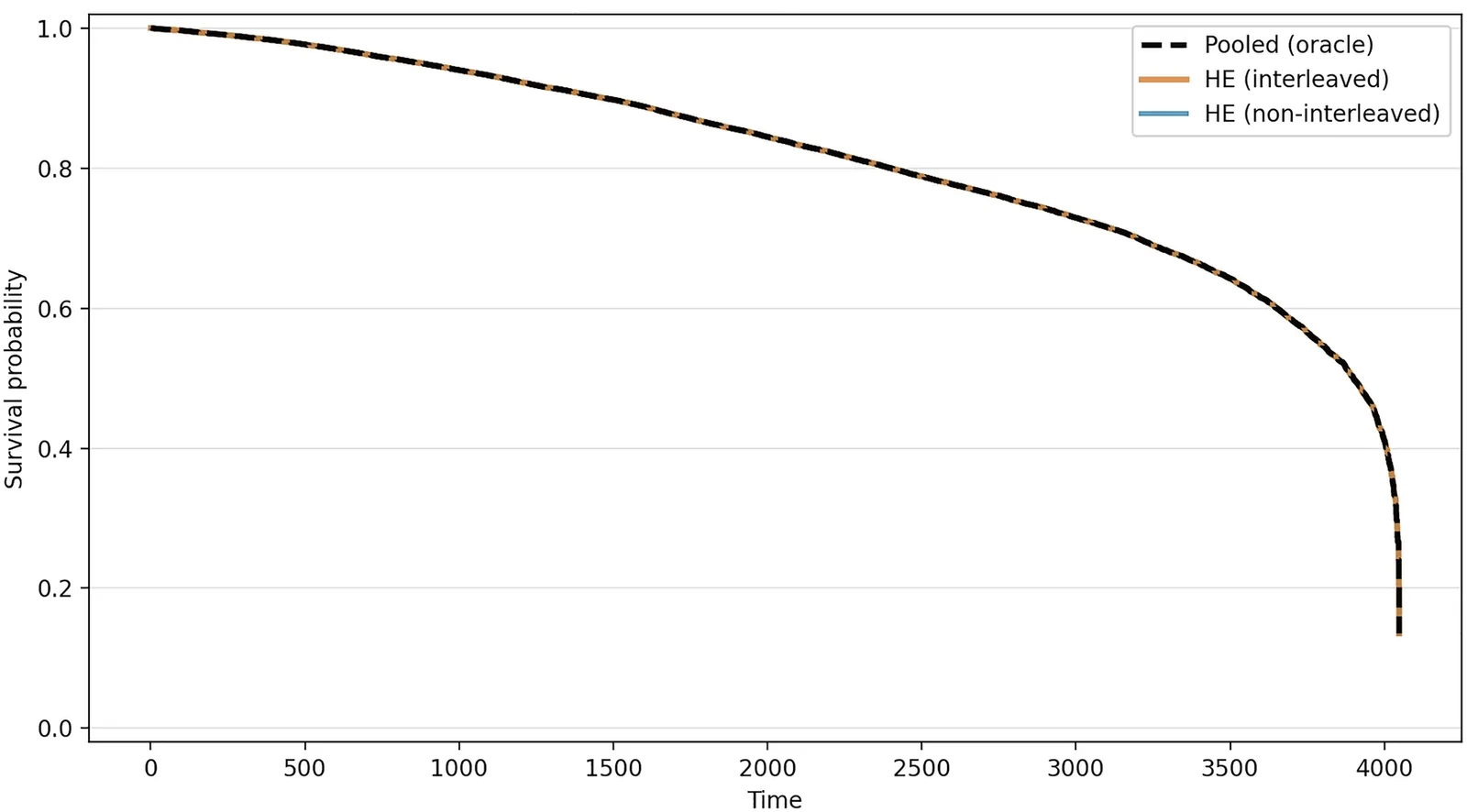
Kaplan–Meier survival curves for *K* = 500 sites demonstrating visual indistinguishability between the pooled oracle (dashed black line), HE-federated with interleaved packing (orange), and HE-federated with non-interleaved/separate packing (blue). All three curves overlap almost perfectly, confirming that homomorphic encryption preserves statistical accuracy even at large scale.

Figure 4 summarizes eight diagnostics in a 2 × 4 grid. Across all *K*, both HE configurations track the pooled oracle to within the numerical noise floor. Visually (Fig. 5), survival curves and bands are indistinguishable; numerically, all equivalence tests, including TAAE and IAE on *S*, absolute and relative RMST gaps, sup-*Z* and coverage, and hazard-based metrics, pass for every *K* and both packings. Packing choice affects efficiency, not accuracy: interleaving reduces communication and runtime, while fidelity remains unchanged.

- *Survival*. IAE(*S*) remains at the 10^−8^ scale (typically 1.3 × 10^−8^ to 2.6 × 10^−8^); TAAE(*S*) is at 10^−12^ scale (typically 3 × 10^−12^ to 6 × 10^−12^). Interleaved and separate curves overlap within noise for every *K*.
- *RMST*. Absolute gaps are ${\mathcal{O}}(1{0}^{-8})$ and relative gaps ${\mathcal{O}}(1{0}^{-12})$ to ${\mathcal{O}}(1{0}^{-8})$, indicating machine-precision agreement with no practical *K*-dependence.
- *Bands*. Coverage is essentially 1.0 (0.99975 to 0.99993) and supZ is 10^−10^ to 10^−9^ across *K*; all runs satisfy the pre-specified supZ criterion.
- *Hazard*.$\mathop{\max }\limits_{t}| {H}_{{\rm{HE}}}-{H}_{{\rm{oracle}}}|$ is 10^−10^ to 10^−9^ and IAE_*H*_ is 2 × 10^−8^ to 4.5 × 10^−8^, with negligible sensitivity to *K* or packing.

Across all *K* and both packings, the HE-federated KM curves *match* the pooled oracle to numerical precision in survival, RMST, band coverage, supremum gap, and hazard, indicating that encryption and packing have no observable effect on statistical fidelity.

*Are the HE curves statistically equivalent to the pooled estimator within pre-specified tolerances?* (**RQ3**)

We assess statistical equivalence using the same setup and figure as in Numerical Fidelity, namely the coverage and standardized sup-gap panels in Fig. 4. Specifically, we report: (i) empirical 95% coverage of the pooled Greenwood bands evaluated at ${\hat{S}}_{\mathrm{HE}}(t)$, and (ii) the standardized sup-gap

$$\mathop{\sup }\limits_{t}\frac{\left|{\hat{S}}_{{\rm{HE}}}(t)-{\hat{S}}_{{\rm{oracle}}}(t)\right|}{\widehat{{\rm{SE}}}\,\left[{\hat{S}}_{{\rm{oracle}}}(t)\right]}.$$

We also evaluate two pre-specified equivalence tests: total time-averaged absolute error within its threshold (Table 8) and relative RMST gap within its threshold (Table 8).

Across all *K* and for both CKKS packings (interleaved and separate), the pooled 95% Greenwood bands *contain*${\widehat{S}}_{{\rm{HE}}}$ essentially everywhere (mean containment > 0.999), and the standardized sup-gap is numerically ≈ 0 (median < 10^−8^). Both equivalence checks pass for 100% of replicates at every *K*. Interleaving does not change inference: HE-interleaved and HE-separate overlay within Monte Carlo noise.

Within our pre-specified tolerances, ${\widehat{S}}_{{\rm{HE}}}(t)$ is statistically equivalent to the pooled oracle ${\widehat{S}}_{{\rm{oracle}}}(t)$ for all *K* and packing choices; the HE pipeline preserves the statistical behavior of the KM estimator.

*Do interleaved vs. non-interleaved CKKS packings change accuracy or only efficiency/communication?* (**RQ4**)

We compare two encodings of the per-time-point statistics (*d*_*t*_, *n*_*t*_) under identical CKKS parameters and the same federated add–decrypt workflow: (i) *interleaved*, where one ciphertext stream co-packs [*n*_1_, *d*_1_, *n*_2_, *d*_2_,…], and (ii) *separate*, where distinct ciphertext streams pack [*d*_1_,…] and [*n*_1_,…]. Any accuracy differences therefore arise solely from packing layout. Figure 4 compares both packings under identical CKKS parameters.

Across all *K* tested (2–500), interleaved and separate packings are *indistinguishable in accuracy* within numerical noise: the two curves overlap in every panel of Fig. 4, their point estimates track across *K*, and their 95% CIs are nearly identical. IAE and TAAE remain at machine-precision levels in both modes; absolute and relative RMST differences are negligible; empirical coverage stays essentially 1.0; and the standardized sup-gap is ≈0. All pre-specified equivalence checks pass for both packings at every *K*.

Packing serves as an efficiency knob rather than an accuracy knob, since interleaving preserves numerical and statistical fidelity to the pooled oracle just as well as separate packing (Fig. 4). Choose interleaving to gain efficiency, not to trade off accuracy.

*How does end-to-end runtime scale with the number of sites K for plain vs. HE modes?* (**RQ5**)

*Setup:* All implementation measurements in this subsection use the deployed alignment grid *T*_all_, so the packed length is *L* = ∣*T*_all_∣. We measure the end-to-end wall-clock runtime of the experimental benchmark pipeline, including cryptographic setup, federated aggregation, threshold decryption, Kaplan-Meier evaluation, and associated implementation overhead. We compare: (i) a *Plain* pooled oracle (no encryption), (ii) HE with *interleaved* CKKS packing (co-packed slots), and (iii) HE with *separate* packing (distinct streams). All CKKS parameters, data, and code paths are identical across conditions except for packing. We sweep *K* ∈ {2, 10, 30, 50, 70, 90, 100, 200, 300, 500} and report means with 95% CIs.

**Fig. 6:**
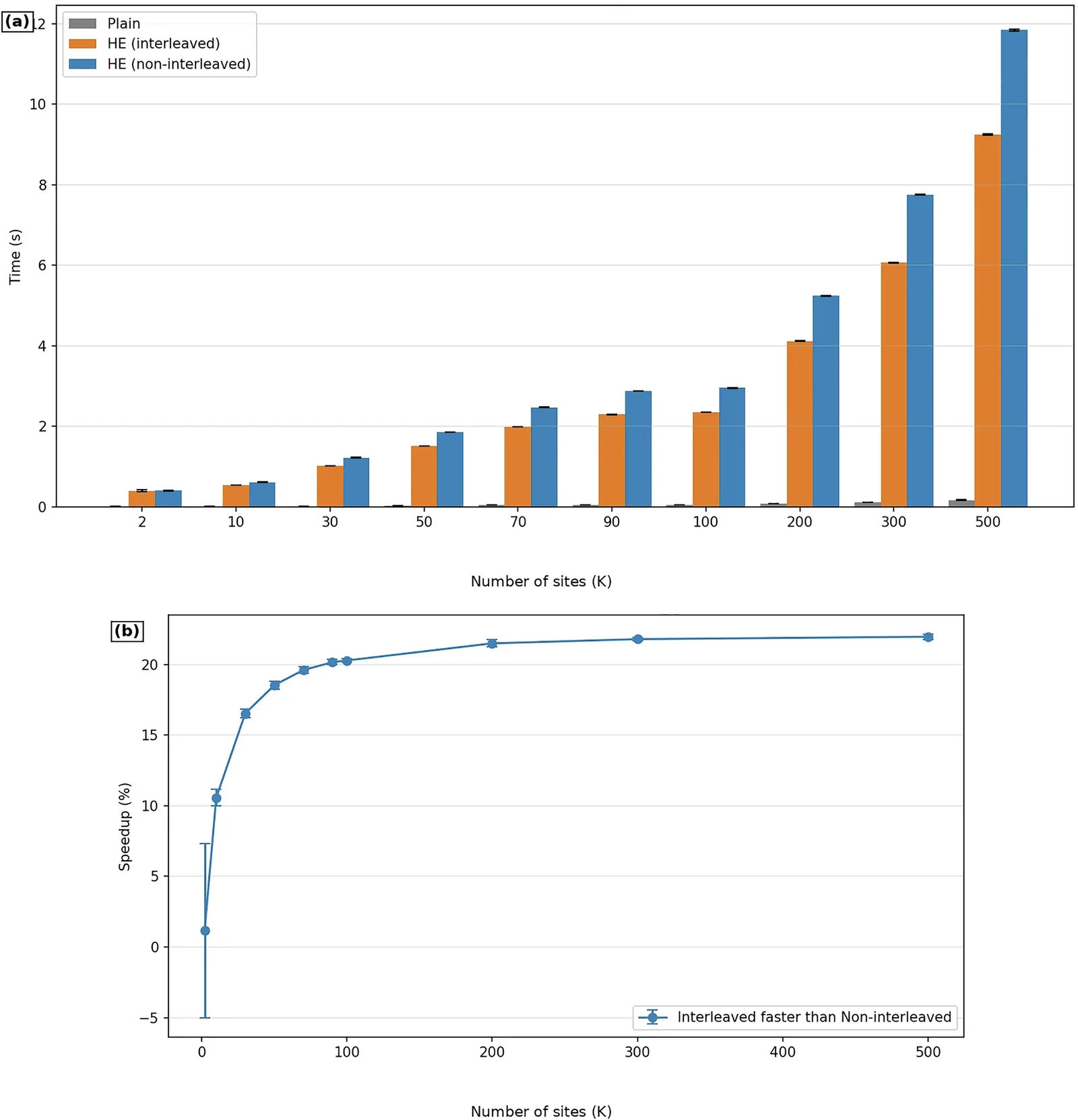
Computational scalability of HE-federated Kaplan–Meier on the Synthetic Breast dataset. **a** End-to-end runtime vs. number of sites *K*, showing approximately linear growth. Plain pooled oracle (gray) serves as a baseline reference, while HE with interleaved packing (orange) and non-interleaved/separate packing (blue) are shown with means and 95% confidence intervals. **b** Percentage speedup of interleaved over non-interleaved packing demonstrates increasing computational gains as *K* rises, plateauing at approximately 22% improvement for large-scale federations.

End-to-end wall-clock runtime (seconds) is used as the evaluation metric (Fig. 6a). We also report the percentage speedup of interleaved over separate packing (Fig. 6b),

$${\rm{speedup}}\,[ \% ]\,=\,100\cdot \frac{{W}_{{\rm{sep}}}-{W}_{{\rm{int}}}}{{W}_{{\rm{sep}}}}\,,$$

where *W*_sep_ and *W*_int_ denote the mean wall-clock runtimes under separate and interleaved packing, respectively.

Figure 6 places runtime trends side-by-side. Panel (a) plots mean runtime vs. *K* for *Plain*, *HE-Interleaved*, and *HE-Separate* with 95% CIs; Panel (b) plots the percentage speedup of interleaved over separate vs. *K*. Curves are monotone and roughly linear for the two HE modes, with a widening gap in favor of interleaving as *K* grows.

- *Near-linear scaling inKfor HE*. HE runtimes grow approximately linearly with the number of sites, consistent with an *O*(*K*) aggregator-addition cost per ciphertext. For interleaved packing, mean time rises from ≈0.39 s at *K* = 2 to ≈9.24 s at *K* = 500; separate packing rises from ≈0.40 s to ≈11.84 s over the same range. CIs are tight across all *K*.
- *Interleaving accelerates compute without affecting accuracy*. Interleaved packing is consistently faster, with the speedup *increasing* with *K*: about 1–2% at *K* = 2, approximately 10.6% at *K* = 10, approximately 16.5% at *K* = 30, and approximately 22% by *K* = 300–500. This matches the reduction in ciphertext counts and additions per round when (*n*_*t*_, *d*_*t*_) are co-packed (see Corollary 14).
- *Pooled-oracle baseline*. The pooled-oracle baseline remains in the millisecond regime (for example, approximately 5–160 ms from *K* = 2 to *K* = 500) since it omits encryption, transmission, and decryption. It serves as a lower bound rather than a deployable privacy-preserving alternative.

HE-federated KM is computationally predictable: end-to-end runtime scales roughly linearly with the number of sites, and interleaved packing yields a consistent 10–22% speedup at moderate-to-large *K* while maintaining identical accuracy (see Proposition 18).

*How do uplink/downlink costs scale withK*, *the number of event-time points* ∣*T*∣, *and CKKS packing; and how large is the interleaving benefit?* (**RQ6**)

This subsection reports formula-based communication simulations rather than measurements from the current implementation. Unless stated otherwise, these simulations instantiate the generic formulas with *L* = ∣*T*∣ to model an event-time-only packing variant. By contrast, the deployed pipeline packs over *T*_all_, i.e., *L* = ∣*T*_all_∣.

We evaluate communication using formula-based simulations implemented in our experimental evaluation. Each round, every site encrypts and uploads aligned per-time-point vectors over a packed grid of length *L* (in the communication simulations presented here, *L* = ∣*T*∣); the aggregator sums and triggers threshold decryption with *R* decryptors. We compare *separate* packing (two ciphertext streams for *d*_•_ and *n*_•_) versus *interleaved* packing (co-packing (*n*_*t*_, *d*_*t*_) across slots). All runs use the same CKKS parameters (*n* = 16,384, ∣*Q*∣ ≈ 438 bits), giving *B* = 8192 complex slots and ciphertext size ct_bytes ≈ 1,794,048 bytes and partial-share size ${\mathtt{share}}\_{\mathtt{bytes}}\approx 897,024$ bytes.

**Fig. 7:**
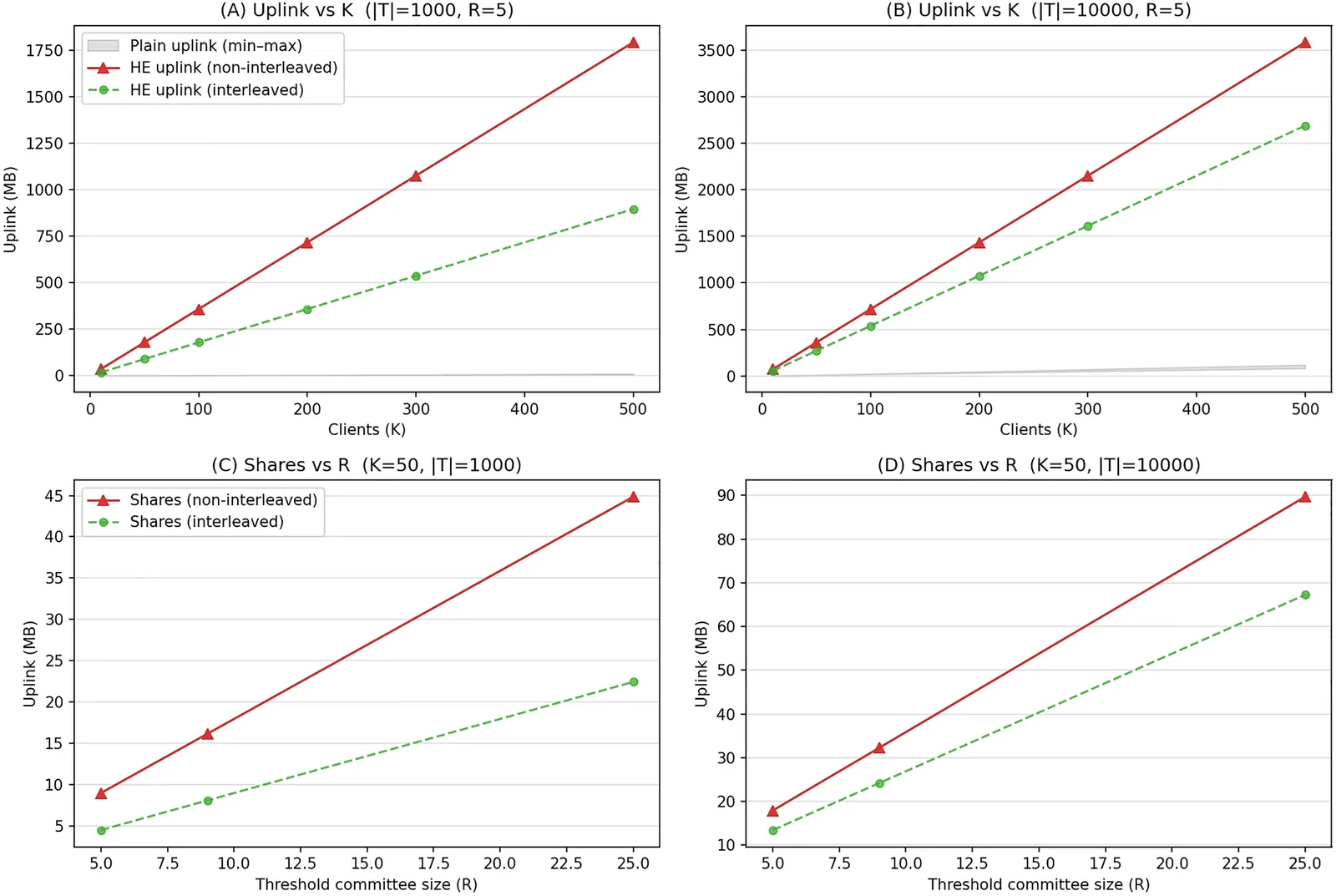
Communication scaling. **A** Uplink vs. *K* with ∣*T*∣ = 1000 and *R* = 5; **B** Uplink vs. *K* with ∣*T*∣ = 10,000 and *R* = 5; **C** decryption-share uplink vs. *R* with *K* = 50 and ∣*T*∣ = 1000; **D** decryption-share uplink vs. *R* with *K* = 50 and ∣*T*∣ = 10,000.

**Fig. 8:**
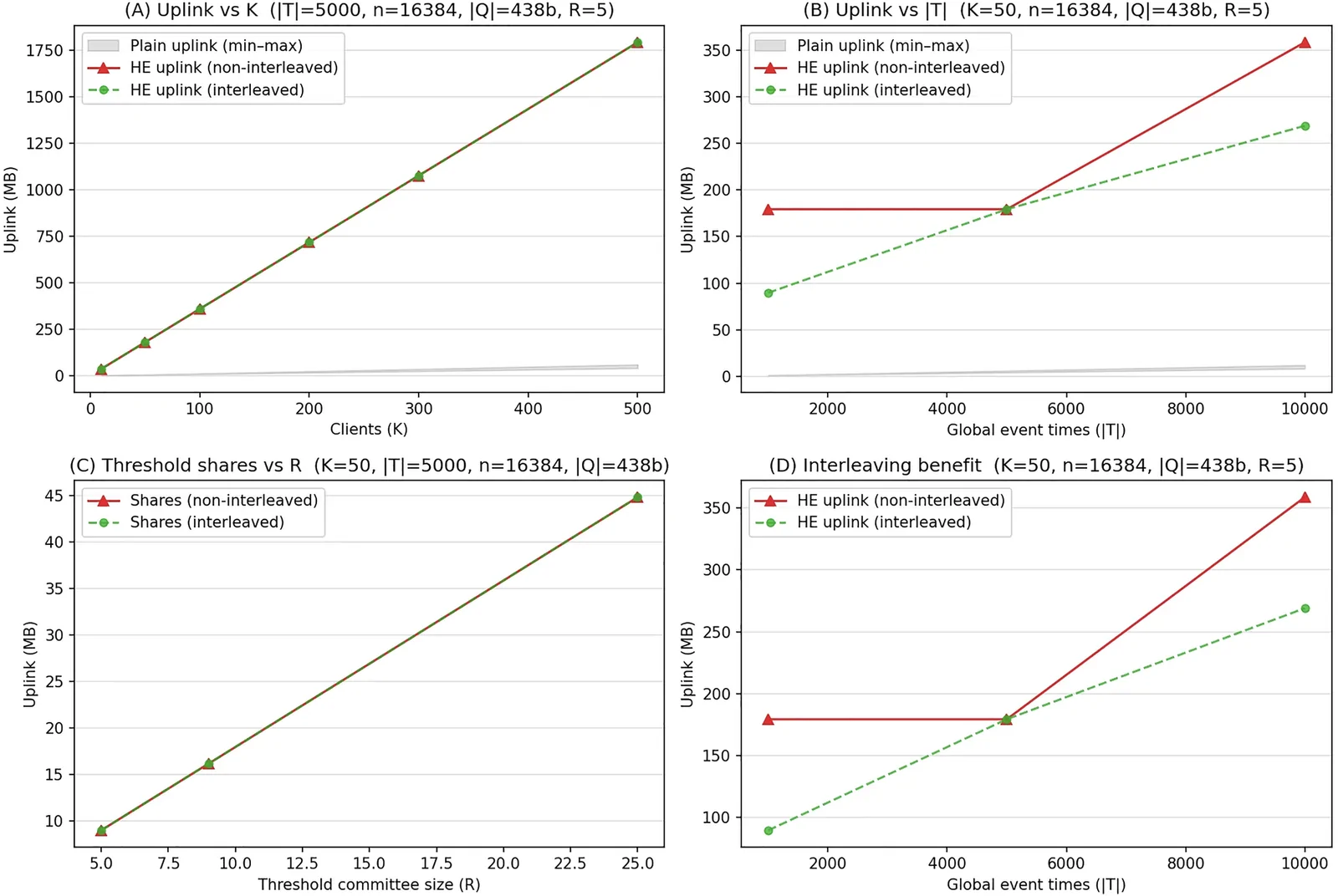
Communication scaling under CKKS packing. **A** HE uplink vs. *K* at fixed ∣*T*∣ = 5000 and *R* = 5; **B** HE uplink vs. ∣*T*∣ at fixed *K* = 50 and *R* = 5; **C** decryption-share uplink vs. *R* at fixed *K* = 50 and ∣*T*∣ = 5000; **D** interleaving benefit shown as HE uplink for non-interleaved and interleaved packing vs. ∣*T*∣, at fixed *K* = 50 and *R* = 5.

**Fig. 9:**
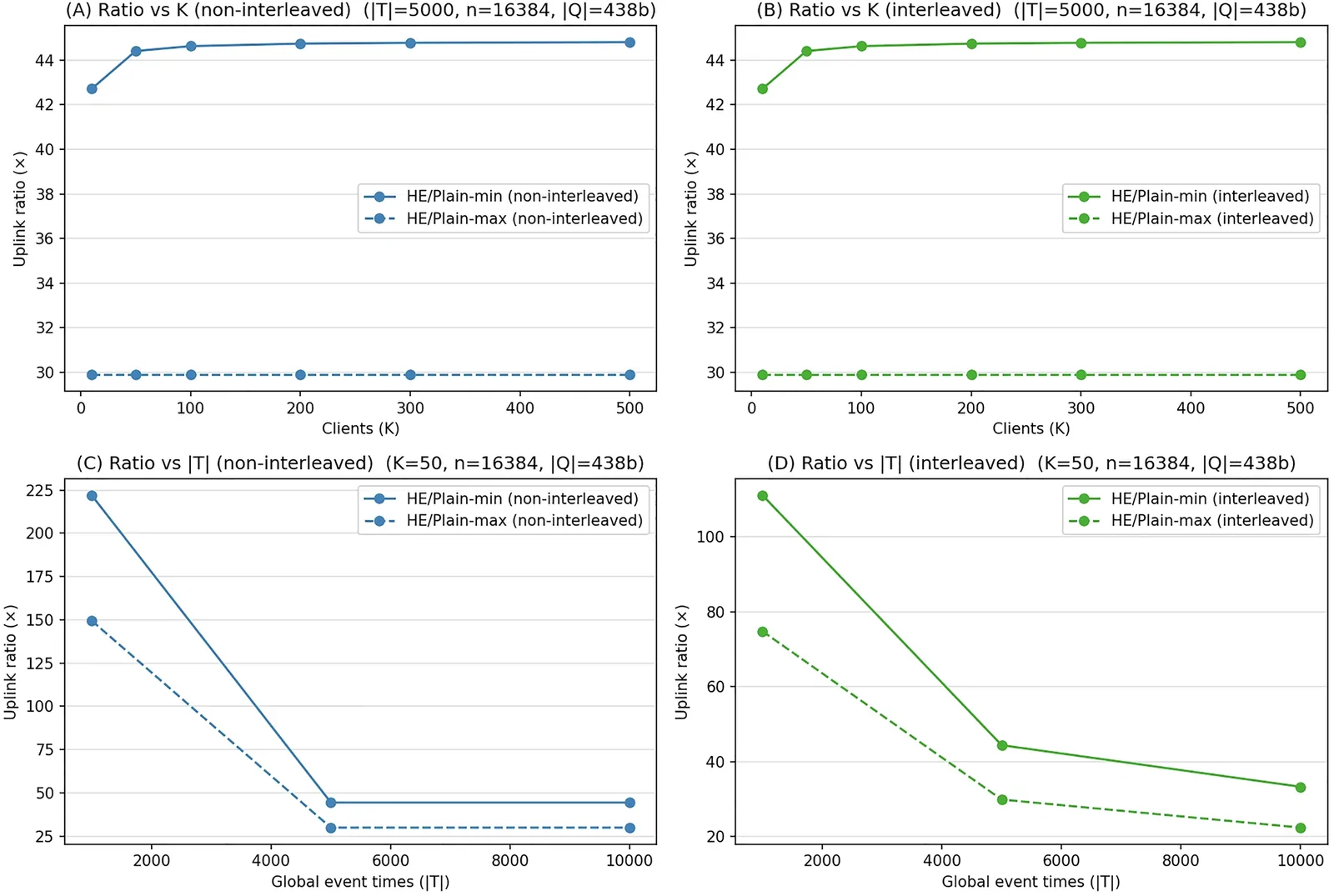
HE-versus-plaintext uplink ratio. **A** Non-interleaved HE/plain uplink ratio vs. *K* at fixed ∣*T*∣ = 5000; **B** interleaved HE/plain uplink ratio vs. *K* at fixed ∣*T*∣ = 5000; **C** non-interleaved HE/plain uplink ratio vs. ∣*T*∣ at fixed *K* = 50; **D** interleaved HE/plain uplink ratio vs. ∣*T*∣ at fixed *K* = 50. Solid lines use the plaintext minimum-uplink denominator and dashed lines use the plaintext maximum-uplink denominator.

All communication quantities are parameterized by the number of event-time points ∣*T*∣, consistent with the simulation code used to generate Figs. 7, 8, 9.

Let *L*: = ∣*T*∣ denote the packed vector length in the communication simulations. The number of ciphertexts per client is

$${M}_{{\rm{int}}}=\left\lceil \frac{2L}{B}\right\rceil ,\,{M}_{{\rm{sep}}}=2\left\lceil \frac{L}{B}\right\rceil .$$

Per-client uplink is *M* × ct_bytes, so total uplink scales linearly in *K*. Decryption-share traffic scales as

$$R\times M\times {\mathtt{share}}\_{\mathtt{bytes}}.$$

The *interleaving benefit* is defined as

$${\rm{benefit}}=\frac{{M}_{{\rm{sep}}}}{{M}_{{\rm{int}}}},$$

which equals 2 × when *L* ≤ *B*/2, 1 × when both packings use the same number of ciphertexts, and intermediate values such as 4/3 × when (*M*_sep_, *M*_int_) = (4, 3).

We also report the *HE/Plain uplink ratio*. The plaintext protocol uploads aligned counts over *T* using 2*K*∣*T*∣ scalars, whereas the HE protocol uploads ciphertexts. This ratio is therefore independent of *K* but depends on ∣*T*∣ through the stepwise behavior of *M*.

Figures 7–9 show communication scaling as functions of *K*, ∣*T*∣, and *R*.

- *Linear scaling inK*. For fixed ∣*T*∣, each client uploads a constant number of ciphertexts, so total uplink grows linearly in *K*.
- *Stepwise scaling in* ∣*T*∣. As ∣*T*∣ increases, *M* changes only when ∣*T*∣ crosses slot-capacity thresholds, producing staircase behavior in communication.
- *Shares scale withR*. Decryption-share traffic grows linearly in *R* and is independent of *K*.
- *Interleaving reduces communication*. Interleaving lowers the number of ciphertexts whenever ⌈2∣*T*∣/*B*⌉ < 2⌈∣*T*∣/*B*⌉, yielding up to a 2 × reduction in uplink and share traffic.
- *HE vs. plaintext*. The HE/Plain uplink ratio is constant in *K* but varies with ∣*T*∣, reflecting linear plaintext scaling versus stepwise HE scaling.

In the current prototype, encrypted alignment may be performed over the full grid *T*_all_. This affects measured byte counts in a live deployment but does not affect the formula-based communication results reported here, which are parameterized by ∣*T*∣.

Communication behavior follows directly from CKKS packing arithmetic. Total HE uplink scales linearly in *K* and stepwise in ∣*T*∣, while interleaving deterministically reduces communication by a factor *M*_sep_/*M*_int_ (up to 2 ×).

### Theoretical results

We provide (i) model identities and basic invariance results supporting RQ2–RQ3, (ii) CKKS perturbation bounds explaining numerical behavior (RQ2–RQ3), (iii) identifiability from public outputs (privacy context for RQ1), (iv) packing optimality (RQ4), and (v) communication and computational complexity laws (RQ5–RQ6).

We use the notation of Table 4. Throughout, we assume a fixed centralized multiset of rows is horizontally partitioned among sites (IID or non-IID, with or without overlap); that all parties use a shared alignment grid *T*_all_, derive the event-time subset *T* ⊆ *T*_all_, and apply the same tie convention; and that the Kaplan–Meier (KM) product only changes at event times, so using event times to form *T* is sufficient for the estimator, whereas using *T*_all_ merely refines the computational grid by inserting zero-event time points. In theorem statements, we write ${\widehat{S}}^{\star }(t)$ for the pooled Kaplan–Meier estimator computed from the aggregate counts on the event-time grid *T*; execution-mode-specific estimators carry explicit subscripts (${\hat{S}}_{\mathrm{plain}},\,{\hat{S}}_{\mathrm{HE}}-\mathrm{exact},\,{\hat{S}}_{\mathrm{HE}}-\mathrm{ckks}$). In the protocol section the released output is denoted ${\widehat{S}}_{{\rm{HE}}}(t)$.

### Model and basic identities

There are *K* sites, indexed by *k* ∈ {1,…,*K*}. Site *k* has a local set of unique observed survival times ${T}_{k}^{{\rm{all}}}$, including both event and censoring times. Let

$${T}_{{\rm{all}}}:=\mathop{\bigcup}\limits_{k=1}^{K}{T}_{k}^{{\rm{all}}}$$

denote the union of all unique survival times across sites. Let ${T}_{k}^{{\rm{event}}}\subseteq {T}_{k}^{{\rm{all}}}$ denote the unique event times at site *k*. The event-time grid used by the Kaplan–Meier estimator is

$$T:=\{t\in {T}_{\mathrm{all}}:\exists \,{\mathrm{an}}\,{\mathrm{even}}\,{\mathrm{tat}}\,t\}\,=\,\mathop{\bigcup }\limits_{k=1}^{K}{T}_{k}^{\mathrm{event}},$$

with cardinality ∣*T*∣.

The sets ${T}_{k}^{{\rm{event}}}$ and *T* are theoretical objects used in KM analysis and theorem statements. The implementation uploads only ${T}_{k}^{{\rm{all}}}$ and constructs only *T*_all_; the event-time subset *T* is handled implicitly because *d*_*i*_ = 0 at censoring-only time points and such factors do not alter the Kaplan–Meier product (Lemma 3).

For each *t*_*i*_ ∈ *T*, let ${d}_{i}^{(k)}$ denote the number of events at site *k* occurring at time *t*_*i*_, and let ${n}_{i}^{(k)}$ denote the number at risk at site *k* just before *t*_*i*_. Global counts at *t*_*i*_ are

$${d}_{i}=\mathop{\sum }\limits_{k=1}^{K}{d}_{i}^{(k)},\,{n}_{i}=\mathop{\sum }\limits_{k=1}^{K}{n}_{i}^{(k)}.$$

The KM estimator on the pooled aggregate counts is

$${\widehat{S}}^{\star }(t)=\mathop{\prod }\limits_{{t}_{i}\le t}\left(1-\frac{{d}_{i}}{{n}_{i}}\right),\,{n}_{i} > 0.$$

We write *h*_*i*_: = *d*_*i*_/*n*_*i*_ for the discrete hazard at *t*_*i*_ and

$$H(t):=\mathop{\sum }\limits_{{t}_{i}\le t}{h}_{i}$$

for the cumulative hazard evaluated on *T*.

When events and censorings occur at the same time *t*_*i*_, we adopt the standard Kaplan–Meier convention: the at-risk count *n*_*i*_ is taken just *beforet*_*i*_, events are applied at *t*_*i*_, and censorings at *t*_*i*_ affect only subsequent risk sets, not *d*_*i*_/*n*_*i*_ at *t*_*i*_.

Sites encrypt local contributions under a joint public key ${\mathsf{pk}}$. Conceptually, the server forms homomorphic sums of aligned local count vectors, and threshold fusion reconstructs packed plaintext blocks that decode to the aggregated counts. In the present implementation, the encrypted count vectors are aligned over *T*_all_; entries corresponding to times in *T*_all_⧹*T* have event count zero and therefore do not alter the Kaplan–Meier product. By Lemma 3, exact aggregation on *T*_all_ therefore yields the same KM estimator as exact aggregation on *T*.

CKKS with ring degree *n* packs *B* = *n*/2 complex slots per ciphertext; the RNS modulus chain is *Q* = ∏_*j*_*q*_*j*_ with bitlength $| Q| ={\sum }_{j}{\log }_{2}{q}_{j}$. We write ${q}_{\max }:=\mathop{\max }\limits_{j}{q}_{j}$ for the largest RNS limb modulus. A coarse size model is ct_bytes ≈ 2*n*∣*Q*∣/8 per ciphertext and share_bytes ≈ *n*∣*Q*∣/8 per partial share.

#### Lemma 1

(Global grid invariance) For any horizontal partition of a fixed centralized multiset of rows (IID or non-IID, with or without overlap), the union of all unique observed times

$${T}_{{\rm{all}}}=\mathop{\bigcup }\limits_{k=1}^{K}{T}_{k}^{{\rm{all}}}$$

is invariant. The derived event-time grid

$$T=\{\,t\in {T}_{{\rm{all}}}:\exists \,{\rm{an}}\,{\rm{event}}\,{\rm{at}}t\}$$

and its cardinality ∣*T*∣ are therefore also invariant.

#### Lemma 2

(Additivity of counts) For every *t*_*i*_ ∈ *T*, if local counting rules are aligned to the same global grid and tie convention, then

$${d}_{i}=\mathop{\sum }\limits_{k=1}^{K}{d}_{i}^{(k)}\,\mathrm{and}\,{n}_{i}=\mathop{\sum }\limits_{k=1}^{K}{n}_{i}^{(k)}.$$

#### Lemma 3

(Grid refinement invariance) Let *T* be the set of event times and let $\widetilde{T}\supseteq T$ be any refinement obtained by inserting time points with zero events. With consistent tie conventions, the KM estimator evaluated on $\widetilde{T}$ equals the KM estimator on *T* at all *t* ∈ *T*.

#### Theorem 4

(Plain federated KM equals pooled oracle) Running R1–R2 in plaintext yields ${\widehat{S}}_{{\rm{plain}}}(t)\equiv {\widehat{S}}^{\star }(t)$ for any partition of the same centralized multiset of rows.

#### Theorem 5

(Exact HE yields identical KM) If R2 uses exact homomorphic addition and threshold decryption returns the exact aggregated counts, then ${\widehat{S}}_{{\rm{HE}}}-{\rm{exact}}(t)\equiv {\widehat{S}}^{\star }(t)$ for any partition. This remains true whether alignment is performed directly on *T* or on any refinement $\widetilde{T}\supseteq T$, including *T*_all_.

For *proofs of Lemmas 1–3 and Theorems 4–5*, see Sections S1–S2 of the Supplementary Information.

### Approximate HE (CKKS): perturbation of the KM product

We model CKKS and threshold outputs on the event-time grid as ${\widehat{d}}_{i}={d}_{i}+\Delta {d}_{i}$ and ${\widehat{n}}_{i}={n}_{i}+\Delta {n}_{i}$ with deterministic error envelopes *ε*_*d*_(*i*) ≥ ∣Δ*d*_*i*_∣ and *ε*_*n*_(*i*) ≥ ∣Δ*n*_*i*_∣. The bound below follows from a multiplicative product inequality applied to the ratio ${\widehat{S}}_{{\rm{HE}}}-{\rm{ckks}}(t)/{\widehat{S}}^{\star }(t)$.

#### Theorem 6

(CKKS perturbation bound) Assume 0 ≤ *ε*_*n*_(*i*) < *n*_*i*_ (so ${\widehat{n}}_{i} > 0$); define *h*_*i*_ = *d*_*i*_/*n*_*i*_ and ${h}_{\max }=\mathop{\max }\limits_{{t}_{j}\le t}{h}_{j} < 1$. Set

$${\delta }_{i}\,:=\,\frac{{\varepsilon }_{d}(i)}{{n}_{i}-{\varepsilon }_{n}(i)}\,+\,\frac{{d}_{i}}{{n}_{i}}\cdot \frac{{\varepsilon }_{n}(i)}{\,{n}_{i}-{\varepsilon }_{n}(i)}.$$

Then, for all *t*,

$$\parallel {\widehat{S}}_{{\rm{HE}}}-{\rm{ckks}}(t)-{\widehat{S}}^{\star }(t)| \,\le {\widehat{S}}^{\star }(t)\left(\exp \left(\frac{1}{1-{h}_{\max }}\mathop{\sum }\limits_{{t}_{i}\le t}{\delta }_{i}\right)-1\right).$$

#### Remark 7

(Terminal jump with *h*_*i*_ = 1) If *d*_*i*_ = *n*_*i*_ at a terminal time point (so *h*_*i*_ = 1 and ${\widehat{S}}^{\star }$ drops to 0), the stated bound applies on indices with *h*_*j*_ < 1. At and after the terminal jump, the trivial bound $| {\widehat{S}}_{{\rm{HE}}}-{\rm{ckks}}(t)-{\widehat{S}}^{\star }(t)| ={\widehat{S}}_{{\rm{HE}}}-{\rm{ckks}}(t)$ holds.

#### Corollary 8

(Uniform convergence on *T*) If *ε*_*d*_(*i*), *ε*_*n*_(*i*) → 0 for all *t*_*i*_ ∈ *T*, then ${\widehat{S}}_{{\rm{HE}}}-{\rm{ckks}}(t)\to {\widehat{S}}^{\star }(t)$ uniformly on *T*.

For *proofs*, see Section S3 of the Supplementary Information.

### Identifiability from public outputs

#### Proposition 9

(Hazards are identified; counts are not) Assume *T* contains the ordered event times and is known, with $\widehat{S}(t_0)=1$. Then the per-time-point hazard ratios are determined by

$${h}_{i}\,=\,1-\frac{\widehat{S}({t}_{i})}{\widehat{S}({t}_{i-1})}, \qquad i=1,\ldots,m,\ \widehat{S}(t_{i-1})>0, \ \widehat{S}(t_0)=1.$$

However, without additional side information (e.g., initial at-risk size, censoring schedule), the integer pairs (*d*_*i*_, *n*_*i*_) consistent with these ratios are not unique; there exist infinitely many real-valued (*d*_*i*_, *n*_*i*_) solutions and, typically, multiple integer completions. Hence publishing $\widehat{S}(t)$ alone reveals *d*_*i*_/*n*_*i*_ but not (*d*_*i*_, *n*_*i*_) nor any per-site split.

For *proofs*, see Section S4 of the Supplementary Information.

### Noise envelopes and utility under a simple model

We separate exact identities above from implementation-dependent error scaling. The following assumption captures our add-only pipeline and the empirical behavior of share fusion.

#### Assumption 1

(Add-only CKKS noise and share aggregation) (i) For a ciphertext contributed by site *k* using *M*_*k*_ ciphertext blocks at fixed scale, the encode–encrypt perturbation satisfies $\parallel {\Delta }_{k}(\cdot )\parallel =O({M}_{k}\log {q}_{\max })$, where ${q}_{\max }:=\mathop{\max }\limits_{\ell }{q}_{\ell }$ is the largest RNS limb modulus. (ii) Threshold-share fusion aggregates zero-mean, independent share errors across decryptors with variance proxy *σ*^2^, contributing $O(\sigma \sqrt{R})$ to each block’s error.

In our protocol *M*_*k*_ = *M* for all *k* since all sites pack over the same grid *T*_all_; the propositions below are stated for the general case.

#### Proposition 10

(Utility envelope: coarse worst case) Under Assumption 1, there exist nonnegative *ε*_*d*_(*i*), *ε*_*n*_(*i*) with

$${\varepsilon }_{d}(i),{\varepsilon }_{n}(i)\in O\,\left(\log ({q}_{\max })\mathop{\sum }\limits_{k=1}^{K}{M}_{k}\right)+O(\sigma \sqrt{R})$$

for which

$$\Delta S(t):=\left|{\widehat{S}}_{\mathrm{oracle}}(t)-{\widehat{S}}_{\mathrm{HE}}(t)\right|\le \mathop{\prod }\limits_{{t}_{i}\le t}\left(1-\frac{{d}_{i}}{{n}_{i}}\right)\cdot \mathop{\sum }\limits_{{t}_{i}\le t}\frac{O\,\left(\log ({q}_{\max })\mathop{\sum }\limits_{k}{M}_{k}\right)+O(\sigma \sqrt{R})}{\,{n}_{i}-{\varepsilon }_{n}(i)},$$

reducing to the large-*n*_*i*_ form with denominator *n*_*i*_.

#### Proposition 11

(Bounded at-risk counts) If *n*_*i*_ ≥ *c* > 0 for all *t*_*i*_, then

$$\Delta S(t)\le \frac{1}{c}\mathop{\sum }\limits_{{t}_{i}\le t}\frac{O\,\left(\log ({q}_{\max })\mathop{\sum }\limits_{k}{M}_{k}\right)+O(\sigma \sqrt{R})}{1-\left(O\,\left(\log ({q}_{\max }){\sum }_{k}{M}_{k}\right)+O(\sigma \sqrt{R})\right)/{n}_{i}},$$

which further reduces to

$$\frac{1}{c}{\sum }_{{t}_{i}\le t}\left(O\,\left(\log ({q}_{\max }){\sum }_{k}{M}_{k}\right)+O(\sigma \sqrt{R})\right)$$

for large *n*_*i*_.

#### Proposition 12

(Error scaling with client count) Under Assumption 1,

$${\varepsilon }_{{\rm{total}}}=\mathop{\sum }\limits_{k=1}^{K}O({M}_{k}\log {q}_{\max })+O(\sigma \sqrt{R}).$$

For *proofs*, see Section S5 of the Supplementary Information.

### Packing optimality

Let *B* = *n*/2 be the CKKS slot budget and consider two aligned streams (*d*_•_, *n*_•_) of length *L* (in the implementation *L* = ∣*T*_all_∣; in the communication simulations of section “Results”, *L* = ∣*T*∣).

#### Lemma 13

(Packing lower bound and interleaving optimality) Any packing that avoids cross-ciphertext multiplies and carries both streams over length *L* requires at least $\lceil \frac{2L}{B}\rceil$ ciphertexts. Interleaved co-packing attains this bound with *M*_int_ = ⌈2*L*/*B*⌉, while separate packing uses *M*_sep_ = 2⌈*L*/*B*⌉ ≥ ⌈2*L*/*B*⌉.

*Proof idea:* pigeonhole principle on total required slots; see Section S6 of the Supplementary Information.

#### Corollary 14

(Interleaving advantage) Let *B* = *n*/2 be the CKKS slot budget and let *L* denote the length of the two streams (*d*_•_, *n*_•_). Separate packing uses *M*_sep_ = 2⌈*L*/*B*⌉ ciphertexts; interleaving uses *M*_int_ = ⌈2*L*/*B*⌉. Then *M*_int_ ≤ *M*_sep_ with *M*_sep_ − *M*_int_ ∈ {0, 1} and

$$\frac{{M}_{{\rm{sep}}}}{{M}_{{\rm{int}}}}\in [1,\,2].$$

More precisely: (i) $\frac{{M}_{{\rm{sep}}}}{{M}_{{\rm{int}}}}=2$ when *L* ≤ *B*/2; (ii) $\frac{{M}_{{\rm{sep}}}}{{M}_{{\rm{int}}}}=\frac{2c+2}{2c+1}\in (1,4/3]$ when *L* = *cB* + *r* with *c*≥1 and 0 < *r* ≤ *B*/2; and (iii) $\frac{{M}_{{\rm{sep}}}}{{M}_{{\rm{int}}}}=1$ when *L* = *cB* + *r* with *c* ≥ 1 and *B*/2 < *r* < *B*.

For *proofs*, see Section S6 of the Supplementary Information.

### Communication complexity

In R1 (plaintext), each site uploads its local unique observed-time set ${T}_{k}^{{\rm{all}}}$ and the coordinator broadcasts the resulting global alignment grid *T*_all_. In R2, the implementation uploads aligned at-risk and event counts over *T*_all_, giving 2*K*∣*T*_all_∣ scalars. An event-time-only variant that filters to the event-time subset *T* before uploading would use 2*K*∣*T*∣ scalars instead.

With CKKS packing (*B* = *n*/2 slots), a length-*L* vector packs into ⌈*L*/*B*⌉ ciphertexts. The formulas below use a generic packed length *L*: in the communication simulations (Figs. 7–9) *L* = ∣*T*∣; in the implementation *L* = ∣*T*_all_∣.

Under *separate* packing,

$${M}_{{\rm{sep}}}=2\left\lceil \frac{L}{B}\right\rceil ,$$

and under *interleaved* packing,

$${M}_{{\rm{int}}}=\left\lceil \frac{2L}{B}\right\rceil .$$

#### Theorem 15

(Communication: implemented plain federated KM) In the implemented protocol: R1 uplink: ${\sum }_{k=1}^{K}| {T}_{k}^{{\rm{all}}}|$ scalars;R1 downlink: *K* ∣*T*_all_∣ scalars;R2 uplink: 2*K* ∣*T*_all_∣ scalars. In an event-time-only variant where R2 counts are aligned over *T* instead of *T*_all_, R2 uplink reduces to 2*K* ∣*T*∣ scalars.

#### Theorem 16

(Communication: CKKS + threshold) If each client uploads *M* ∈ {*M*_sep_, *M*_int_} ciphertexts in R2 (the value of *M* is identical across sites since all pack over the same global alignment grid *T*_all_), the total R2 uplink is *KM* ciphertexts. Threshold decryption with a committee of size *R* requires *RM* partial shares, where *M* ∈ {*M*_sep_, *M*_int_} is the number of aggregated blocks to be decrypted. Equivalently,

$${M}_{{\rm{int}}}=\left\lceil \frac{2L}{B}\right\rceil ,\,{M}_{{\rm{sep}}}=2\left\lceil \frac{L}{B}\right\rceil ,$$

where *L* is the packed grid length.

#### Corollary 17

(Scaling laws) Implemented plain: *Θ*(*K*) for fixed ∣*T*_all_∣ and *Θ*(∣*T*_all_∣) for fixed *K*; in an event-time-only variant, the same asymptotics hold with ∣*T*∣ replacing ∣*T*_all_∣.CKKS: R2 uplink is linear in *K* and in ⌈*L*/*B*⌉; share traffic is linear in *R* and in ⌈*L*/*B*⌉, where *L* is the packed grid length. Interleaving satisfies ⌈2*L*/*B*⌉ ≤ 2⌈*L*/*B*⌉.

The communication simulations in section “Results” instantiate *L* = ∣*T*∣; the implementation uses *L* = ∣*T*_all_∣, and the same formulas apply.

For *proofs*, see Section S7 of the Supplementary Information.

### Computational complexity

#### Proposition 18

(Computational cost law) The per-round work satisfies

$$W({\rm{mode}})\,=\,\alpha K{M}_{\star }\,+\,\beta R{M}_{\star }\,+\,\gamma {M}_{\star }\,+\,{\delta }_{1}L\,+\,{\delta }_{2}| T| ,$$

where *M*_⋆_ = *M*_int_ or *M*_sep_ depending on packing, *L* is the packed grid length, and *α*, *β*, *γ*, *δ*_1_, *δ*_2_ > 0 are mode-independent constants. In the current implementation, *L* = ∣*T*_all_∣; in an event-time-only packing variant, *L* = ∣*T*∣.

The first term is homomorphic addition at the server; the second is partial-share creation by the committee; the third is fusion; the fourth captures packed-vector decode or alignment work on the packed grid; and the fifth is the Kaplan–Meier pass on the event-time grid. This mirrors the empirical near-linear trends in *K* and the interleaving advantage observed in section “Results”.

#### Corollary 19

(Runtime impact of interleaving) With *W*_sep_ and *W*_int_ the total work under separate vs. interleaved packing,

$$\frac{{W}_{{\rm{sep}}}}{{W}_{{\rm{int}}}}\,=\,\frac{(\alpha K+\beta R+\gamma ){M}_{{\rm{sep}}}+{\delta }_{1}L+{\delta }_{2}| T| }{(\alpha K+\beta R+\gamma ){M}_{{\rm{int}}}+{\delta }_{1}L+{\delta }_{2}| T| }\,\le \,\frac{{M}_{{\rm{sep}}}}{{M}_{{\rm{int}}}}\,\le \,2,$$

where the last inequality follows from Corollary 14. In the three regimes:

- If *L* ≤ *B*/2, then $\frac{{W}_{{\rm{sep}}}}{{W}_{{\rm{int}}}}\le 2$ (idealized 50% speedup upper bound).
- If (*M*_sep_, *M*_int_) = (4, 3), then $\frac{{W}_{{\rm{sep}}}}{{W}_{{\rm{int}}}}\le \frac{4}{3}$ (up to 25%).
- If *M*_sep_ = *M*_int_, packing does not change runtime.

Moreover, the fixed terms *δ*_1_*L* + *δ*_2_∣*T*∣ strictly reduce the realized ratio below *M*_sep_/*M*_int_ whenever they are positive.

For *proofs*, see Section S8 of the Supplementary Information.

## Discussion

Our framework advances privacy-preserving federated Kaplan–Meier (KM) analysis by adopting CKKS-based multiparty HE with multiparty decryption and strict output gating. Unlike integer-only systems^1^ that require fixed-point coordination for real workloads, CKKS natively supports approximate arithmetic over real values. This capability enables faithful time-to-event analytics while constraining privacy risk in multi-institution collaborations^2,6^.

We provide estimator-level guarantees including equality results for plaintext and homomorphic settings, a CKKS perturbation bound with uniform convergence, and an identifiability statement. These results are complemented by Kaplan–Meier specific communication and computational scaling laws together with a packing optimality result based on interleaving (*n*_*t*_, *d*_*t*_). Empirically, encrypted federated curves are numerically indistinguishable from pooled oracle estimates across survival, RMST, band coverage, supremum gap, and hazard diagnostics over large synthetic cohorts (up to *K* = 500) and on the NCCTG lung dataset (see Section S9 of the Supplementary Information); Fig. S1 (Supplementary Information-Fig. S3 (Supplementary Information)). Interleaved and separate packings match in accuracy, while interleaving reduces ciphertext count and therefore bandwidth and runtime in the regimes predicted by the packing arithmetic. Although HE introduces overhead relative to plaintext computation, end-to-end performance remains feasible for moderate and large federations. The resource model developed here provides concrete byte and memory envelopes that support practical deployment planning.

We quantify a subtraction-based reconstruction attack in plaintext two-round Kaplan–Meier protocols. A site can recover the residual contributions of other participants by subtracting its own $({n}_{t}^{(A)},{d}_{t}^{(A)})$ from broadcast totals $({n}_{t}^{({\rm{Fed}})},{d}_{t}^{({\rm{Fed}})})$. This leakage is exact when *K* = 2 and remains substantial for small overlapping coalitions. Our protocol removes this attack surface because per-time-point tables are never released. Only the survival curve ${\widehat{S}}_{{\rm{HE}}}(t)$ and optional confidence bands are revealed. By Proposition 9, publishing ${\widehat{S}}_{{\rm{HE}}}(t)$ identifies per-time-point hazard ratios *h*_*i*_ but does not determine integer totals (*d*_*i*_, *n*_*i*_) or any per-site decomposition. Consequently the subtraction channel is closed even for honest-but-curious participants.

Estimator fidelity is established both theoretically and empirically. Plain federated Kaplan–Meier equals the pooled oracle (Theorem 4). Exact homomorphic evaluation reproduces the oracle (Theorem 5). Approximate homomorphic evaluation under CKKS yields a perturbation bound for the Kaplan–Meier product together with uniform convergence as noise approaches zero (Theorem 6, Corollary 8). Empirical evaluation confirms that survival errors, RMST differences, coverage diagnostics, and hazard metrics remain within predefined tolerances.

Communication and computation follow simple laws specialized to Kaplan–Meier estimation. With slot capacity *B* = *n*/2, interleaved packing requires *M*_int_ = ⌈2*L*/*B*⌉ ciphertexts and separate packing requires *M*_sep_ = 2⌈*L*/*B*⌉, where *L* is the packed grid length. Encrypted uplink in Phase R2 is therefore linear in *K* and stepwise in *L*, while decryption share traffic is linear in committee size *R*. Computational effort decomposes into server additions *Θ*(*M*_⋆_*K*), share generation *Θ*(*RM*_⋆_), fusion *Θ*(*M*_⋆_), packed-grid decode or alignment work, and the final Kaplan–Meier pass *Θ*(∣*T*∣) on the event-time grid, where *M*_⋆_ ∈ {*M*_int_, *M*_sep_}. These scaling laws match the empirical behavior observed in section “Results” (the implementation uses *L* = ∣*T*_all_∣; the communication simulations use *L* = ∣*T*∣).

Our security posture reflects both the workload and the capabilities of current HE systems. We instantiate CKKS with parameters providing indistinguishability under chosen-plaintext attack (IND-CPA) under the RLWE assumption and enforce aggregate-only decryption through a multiparty committee. We do not claim IND-CCA2 security for CKKS. Instead we rely on operational safeguards including output gating, quorum-based multiparty decryption, authenticated transport, audit logging, and key rotation. These controls reduce practical risk in clinical data collaborations while preserving estimator fidelity.

In the present implementation, multiparty decryption shares are fused in an all-of-*R* configuration to recover aggregated counts required for Kaplan–Meier evaluation. If fusion is performed at the coordinator, the coordinator may transiently observe aggregated counts aligned over *T*_all_, including zero-event entries at censoring-only times. However, the coordinator never observes per-site contributions and only the final survival curve ${\widehat{S}}_{{\rm{HE}}}(t)$ is released to participating sites.

We deploy CKKS without decryption-time noise flooding in order to preserve numerical precision, achieving agreement with the pooled oracle at the 10^−8^ level. Instead of asserting IND-CPA^*D*^ security, we constrain potential oracle exposure operationally through four mechanisms. First, multiparty decryption is used with *θ* = *R*. Second, policy gating ties each computation to a precommitted cohort and alignment grid *T*_all_, from which the event-time grid *T* is derived. Third, the system exposes a restricted and non-adaptive interface with a bounded number of decryptions. Fourth, strict output gating ensures that only ${\widehat{S}}_{{\rm{HE}}}(t)$ and optional derived public summaries are published; the per-time-point aggregate table is not released.

Resource usage follows directly from the packing arithmetic. With (*n* = 16384, ∣*Q*∣ ≈ 438bits), one ciphertext occupies approximately 1.794 MB and one partial share approximately 0.897 MB. With slot capacity *B* = 8192, the ciphertext counts (*M*_int_, *M*_sep_) equal (1, 2) for packed length *L* = 1000, (2, 2) for *L* = 5000, and (3, 4) for *L* = 10,000. In the current implementation, *L* = ∣*T*_all_∣; in the communication simulations reported in section “Results”, *L* = ∣*T*∣. These values determine the bandwidth envelopes reported in the Results. Reliability improvements such as idempotent uploads, bounded retry strategies, reassignment of the lead role when a decryptor times out, and chunked transfers improve resilience on unreliable networks without altering the cryptographic design.

Operational parameters that influence deployment include the slot capacity *B* = *n*/2, packing layout, committee size *R*, threshold *θ*, and the choice of packed grid length *L*. Interleaved packing is generally preferable because it preserves accuracy while minimizing ciphertext count. Fixing a modest committee size, such as *R* = 9 or *R* = 25, decouples share traffic from federation size *K*. Selecting a ring degree *n* that places *L* on a favorable packing step can reduce ciphertext counts more than it increases per-ciphertext size. Under add-only Kaplan–Meier computation at scale 2^40^, numerical stability remains largely insensitive to the specific choices of *n*, *R*, and *θ*. Table 3 summarizes the recommended defaults. Table 4 summarizes the notation used in the federated CKKS Kaplan-Meier protocol.

**Table 3:** Recommended defaults for CKKS federated Kaplan–Meier computation with add-only aggregation

Knob	Default	Rationale/When to change
Ring degree *n*	16,384	Meets approximately 128-bit RLWE security with our ∣*Q*∣ while giving *B* = 8192 slots; often places the packed grid length *L* on favorable packing steps. Increase to 32,768 only if it moves *L* to a lower ciphertext plateau.
Packing	**Interleaved**	Co-pack (*n*_*t*_, *d*_*t*_) to minimize ciphertext count *M*; accuracy remains unchanged. Use *separate* only if downstream plaintext postprocessing requires distinct streams.
CKKS scale	2^40^	Preserves approximately 9 to 10 decimal digits under additions and rotations; stable across sites for add-only KM.
Modulus chain ∣*Q*∣	approximately 420 to 450 bits	Leveled use without bootstrapping. Provides headroom for additions and rotations at scale 2^40^.
Galois keys	Powers of two	Minimal set for slot rotations required by packing; no relinearization keys required.
Committee size *R*	*R* ∈ {9, 25}	Fix *R* independent of *K* to bound share traffic; *R* = 9 is lightweight and *R* = 25 provides a stronger quorum.
Threshold *θ*	*θ* = *R* (strict)	Maximizes confidentiality. Deployments supporting flexible thresholds may use *θ* = ⌈*ϕR*⌉ with *ϕ* ∈ [2/3, 0.7] to balance confidentiality and liveness.
Decryptors vs. clients	*R* ≤ *K*	Use all clients as decryptors (*R* = *K*) or a fixed subset; fixing *R* decouples share traffic from *K*.
Packed grid length *L*	*L* = ∣*T*_all_∣ (implementation); *L* = ∣*T*∣ (communication simulations)	The current implementation aligns encrypted counts over *T*_all_ (all observed times). The KM estimator depends only on event times *T*, since *d*_*t*_ = 0 for censoring-only times. Filtering to *T* before encryption reduces ciphertext count and communication when supported; the communication simulations in the “Results” section use *L* = ∣*T*∣.
Tie or censoring rule	Consistent across sites	Ensures additivity and partition-invariant Kaplan–Meier estimation.

**Table 4:** Notation used in the federated CKKS Kaplan–Meier protocol

Symbol	Meaning
*K*	Number of sites (clients).
${\mathcal{R}}$	Set of parties authorized to participate in threshold decryption.
*R*	Decryptor committee size, i.e., $R=| {\mathcal{R}}|$.
*r*	Index for an individual decryptor ($r\in {\mathcal{R}}$).
*n*	CKKS ring degree.
*B*	Slot capacity per ciphertext (*B* = *n*/2 for CKKS).
∣*Q*∣	Total modulus bitlength controlling ciphertext and share sizes.
*q*_*j*_	The *j*-th RNS limb modulus; ${q}_{\max }:=\mathop{\max }\limits_{j}{q}_{j}$.
*T*_all_	Global alignment grid: union of all unique observed survival times across sites (including event and censoring times).
$T={\{{t}_{i}\}}_{i=1}^{| T| }$	Event-time grid: subset of *T*_all_ containing only times with at least one observed event; ∣*T*∣ denotes its cardinality.
${T}_{k}^{{\rm{all}}}$	Local unique survival times at site *k* (including event and censoring times).
${T}_{k}^{{\rm{event}}}$	Local unique event times at site *k*; satisfies ${T}_{k}^{{\rm{event}}}\subseteq {T}_{k}^{{\rm{all}}}$. Theoretical; the implementation does not upload ${T}_{k}^{{\rm{event}}}$ separately (see section “Model and basic identities”).
${d}_{i}^{(k)}$	Site-*k* event count at time *t*_*i*_ (number of rows with *t* = *t*_*i*_ and *e* = 1).
${n}_{i}^{(k)}$	Site-*k* at-risk count just before *t*_*i*_ (number of rows with *t*≥*t*_*i*_).
*d*_*i*_, *n*_*i*_	Aggregated event and at-risk counts at time *t*_*i*_.
*L*	Generic packed grid length appearing in layout, communication, and runtime formulas; instantiated explicitly in each section.
*M*_0_	Per-stream ciphertext count, *M*_0_ = ⌈*L*/*B*⌉.
*M*_int_	Ciphertext count under interleaved packing, *M*_int_ = ⌈2*L*/*B*⌉.
*M*_sep_	Total ciphertext count under separate packing, *M*_sep_ = 2*M*_0_ = 2⌈*L*/*B*⌉.
${\mathcal{I}}$	Public ciphertext-index set induced by the packing layout: ${\mathcal{I}}=[{M}_{{\rm{int}}}]$ for interleaving, ${\mathcal{I}}=[{M}_{0}]\times \{d,n\}$ for separate packing.
${{\bf{z}}}_{\iota }^{(k)}$	Packed plaintext block at index $\iota \in {\mathcal{I}}$ for site *k*. For interleaving, ι = *j* indexes interleaved blocks; for separate packing, ι = (*j*, *s*) pairs a block index *j* ∈ [*M*_0_] with a stream label *s* ∈ {*d*, *n*}. We treat count vectors as real-valued slot payloads embedded in the CKKS plaintext space over *B* complex slots.
${\mathsf{pk}}$	Joint public key produced by distributed key generation.
${{\mathsf{sk}}}_{k}$	Secret-key share held by site *k*.
${E}_{{\mathsf{pk}}}(\cdot )$	Public-key encryption under ${\mathsf{pk}}$ (CKKS).
${{\mathsf{ct}}}_{\iota }^{(k)}$	Ciphertext block uploaded by site *k* at public index $\iota \in {\mathcal{I}}$.
${{\mathsf{ct}}}_{\Sigma ,\iota }$	Aggregated ciphertext block at public index $\iota \in {\mathcal{I}}$: homomorphic sum ${\bigoplus }_{k=1}^{K}{{\mathsf{ct}}}_{\iota }^{(k)}$.
${\Delta }_{\iota }^{(r)}$	Partial decryption share produced by decryptor *r* for aggregated block ${{\mathsf{ct}}}_{\Sigma ,\iota }$.
*pt*_ι_	Fused plaintext block after MULTIPARTYDECRYPTFUSION at index ι.
**u**_ι_	Decoded slot vector extracted from *pt*_ι_.
${\widehat{S}}^{\star }(t)$	Pooled Kaplan–Meier estimator on aggregate counts; used as the target in theorem statements.
${\widehat{S}}_{{\rm{oracle}}}(t)$	Synonym for ${\widehat{S}}^{\star }(t)$, used in experimental metrics and figures.
${\widehat{S}}_{{\rm{plain}}}(t)$	Plaintext federated Kaplan–Meier estimator (Theorem 4).
${\widehat{S}}_{{\rm{HE}}}(t)$	HE-federated Kaplan–Meier estimator; subscripted as ${\widehat{S}}_{{\rm{HE}}}-{\rm{exact}}$ for exact HE (Theorem 5) and ${\widehat{S}}_{{\rm{HE}}}-{\rm{ckks}}$ for CKKS approximate HE (Theorem 6).
*#*{ ⋅ }	Set cardinality operator.
*θ*	Decryption threshold (minimum number of decryptors required for fusion); in our implementation and experiments *θ* = *R*.
⨁	Homomorphic addition in CKKS (ciphertext addition corresponding to slotwise plaintext addition).

Real-world multi-institutional deployments may experience network instability or slow participants (“stragglers”). The protocol structure naturally limits the impact of such delays. Phase B aggregation requires only a single ciphertext upload from each site, so network latency affects only the completion time of the aggregation round rather than its correctness. Because aggregation consists solely of homomorphic additions, the coordinator can accumulate ciphertexts incrementally as they arrive and defer Phase C until all uploads are complete.

Phase C decryption requires contributions from all *R* decryptors in the current all-of-*R* configuration (*θ* = *R*). In practice this stage can be made resilient to stragglers using standard distributed systems techniques such as bounded retry policies, checkpointing, and temporary lead reassignment among decryptors. A deployment requiring stronger liveness guarantees could instead adopt a *θ*-of-*R* configuration with *θ* < *R*, allowing fusion once a quorum of decryptors has responded. These operational mechanisms do not change the communication or security analysis presented in this work but improve robustness in heterogeneous network environments.

Secure multi-party computation (MPC) protocols provide an alternative framework for privacy-preserving survival analysis. In MPC-based Kaplan–Meier implementations, sites jointly compute the survival function using secret sharing and interactive protocols ^7^. While MPC offers strong cryptographic guarantees, it typically requires multiple rounds of interaction among participating sites, with the number of rounds depending on the multiplicative depth of the computation ^8^.

In contrast, the HE approach used here requires a single encrypted upload from each site (Phase B), followed by server-side homomorphic aggregation and a single round of partial decryption shares from the committee (Phase C). The total number of communication rounds is therefore small and does not grow with the number of participating sites. This simplifies deployment across institutions with heterogeneous connectivity.

Furthermore, CKKS natively supports approximate arithmetic over real values, which aligns well with statistical estimators such as Kaplan–Meier and restricted mean survival time (RMST). MPC-based solutions often require fixed-point representations and additional coordination to maintain numerical consistency across sites.

MPC-based approaches do offer complementary strengths. They can provide exact arithmetic with no approximation error, may support stronger security models that avoid assumptions related to approximate decryption in CKKS ^9^, and can be designed without a trusted aggregation coordinator. For settings where exact counts are required or where the coordinator cannot be assumed honest-but-curious, an MPC design may therefore be preferable.

For the federated survival analysis setting considered here, however, the CKKS approximation error on the survival curve ${\widehat{S}}_{{\rm{HE}}}(t)$ evaluated at event times remains below 10^−8^ across all experimental configurations (section “Results”), well within typical clinical reporting tolerances. Under the threat model adopted in section “Threat model, threshold, and key management”, and with output gating ensuring that only ${\widehat{S}}_{{\rm{HE}}}(t)$ is released to sites, the threshold HE design provides a practical balance between security, numerical fidelity, scalability, and operational simplicity for large federations up to *K* = 500 sites.

Several limitations remain. The protocol currently focuses on add-only encrypted aggregation for Kaplan–Meier estimation. Extensions to weighted estimators, interval censoring, or Cox-type models will require careful management of multiplicative depth or hybrid techniques combining HE with differential privacy. Additional directions include verifiable aggregation to protect against malicious coordinators, deployments supporting flexible *θ*-of-*R* thresholds under participant churn, and broader stress testing across heterogeneous institutional networks. Together these directions extend the foundation established here for secure and scalable multi-institution survival analysis.

## Methods

### Notation and preliminaries

*Notation* Throughout the paper, *T*_all_ denotes the global alignment grid of all distinct observed times, *T* ⊆ *T*_all_ denotes the event-time grid used by the Kaplan–Meier product, and *L* denotes a generic packed grid length. In the implementation, *L* = ∣*T*_all_∣; in an event-time-only packing variant, *L* = ∣*T*∣. Each section that uses *L* states the instantiation explicitly.

For ciphertext layout, we write

$${M}_{0}:=\lceil L/B\rceil ,\,{M}_{{\rm{int}}}:=\lceil 2L/B\rceil ,\,{M}_{{\rm{sep}}}:=2{M}_{0}.$$

We let ${\mathcal{I}}$ denote the public ciphertext-index set induced by the chosen layout: ${\mathcal{I}}=[{M}_{{\rm{int}}}]$ for interleaving and ${\mathcal{I}}=[{M}_{0}]\times \{d,n\}$ for separate packing. For interleaving, each $\iota \in {\mathcal{I}}$ is a block index; for separate packing, each ι = (*j*, *s*) pairs a block index *j* ∈ [*M*_0_] with a stream label *s* ∈ {*d*, *n*}.

In the implementation, ${\mathcal{R}}=[K]$ and hence *R* = *K*. When studying communication scaling, we also consider hypothetical settings with fixed committee size *R* independent of *K*.

Modern Learning With Errors (LWE) and Ring Learning With Errors (RLWE) based encryption schemes use noisy ciphertexts together with a linear-style decryption relation determined by a secret key. In threshold variants, the secret key is distributed into shares so that no single party can complete decryption alone; instead, each decryptor contributes a *partial decryption share*, and a combiner fuses a qualified set of shares to recover the plaintext. General constructions and security proofs for threshold LWE/RLWE-style encryption appear in Asharov et al.^10^ and Cramer et al.^11^.

The protocol notation in this work allows a general *θ*-of-*R* threshold among decryptors. In our implementation, however, we instantiate the strict all-of-*R* configuration *θ* = *R* using OpenFHE’s multiparty Lead/Main/Fusion APIs. Supporting a more general *θ*-of-*R* threshold is orthogonal to the Kaplan–Meier pipeline itself and depends on the underlying multiparty HE configuration rather than on the survival-analysis logic.

We use CKKS for approximate packed arithmetic on real-valued vectors. The later security analysis treats the protocol phase by phase. In particular, the Phase B ciphertext privacy claim is stated for the distributed threshold setting used here, where an adversary may already hold a strict subset of secret-key shares from the distributed key-generation phase. For that reason, the formal Phase B privacy theorem is stated under an explicit threshold-aware ciphertext privacy assumption rather than under vanilla single-key IND-CPA alone.

We distinguish between the full alignment grid *T*_all_ and the event-time grid *T* ⊆ *T*_all_. The protocol and current implementation align encrypted counts on *T*_all_, while the Kaplan–Meier estimator depends only on event times in *T*. Inserting time points with zero events does not change the estimator (Lemma 3), so this distinction preserves consistency between theory and implementation.

### Federated KM with threshold CKKS: protocol and phases

We use CKKS^6^ with batching to support approximate real arithmetic on packed vectors. Our protocol organizes the federated Kaplan–Meier workflow into three phases that separate *key setup*, *encrypted aggregation*, and *threshold decryption with output gating*. The coordinator orchestrates the round structure and performs add-only homomorphic aggregation; sites compute local per-time Kaplan–Meier counts aligned to the shared global alignment grid *T*_all_ and encrypt them under a joint public key, while the final Kaplan–Meier product is evaluated on the event-time subset *T* ⊆ *T*_all_; a decryptor committee contributes partial decryption shares that are fused into aggregate plaintexts from which the public survival output is computed. Algorithms 1–3 formalize the steps, and Table 5 groups the protocol keywords by phase.

**Table 5:** Protocol keywords grouped by phase

Keyword	Phase	Role	Purpose
InitCKKS	A	Coordinator	Initialize CKKS parameters and context (Alg. 1, l. 1).
ChooseOrder	A	Coordinator	Fix deterministic DKG order of sites (Alg. 1, l. 2).
KeyGen	A	First site	Create the initial public key and the first secret-key share (Alg. 1, l. 6).
MultipartyKeyGen	A	Each subsequent site	Mix in the site’s secret share to update the joint ${\mathsf{pk}}$; each site keeps its local ${{\mathsf{sk}}}_{k}$ (Alg. 1, l. 8).
Broadcast(${\mathsf{pk}}$)	A	Coordinator	Distribute the joint public key to all sites (Alg. 1, l. 11).
UniqueTimes	A	Each site	Extract and upload local unique observed survival times ${T}_{k}^{{\rm{all}}}$ (Alg. 1, l. 12).
Union & Sort	A	Coordinator	Form and sort the global alignment grid ${T}_{{\rm{all}}}={\bigcup }_{k}{T}_{k}^{{\rm{all}}}$ and broadcast it to all sites (Alg. 1, l. 12). The event-time subset *T* = {*t* ∈ *T*_all_: ∃ aneventat*t*} is not constructed explicitly; it is handled implicitly during KM evaluation because *d*_*i*_ = 0 at censoring-only times.
Pack (interleaved)	B	Site *k*	Fill slots as [*n*_1_, *d*_1_, *n*_2_, *d*_2_,…] with *M* = ⌈2∣*T*_all_∣/*B*⌉ (Alg. 2, ll. 6–7).
Pack (separate)	B	Site *k*	Pack [*d*_•_] and [*n*_•_] separately; *M* = 2⌈∣*T*_all_∣/*B*⌉ (Alg. 2, ll. 9–10).
Enc/${E}_{{\mathsf{pk}}}(\cdot )$	B	Site *k*	Encrypt packed vectors under the joint public key (Alg. 2, ll. 8,11).
Agg: Add	B	Coordinator	Perform slotwise homomorphic addition (Alg. 2, ll. 14–20).
MultipartyDecryptLead	C	One decryptor	Produce the lead partial decryption share (Alg. 3, l. 4).
MultipartyDecryptMain	C	Other decryptors	Produce the remaining partial decryption shares (Alg. 3, l. 6).
MultipartyDecryptFusion	C	Combiner	Fuse the collected valid shares; in our implementation this is all-of-*R* (Alg. 3, l. 9).
GetCKKSPackedValue	C	Combiner	Decode CKKS plaintext slots (Alg. 3, l. 10).
ReconstructCounts	C	Combiner	Reconstruct the aggregate table ${\{({n}_{i},{d}_{i})\}}_{i=1}^{L}$ from fused plaintext blocks (Alg. 3).
ComputeKM	C	Combiner	Evaluate the Kaplan–Meier estimator on the alignment grid *T*_all_ from the reconstructed aggregate table (equivalent to evaluation on the event-time subset *T* since *d*_*i*_ = 0 at non-event times; Alg. 3).
Reveal	C	Coordinator	Release only the final survival curve ${\widehat{S}}_{{\rm{HE}}}(t)$ (and optional public summaries), not the per-time-point aggregate table (Alg. 3).
Broadcast(${\widehat{S}}_{{\rm{HE}}}$)	C	Coordinator	Disseminate the final public output (Alg. 3).

Phase A performs setup and time-grid construction; Phase B performs encrypted aggregation; Phase C performs multiparty decryption and output reveal. The keywords map to the abstract protocol tuple as follows: SETUP ↔ Phase A (InitCKKS through Union & Sort); PACK + ENC + AGG ↔ Phase B; PARTDEC ↔ MultipartyDecryptLead/Main; FUSE ↔ MultipartyDecryptFusion; REVEAL ↔ ReconstructCounts + ComputeKM + Reveal + Broadcast(${\widehat{S}}_{{\rm{HE}}}$).

The protocol description and the security claims address different aspects of the system. In particular, Phase B uses CKKS encryption under a joint public key, but the formal privacy theorem for distributed encrypted aggregation is stated under the threshold-aware auxiliary-input ciphertext privacy assumption introduced in section “Security analysis by phase”, rather than being derived here directly from a standalone vanilla IND-CPA theorem for single-key CKKS.

Let ${\mathsf{Params}}$ denote the CKKS parameter set. The protocol

$${\Pi }_{{\rm{KM}}-{\rm{Fed}}-{\rm{CKKS}}}=\left({\rm{S}}{\rm{ETUP}},{\rm{P}}{\rm{ACK}},{\rm{E}}{\rm{NC}},{\rm{A}}{\rm{GG}},{\rm{P}}{\rm{ART}}{\rm{D}}{\rm{EC}},{\rm{F}}{\rm{USE}},{\rm{R}}{\rm{EVEAL}}\right)$$

comprises: SETUP (Phase A), PACK+ENC+AGG (Phase B), and PARTDEC+FUSE+REVEAL (Phase C). The protocol notation allows a general *θ*-of-*R* threshold among decryptors, but in our implementation and experiments we instantiate the strict all-of-*R* setting *θ* = *R*. Only the released survival curve ${\widehat{S}}_{{\rm{HE}}}(t)$ (and optional derived summaries such as confidence bands, if computed) is published to sites.

#### Algorithm 1

Phase A: Key Initialization and Alignment Grid 

#### Algorithm 2

Phase B: Encrypted Survival Counts & Server Aggregation 

#### Algorithm 3

Phase C: Multiparty Decryption & Output Gating 

*Phase A* performs key initialization and construction of the global alignment grid. The coordinator initializes the CKKS context, fixes a deterministic site order *π*, and runs iterative multiparty key generation: the first site calls KEYGEN, and each subsequent site calls MULTIPARTYKEYGEN to incorporate its contribution into the joint public key. Each site retains its own secret-key share ${{\mathsf{sk}}}_{k}$; the coordinator learns only the resulting joint public key ${\mathsf{pk}}$. Measured in *K*, this phase performs one KEYGEN, *K* − 1 MULTIPARTYKEYGEN calls, and one broadcast.

In the same phase, each site uploads its local unique observed survival times ${T}_{k}^{{\rm{all}}}$ (including both event and censoring times). The coordinator forms the global alignment grid by union:

$${T}_{\mathrm{all}}={\mathrm{Sort}}\,\left(\mathop{\bigcup}\limits_{{\rm{k}}=1}^{{\rm{K}}}{T}_{k}^{\mathrm{all}}\right).$$

The event-time subset *T* = {*t* ∈ *T*_all_: ∃ aneventat*t*} is not constructed explicitly in the deployed pipeline; it is handled implicitly during aligned counting and KM evaluation, since *d*_*i*_ = 0 at censoring-only time points and such factors do not alter the Kaplan–Meier product (Lemma 3). The protocol operates over *T*_all_ for alignment and encrypted aggregation, while the Kaplan–Meier estimator depends only on the event-time subset *T* in theoretical statements.

The time-grid union runs in plaintext and reveals *T*_all_ by design. Deployments that require hiding local time sets ${T}_{k}^{{\rm{all}}}$ would need to replace the plaintext union with a private-set-union or related encrypted set-operation primitive.

*Phase B* performs encrypted aggregation of Kaplan–Meier counts. Given the shared alignment grid *T*_all_ and joint public key ${\mathsf{pk}}$, each site computes local event counts ${d}_{i}^{(k)}$ and at-risk counts ${n}_{i}^{(k)}$ at each *t*_*i*_ ∈ *T*_all_, packs them into CKKS plaintext blocks, encrypts the packed vectors, and uploads the resulting ciphertexts. For non-event times, ${d}_{i}^{(k)}=0$, so inclusion of these indices does not affect the Kaplan–Meier product.

In the *interleaved* layout, slots are packed as [*n*_1_, *d*_1_, *n*_2_, *d*_2_, …], giving

$${M}_{\mathrm{int}}=\left\lceil \frac{2|{T}_{\mathrm{all}}|}{B}\right\rceil ,\,B=\frac{n}{2}.$$

In the *separate* layout, the *d*_•_ and *n*_•_ streams are packed independently, giving

$${M}_{\mathrm{sep}}=2\left\lceil \frac{|{T}_{\mathrm{all}}|}{B}\right\rceil .$$

The coordinator performs only elementwise homomorphic addition to form aggregate ciphertext blocks. Server work is *O*(*MK*) homomorphic additions, and per-site uplink is *Θ*(*M*). No plaintext is revealed in this phase. The formal privacy guarantee for this phase is given later as an assumption-based ciphertext-content indistinguishability result in the distributed threshold setting.

*Phase C* performs multiparty decryption and output gating. The aggregated ciphertext blocks are sent to the decryptor committee. In the current implementation, all *R* decryptors participate, so fusion is all-of-*R*. For each block *j*, exactly one decryptor produces the lead share via MULTIPARTYDECRYPTLEAD, and the remaining decryptors produce main shares via MULTIPARTYDECRYPTMAIN. The combiner calls MULTIPARTYDECRYPTFUSION once per block to obtain plaintext block *pt*_*j*_, decodes the CKKS slots, and reconstructs the aggregate count table

$${\{({n}_{i},{d}_{i})\}}_{i=1}^{| {T}_{{\rm{all}}}| }.$$

It then evaluates the Kaplan–Meier estimator over the event-time grid *T*:

$${\widehat{S}}_{{\rm{HE}}}(t)=\mathop{\prod }\limits_{\begin{array}{c}{t}_{i}\le t\\ {t}_{i}\in T\end{array}}\left(1-\frac{{d}_{i}}{{n}_{i}}\right).$$

Because *d*_*i*_ = 0 at non-event times, evaluating the product over *T* is equivalent to evaluating it over *T*_all_.

Only ${\widehat{S}}_{{\rm{HE}}}(t)$, and optionally derived public summaries, is returned to sites; the aggregate count table is not released by the protocol interface. Costs scale as *O*(*RM*) partial-share computations, *O*(*M*) fusions, and *O*(∣*T*∣) for the Kaplan–Meier pass.

If the combiner is co-located with the coordinator, then the coordinator may transiently observe the aggregate plaintext counts (*n*_*i*_, *d*_*i*_) needed to evaluate ${\widehat{S}}_{{\rm{HE}}}(t)$. This does not change what is *released* to sites, but it does enlarge the coordinator’s transient operational visibility and is therefore treated explicitly in the later deployment qualification of the security analysis.

### Threat model, threshold, and key management

The system assumes a semi-honest coordinator and semi-honest sites. All parties follow the protocol as specified but may attempt to infer additional information from the transcripts they observe. Transport is protected by mutually authenticated, encrypted channels. Malicious deviations, adaptive corruption, and coordinator-site collusion are outside the present model and would require additional mechanisms and a separate analysis.

Sites perform distributed key generation as follows: the first site calls KEYGEN, and each subsequent site calls MULTIPARTYKEYGEN, producing a joint public key ${\mathsf{pk}}$ while each site retains its own secret-key share ${{\mathsf{sk}}}_{k}$. The protocol notation allows a general *θ*-of-*R* decryption threshold, where *θ* is the minimum number of decryptors required for successful fusion. In our implementation and experiments we instantiate the strict all-of-*R* setting *θ* = *R*, meaning all decrypting parties contribute shares before decryption completes.

Phase A reveals public alignment metadata: each site discloses its local unique observed survival times, from which the coordinator forms the global alignment grid *T*_all_. The event-time grid *T* is a theoretical subset of *T*_all_ and is not constructed explicitly in the implementation. The alignment grid is treated as non-sensitive and is broadcast to all sites.

The decryptor committee is intended to enforce an *aggregate-only* decryption policy bound to a precommitted cohort and alignment grid *T*_all_, from which the event-time grid *T* is derived. In particular, the intended interface does not offer a general adaptive decryption oracle. Decryption is restricted to ciphertexts produced by the fixed aggregation pipeline, tied to the committed cohort and grid, and used only to recover the aggregate plaintext required for the Kaplan–Meier output. This restriction is important because our formal Phase C claim is output-relative: it characterizes what a corrupted decryptor learns relative to the fused plaintext and the released curve, rather than asserting a generic adaptive chosen-ciphertext guarantee for CKKS.

The following deployment controls are recommended:

(i) time- or usage-based key rotation with fresh DKG;
(ii) authorization logs binding each decryption request to the committed cohort and alignment grid *T*_all_;
(iii) attestation and audit of decryptor hosts; and
(iv) mutually authenticated encrypted transport for all protocol traffic.

If the combiner operates within the decryptor committee and is not co-located with the coordinator, then the coordinator never sees aggregate plaintext counts. If the combiner is co-located with the coordinator, then the coordinator may transiently observe the aggregate plaintext table ${\{({n}_{i},{d}_{i})\}}_{i=1}^{| {T}_{{\rm{all}}}| }$ required for Kaplan–Meier evaluation; however, only the final survival curve ${\widehat{S}}_{{\rm{HE}}}(t)$ is released to sites.

### Security model and claims

We analyze

$${\Pi }_{\mathrm{KM}-\mathrm{Fed}-\mathrm{CKKS}}$$

in a *semi-honest* (honest-but-curious), *static* adversarial model. Corruption sets are fixed before protocol execution begins, and corrupted parties follow the protocol faithfully while attempting to infer additional information from their observed transcripts. The view of a corrupted party includes its local input, its full random tape, and all messages it receives. Adaptive corruption and malicious deviations are outside the scope of this analysis.

The coordinator is modeled as a semi-honest, non-colluding aggregator: it performs homomorphic operations faithfully but may attempt to infer information from ciphertexts and metadata. Collusion between the coordinator and corrupted sites would enlarge the adversarial view and is excluded from the present model. Transport is assumed to be protected by mutually authenticated, encrypted channels.

Let ${{\mathcal{C}}}_{S}\subsetneq [K]$ denote the set of corrupted sites. Let ${\mathcal{R}}\subseteq [K]$ denote the set of parties authorized to participate in threshold decryption, with $R=| {\mathcal{R}}|$ and decryption threshold *θ*. In our implementation and experiments, every site participates in decryption, so ${\mathcal{R}}=[K]$ and we instantiate the strict all-of-*R* setting *θ* = *R*. Let ${{\mathcal{C}}}_{R}\subsetneq {\mathcal{R}}$ denote the set of corrupted decryptors. The corruption sets ${{\mathcal{C}}}_{S}$ and ${{\mathcal{C}}}_{R}$ are indexed over different protocol roles and may overlap.

Standard IND-CPA for CKKS governs ciphertext indistinguishability under chosen plaintext attack, but does not cover a setting in which an adversary can issue adaptive decryption queries. In principle, approximate decryption behavior can become relevant under stronger oracle models. Rather than claiming a generic adaptive decryption guarantee for CKKS, our protocol constrains the oracle surface operationally:

(i) no single party can decrypt aggregated ciphertexts on its own;
(ii) decryption is restricted to ciphertexts bound to a precommitted cohort and alignment grid *T*_all_;
(iii) the computation interface is fixed to add-only aggregation followed by one Kaplan–Meier evaluation pass; and
(iv) only the released survival output ${\widehat{S}}_{{\rm{HE}}}(t)$ is published to sites, while the aggregated plaintext table ${\{({n}_{i},{d}_{i})\}}_{i=1}^{| {T}_{{\rm{all}}}| }$ remains internal to the decryption workflow unless the deployment explicitly co-locates fusion with the coordinator.

Accordingly, the formal Phase C claim below is *simulation relative to the fused plaintext and released output*, not a generic adaptive decryption security statement. The Kaplan–Meier estimator itself depends only on the event-time subset *T* ⊆ *T*_all_, since *d*_*i*_ = 0 at censoring-only time points.

Our formal guarantees are phase-specific and transcript-based.

- (C1) **Phase A setup privacy**. The distributed key-generation transcript is simulatable from the corrupted parties’ local DKG state and the final global alignment grid *T*_all_ and event-time grid *T*. This does *not* hide the plaintext union steps beyond what is revealed by *T*_all_ and *T* themselves.
- (C2) **Phase B encrypted aggregation privacy**. The encrypted aggregation transcript is computationally indistinguishable from a simulation that does not use the honest sites’ local Kaplan–Meier count vectors, *assuming* threshold-aware auxiliary-input ciphertext privacy for the distributed CKKS layer. This is an assumption-based claim; we do not prove that assumption from RLWE for our specific threshold CKKS instantiation in this work.
- (C3) **Phase C output-relative privacy**. The decryption transcript seen by a corrupted decryptor is simulatable from the aggregate plaintext table *P*, the signed metadata ${\mathsf{md}}$, the released curve ${\widehat{S}}_{{\rm{HE}}}(t)$, and the corrupted decryptors’ local state, under the stated threshold decryption assumptions. This is a protocol-level reduction to the underlying threshold decryption mechanism, not a derivation from RLWE alone.
- (C3) **Output gating**. Only ${\widehat{S}}_{{\rm{HE}}}(t)$ is released to sites. The aggregated plaintext table $P={\{({n}_{i},{d}_{i})\}}_{i=1}^{L}$ is not released by the protocol interface. This closes the direct subtraction channel that would arise if per-time aggregate counts were published in plaintext.

The formal statements proved in section “Security analysis by phase” establish:

(i) identical-distribution simulation for the DKG transcript in Phase A, relative to the exposed alignment grid *T*_all_ and event-time grid *T*;
(ii) computational indistinguishability of the Phase B encrypted aggregation transcript from a zero-encryption simulation, under Assumption 2; and
(iii) computational indistinguishability of the Phase C decryption transcript from a simulation relative to the fused plaintext and released output, under the threshold-decryption assumptions in Theorem 22.

These are the precise claims supported by the proofs. In particular, the Phase B claim is not a standalone theorem about vanilla CKKS IND-CPA alone; it is a theorem about our distributed setting under Assumption 2. Likewise, the Phase C claim is output-relative and committee-relative, not a statement that the decryption phase hides the fused plaintext from committee members who legitimately participate in fusion.

Our security analysis focuses on confidentiality and transcript privacy in the semi-honest model. Authenticity and integrity are provided operationally by the deployment assumptions: mutually authenticated channels, per-message integrity protection, and signed metadata binding each decryption request to its cohort, alignment grid *T*_all_, and ciphertext identifiers. We do not prove cryptographic soundness against an actively malicious coordinator or malicious sites in this work. Preventing malformed ciphertext uploads, replayed shares, or incorrect aggregation under malicious deviations would require additional mechanisms such as ciphertext validity checks, verifiable aggregation, and verifiable threshold decryption.

The guarantees above are transcript-based and do not eliminate all side channels. In particular:

(i) the plaintext union step in Phase A reveals the global alignment grid *T*_all_; the theoretical event-time grid *T* ⊆ *T*_all_ is not transmitted explicitly in the implementation but is included in the security model for theoretical generality;
(ii) metadata such as ciphertext count, ciphertext size, upload timing, and committee size may reveal coarse-grained system information;
(iii) the current analysis excludes coordinator-site collusion; and
(iv) if fusion is co-located with the coordinator, then the coordinator may transiently observe the aggregate plaintext table ${\{({n}_{i},{d}_{i})\}}_{i=1}^{| {T}_{{\rm{all}}}| }$ needed to evaluate ${\widehat{S}}_{{\rm{HE}}}(t)$, even though only ${\widehat{S}}_{{\rm{HE}}}(t)$ is released to sites.

These limitations are reflected explicitly in the phase-wise theorems and in the deployment qualifications stated there.

Under the stated semi-honest, static model, our protocol provides: (i) setup privacy relative to the intentionally exposed alignment grid *T*_all_ and event-time grid *T*; (ii) assumption-based ciphertext content privacy during encrypted aggregation; and (iii) output-relative privacy during threshold decryption and release. Together, these characterize what the protocol hides, what it intentionally reveals, and what remains dependent on deployment choices and system-level controls.

### Security analysis by phase

Let

$$\Pi :={\Pi }_{{\rm{KM}}-{\rm{Fed}}-{\rm{CKKS}}}$$

denote the protocol from section “Methods”. Let *λ* denote the security parameter throughout. We analyze the protocol phase by phase, since the three phases have different security goals and rely on different assumptions.

The guarantees proved below are intentionally phase-specific.

(i) **Phase A** establishes privacy of the distributed key-generation transcript, but does *not* claim privacy for the plaintext time-grid union steps beyond what is revealed by the final global alignment grid *T*_all_ and event-time grid *T*.
(ii) **Phase B** establishes computational indistinguishability of the encrypted aggregation transcript: honest-site ciphertext content is computationally hidden, subject to the public metadata exclusion stated in Theorem 21. The guarantee is assumption-based and relies on Assumption 2 for the distributed CKKS layer.
(iii) **Phase C** establishes simulation relative to the fused plaintext and released output: the decryption transcript of a corrupted committee member is simulatable from the aggregate plaintext table ${\{({n}_{i},{d}_{i})\}}_{i=1}^{| {T}_{{\rm{all}}}| }$, the signed metadata ${\mathsf{md}}$, the released curve ${\widehat{S}}_{{\rm{HE}}}(t)$, and the adversary’s local state, under the threshold-decryption assumptions stated in Theorem 22. Informally, ordinary sites receiving only ${\widehat{S}}_{{\rm{HE}}}(t)$ are exposed to less information than committee members, but this intuition is not formalized as a separate theorem.

Throughout this analysis we assume a *semi-honest* (honest-but-curious), *static* adversary: corruption sets are fixed before protocol execution begins, and corrupted parties follow the protocol faithfully while attempting to infer additional information from their observed transcripts. The adversarial view of a corrupted party includes its input, its full local random tape, and all received messages. Adaptive corruption and malicious deviations are outside the scope of this analysis.

The coordinator is modeled as a semi-honest, non-colluding aggregator: it performs homomorphic operations faithfully but may attempt to infer information from the ciphertexts and metadata it observes. Collusion between the coordinator and corrupted sites would enlarge the adversary’s view and is outside the present model.

Let ${{\mathcal{C}}}_{S}\subsetneq [K]$ denote the set of corrupted sites. Let ${\mathcal{R}}\subseteq [K]$ denote the set of parties authorized to participate in threshold decryption; in our implementation every site participates, so ${\mathcal{R}}=[K]$ and $R=| {\mathcal{R}}| =K$. Let ${{\mathcal{C}}}_{R}\subsetneq {\mathcal{R}}$ denote the set of corrupted decryptors. The protocol supports a general *θ*-of-*R* threshold; our implementation uses *θ* = *R*. Since ${{\mathcal{C}}}_{S}$ and ${{\mathcal{C}}}_{R}$ are indexed over different protocol roles, they are treated as independent corruption sets even when they overlap. When ${{\mathcal{C}}}_{S}\cap {{\mathcal{C}}}_{R}\ne {\rm{\varnothing }}$, the shared secret-key shares are carried as persistent adversary state across phases; the composition discussion below addresses this explicitly.

The simulators defined for the three phases may be composed into a single global simulator. The phases execute sequentially, and the inter-phase interfaces relevant to simulation consist only of public outputs: $({\mathsf{pk}},{T}_{{\rm{all}}})$ from Phase A and the ciphertext blocks from Phase B. Corrupted parties’ secret-key shares persist as auxiliary adversary state and are threaded unchanged through the composition; they do not alter the public inter-phase outputs to which the sequential composition argument applies.

Table 6 summarizes the security-specific notation used throughout the phase-wise proofs.

**Table 6:** Security-specific notation used in the phase-wise proofs (Theorems 20–22 and Corollary 23)

Symbol	Meaning
*Security parameters and models*	
*λ*	Security parameter.
PPT	Probabilistic polynomial-time (adversary, simulator, or algorithm).
${\mathcal{A}}$	PPT adversary.
${\mathsf{negl}}(\lambda )$	A negligible function of *λ*.
=_*d*_	Identical distribution between two random variables or ensembles.
$\mathop{\approx }\limits^{c}$	Computational indistinguishability (advantage bounded by ${\mathsf{negl}}(\lambda )$).
Transcript	The full set of messages and local state observed by a party (or adversary) during protocol execution, formalized as ${{\mathsf{View}}}_{{\mathcal{A}}}^{(\cdot )}$.
*Protocol and corruption*	
*Π*	The protocol *Π*_KM−Fed−CKKS_ (shorthand used in proofs).
${{\mathcal{C}}}_{S}\subsetneq [K]$	Static set of corrupted sites (strict subset); fixed before execution begins.
${{\mathcal{C}}}_{R}\subsetneq {\mathcal{R}}$	Static set of corrupted decryptors (strict subset); fixed before execution begins.
*ρ*_*k*_	Local key-generation randomness of site *k* (revealed if $k\in {{\mathcal{C}}}_{S}$), part of its full random tape.
${\rho }_{k}^{{\rm{enc}}}$	Encryption randomness used by site *k* in Phase B (revealed if $k\in {{\mathcal{C}}}_{S}$), part of its full random tape.
*Adversarial views*	
${{\mathsf{View}}}_{{\mathcal{A}}}^{(A)}(\Pi )$	Phase A view: ${\mathsf{Params}}$, corrupted DKG state ${\{{{\mathsf{sk}}}_{k},{\rho }_{k}\}}_{k\in {{\mathcal{C}}}_{S}}$, joint ${\mathsf{pk}}$, complete DKG transcript, global alignment grid *T*_all_, event-time grid *T*.
${{\mathsf{View}}}_{{\mathcal{A}}}^{(B)}(\Pi )$	Phase B view: public setup; corrupted sites’ inputs ${\{{({d}_{i}^{(k)},{n}_{i}^{(k)})}_{i=1}^{L}\}}_{k\in {{\mathcal{C}}}_{S}}$, secret-key shares, and encryption randomness; all uploaded ciphertexts ${\{{{\mathsf{ct}}}_{\iota }^{(k)}\}}_{k\in [K],\iota \in {\mathcal{I}}}$ and aggregated ciphertexts ${\{{{\mathsf{ct}}}_{\Sigma ,\iota }\}}_{\iota \in {\mathcal{I}}}$.
${{\mathsf{View}}}_{{\mathcal{A}}}^{(C)}(\Pi )$	Phase C view: aggregated ciphertexts ${\{{{\mathsf{ct}}}_{\Sigma ,\iota }\}}_{\iota \in {\mathcal{I}}}$; corrupted secret-key shares ${\{{{\mathsf{sk}}}_{r}\}}_{r\in {{\mathcal{C}}}_{R}}$ and partial decryption shares ${\{{\Delta }_{\iota }^{(r)}\}}_{r\in {{\mathcal{C}}}_{R},\iota \in {\mathcal{I}}}$; signed metadata ${\mathsf{md}}$; aggregate plaintext table $P:={\{({n}_{i},{d}_{i})\}}_{i=1}^{L}$; released curve ${\widehat{S}}_{{\rm{HE}}}(t)$.
*Simulators*	
${{\mathcal{S}}}_{A}$	PPT simulator for Phase A; input: $({\mathsf{Params}},\,{\{{{\mathsf{sk}}}_{k},{\rho }_{k}\}}_{k\in {{\mathcal{C}}}_{S}},\,{T}_{{\rm{all}}},\,T)$.
${{\mathcal{S}}}_{B}$	PPT simulator for Phase B; input: $({\mathsf{Params}},\,{\{{({d}_{i}^{(k)},{n}_{i}^{(k)})}_{i=1}^{L},{{\mathsf{sk}}}_{k},{\rho }_{k}^{{\rm{enc}}}\}}_{k\in {{\mathcal{C}}}_{S}},\,{\mathsf{pk}})$.
${{\mathcal{S}}}_{C}$	PPT simulator for Phase C; input: $({\{{{\mathsf{sk}}}_{r}\}}_{r\in {{\mathcal{C}}}_{R}},\,{\{{{\mathsf{ct}}}_{\Sigma ,\iota }\}}_{\iota \in {\mathcal{I}}},\,{\mathsf{md}},\,P,\,{\widehat{S}}_{{\rm{HE}}}(t))$.
${{\mathcal{S}}}_{{\rm{share}}}$	PPT share-privacy simulator guaranteed by Assumption (ii) of Theorem 22.
*Phase B proof objects*	
$| {\mathcal{I}}|$	Total number of ciphertext blocks per site under the chosen layout; $| {\mathcal{I}}| ={M}_{{\rm{int}}}$ for interleaving and $| {\mathcal{I}}| ={M}_{{\rm{sep}}}$ for separate packing. Public and identical across sites.
*N*	Total number of honest-site ciphertext blocks in the hybrid argument; $N=(K-| {{\mathcal{C}}}_{S}| )\cdot | {\mathcal{I}}|$.
*H*^(*ℓ*)^	Hybrid distribution in the Phase B proof: first *ℓ* honest-site ciphertext blocks replaced by encryptions of **0**_*B*_ (the zero vector in ${{\mathbb{R}}}^{B}$), remainder real; *H*^(0)^ is the real execution, *H*^(*N*)^ is the simulator output.
${\mathsf{Real}}$, ${\mathsf{Real}}{\prime}$	Real execution (${\mathsf{Real}}$) and a re-randomized equivalent execution (${\mathsf{Real}}{\prime}$) used in the initial hybrid step of the Phase B proof.
*Phase C proof objects*	
${\mathsf{md}}$	Signed metadata blob: coordinator-authenticated statement attesting the cohort identifier, committed alignment grid *T*_all_, and ciphertext block identifiers ${\{{{\mathsf{ct}}}_{\Sigma ,\iota }\}}_{\iota \in {\mathcal{I}}}$.
*P*	Fused plaintext aggregate table ${\{({n}_{i},{d}_{i})\}}_{i=1}^{L}$ obtained after threshold decryption; used as input in the share-privacy assumption of Theorem 22.
*Assumptions*	
Assumption 2	Threshold-aware auxiliary-input IND-CPA for the distributed CKKS layer: ciphertext indistinguishability holds even when the adversary holds a strict subset of secret-key shares and polynomially many auxiliary ciphertexts under the joint public key.

Symbols shared with the main protocol are defined in Table 4; this table contains only notation that is specific to or has a refined meaning within the security analysis.

Phase A consists of two substeps: (a) distributed key generation, which produces the joint public key ${\mathsf{pk}}$ and local secret-key shares $\{{{\mathsf{sk}}}_{k}\}$; and (b) construction of the global alignment grid

$${T}_{{\rm{all}}}={\rm{Sort}}\left(\mathop{\bigcup }\limits_{k=1}^{K}{T}_{k}^{{\rm{all}}}\right).$$

The event-time subset *T* = {*t* ∈ *T*_all_: ∃ an even tat *t*} is a theoretical object; it is not constructed explicitly in the implementation. The first substep is data-independent; the second intentionally reveals the union of local observed-time sets.

Let ${{\mathsf{View}}}_{{\mathcal{A}}}^{(A)}(\Pi )$ denote the Phase A view of a PPT adversary ${\mathcal{A}}$ corrupting ${{\mathcal{C}}}_{S}\subsetneq [K]$. This view consists of:

- the public CKKS parameters ${\mathsf{Params}}$ and all public setup values;
- the corrupted sites’ secret-key shares ${\{{{\mathsf{sk}}}_{k}\}}_{k\in {{\mathcal{C}}}_{S}}$ and their local key-generation randomness ${\{{\rho }_{k}\}}_{k\in {{\mathcal{C}}}_{S}}$;
- the joint public key ${\mathsf{pk}}$ and the complete DKG transcript; and
- the final global alignment grid *T*_all_ and event-time grid *T*.

In the implementation used here, only *T*_all_ is explicitly disclosed; the event-time grid *T* is included in the adversarial view for theoretical generality, since *T* ⊆ *T*_all_ is derivable from the data. The theorem therefore provides a conservative (overgenerous in leakage) guarantee.

#### Theorem 20

(Phase A setup privacy) Assume the distributed key-generation mechanism used in *Π* is correctly specified and data-independent: for any fixed corrupted-party state ${\{{{\mathsf{sk}}}_{k},{\rho }_{k}\}}_{k\in {{\mathcal{C}}}_{S}}$, the joint distribution of the DKG transcript and joint public key depends only on the parties’ key-generation randomness and secret-key material, not on any site’s plaintext dataset. Then there exists a PPT simulator ${{\mathcal{S}}}_{A}$ such that

$${{\mathcal{S}}}_{A}\left({\mathsf{Params}},\,{\{{{\mathsf{sk}}}_{k},{\rho }_{k}\}}_{k\in {{\mathcal{C}}}_{S}},\,{T}_{{\rm{all}}},\,T\right)\,{=}_{d}\,{{\mathsf{View}}}_{{\mathcal{A}}}^{(A)}(\Pi ),$$

where = _*d*_ denotes identical distribution.

#### Proof.

The simulator ${{\mathcal{S}}}_{A}$ operates as follows. For each honest party $k\notin {{\mathcal{C}}}_{S}$, it samples fresh key-generation randomness ${\widetilde{\rho }}_{k}$ independently from the same distribution specified by the DKG protocol. It then runs the DKG protocol using the real corrupted-party inputs ${\{{{\mathsf{sk}}}_{k},{\rho }_{k}\}}_{k\in {{\mathcal{C}}}_{S}}$ and the freshly sampled ${\{{\widetilde{\rho }}_{k}\}}_{k\notin {{\mathcal{C}}}_{S}}$, producing a simulated DKG transcript and joint public key $\hat{{\mathsf{pk}}}$. It appends *T*_all_ and *T*.

By the data-independence assumption, the joint distribution of $({\rm{DKGtranscript}},{\mathsf{pk}})$ depends only on key-generation randomness and secret-key material. In the real execution, the honest parties’ randomness ${\{{\rho }_{k}\}}_{k\notin {{\mathcal{C}}}_{S}}$ is drawn independently from the prescribed distribution; the simulator draws ${\{{\widetilde{\rho }}_{k}\}}_{k\notin {{\mathcal{C}}}_{S}}$ from the identical distribution. Since the corrupted-party state is identical in both cases, and the honest randomness has the same distribution, the joint distribution of the DKG transcript, $\hat{{\mathsf{pk}}},\,{T}_{{\rm{all}}}$, and *T* in the simulation is identical to that of the DKG transcript, ${\mathsf{pk}},\,{T}_{{\rm{all}}}$, and *T* in a real execution.

Theorem 20 does not claim privacy for the individual sets ${T}_{k}^{{\rm{all}}}$ beyond what *T*_all_ reveals. In the present implementation the union is computed in plaintext; timepoints are treated as non-sensitive metadata. If a stricter deployment requires hiding ${T}_{k}^{{\rm{all}}}$, the union step must be replaced by a private-set-union protocol analyzed separately.

Each site *k* computes local Kaplan–Meier count pairs ${({d}_{i}^{(k)},{n}_{i}^{(k)})}_{i=1}^{| {T}_{{\rm{all}}}| }$ over the shared alignment grid *T*_all_, packs them into CKKS plaintext vectors, encrypts them under ${\mathsf{pk}}$, and uploads the resulting ciphertext blocks. Sites with no event at a given grid point contribute ${d}_{i}^{(k)}=0$ for that position. The coordinator performs only homomorphic addition on received ciphertexts.

Let *B* denote the number of plaintext slots in a single CKKS ciphertext, a public parameter (with *B* = *n*/2 for CKKS). Each site *k* packs its count pairs over the alignment grid *T*_all_ into CKKS plaintext vectors using a fixed, publicly known packing algorithm. With packed grid length *L* = ∣*T*_all_∣, the public ciphertext-index set ${\mathcal{I}}$ has $| {\mathcal{I}}| ={M}_{{\rm{int}}}=\lceil 2L/B\rceil$ for interleaving and $| {\mathcal{I}}| ={M}_{{\rm{sep}}}=2\lceil L/B\rceil$ for separate packing. We write ${{\bf{z}}}_{\iota }^{(k)}\in {{\mathbb{R}}}^{B}$ for the packed vector of site *k* at index $\iota \in {\mathcal{I}}$. Since *B*, *L*, and ${\mathcal{I}}$ are public, all ciphertext block dimensions are derivable without accessing any site’s private data. Because every site packs counts over the same global alignment grid *T*_all_, the index set ${\mathcal{I}}$ is identical for all sites. The proof below is stated for a generic ι; for interleaving each ι is a block index, while for separate packing each ι = (*j*, *s*) pairs a block index *j* ∈ [*M*_0_] with a stream label *s* ∈ {*d*, *n*}.

Standard IND-CPA for public-key encryption is stated for adversaries that see the public key but not any secret-key material. In our distributed setting, a Phase B adversary may additionally hold a strict subset of secret-key shares produced by the DKG. To make the security claim precise, we isolate the following assumption for the distributed CKKS layer used by *Π*_KM_ − Fed − CKKS.

#### Assumption 2

(Threshold-aware auxiliary-input IND-CPA) Let ${\mathsf{pk}}$ be a joint public key produced by the distributed key-generation procedure, and let ${\{{{\mathsf{sk}}}_{k}\}}_{k\in {{\mathcal{C}}}_{S}}$ be any strict subset of secret-key shares with $| {{\mathcal{C}}}_{S}| < \theta$. Then ciphertext privacy remains computationally secure against any PPT distinguisher given:

(i) the public key ${\mathsf{pk}}$,
(ii) the corrupted shares ${\{{{\mathsf{sk}}}_{k}\}}_{k\in {{\mathcal{C}}}_{S}}$, and
(iii) polynomially many auxiliary ciphertexts under ${\mathsf{pk}}$,

in the following sense: for any equal-dimension plaintexts **m**_0_, **m**_1_, the distributions

$$({\mathsf{pk}},{\{{{\mathsf{sk}}}_{k}\}}_{k\in {{\mathcal{C}}}_{S}},{\mathsf{aux}},{E}_{{\mathsf{pk}}}({{\bf{m}}}_{0}))\,{\rm{and}}\,({\mathsf{pk}},{\{{{\mathsf{sk}}}_{k}\}}_{k\in {{\mathcal{C}}}_{S}},{\mathsf{aux}},{E}_{{\mathsf{pk}}}({{\bf{m}}}_{1}))$$

are computationally indistinguishable for every PPT-computable auxiliary input ${\mathsf{aux}}$ consisting of polynomially many ciphertexts under ${\mathsf{pk}}$.

*Remark*. Assumption 2 is stronger than standard IND-CPA because the distinguisher is additionally given a strict subset of secret-key shares and auxiliary ciphertexts. We therefore state it explicitly as an assumption on the distributed CKKS layer used by our protocol; establishing it formally for a specific threshold CKKS instantiation is outside the scope of this work.

Let ${{\mathsf{View}}}_{{\mathcal{A}}}^{(B)}(\Pi )$ denote the pre-decryption Phase B view of a PPT adversary ${\mathcal{A}}$ corrupting ${{\mathcal{C}}}_{S}\subsetneq [K]$. This view consists of:

- the public parameters ${\mathsf{Params}}$, the public key ${\mathsf{pk}}$, and public setup values from Phase A;
- the corrupted sites’ inputs ${\left\{{({d}_{i}^{(k)},{n}_{i}^{(k)})}_{i=1}^{| {T}_{\mathrm{all}}| }\right\}}_{k\in {{\mathcal{C}}}_{S}}$, secret-key shares ${\{{{\mathsf{sk}}}_{k}\}}_{k\in {{\mathcal{C}}}_{S}}$, and encryption randomness ${\{{\rho }_{k}^{{\rm{enc}}}\}}_{k\in {{\mathcal{C}}}_{S}}$;
- ciphertext uploads ${\{{{\mathsf{ct}}}_{j}^{(k)}\}}_{k\in [K],j\in [M]}$ from all sites; and
- aggregated ciphertexts ${\{{{\mathsf{ct}}}_{\Sigma ,j}\}}_{j=1}^{M}$ produced by the coordinator.

The coordinator is treated as semi-honest: it performs homomorphic aggregation faithfully. The corrupted sites’ encryption randomness ${\{{\rho }_{k}^{{\rm{enc}}}\}}_{k\in {{\mathcal{C}}}_{S}}$ is included in the view because the adversarial model includes each corrupted party’s full local random tape.

#### Theorem 21

(Transcript privacy of Phase B encrypted aggregation) Assume Assumption 2. Then there exists a PPT simulator ${{\mathcal{S}}}_{B}$ such that

$${{\mathcal{S}}}_{B}\,\left({\mathsf{Params}},\,{\{{({d}_{i}^{(k)},{n}_{i}^{(k)})}_{i=1}^{| {T}_{{\rm{all}}}| },{{\mathsf{sk}}}_{k},{\rho }_{k}^{{\rm{enc}}}\}}_{k\in {{\mathcal{C}}}_{S}},\,{\mathsf{pk}}\right)\,\mathop{\approx }\limits^{c}\,{{\mathsf{View}}}_{{\mathcal{A}}}^{(B)}(\Pi ).$$

That is, the Phase B encrypted aggregation transcript is computationally indistinguishable from a simulation that does not use the honest sites’ local Kaplan–Meier count vectors. This guarantee covers ciphertext *content* privacy only; public metadata such as the common block count *M*, ciphertext sizes, and upload timing is excluded.

#### Proof.

We construct ${{\mathcal{S}}}_{B}$ and prove indistinguishability by a standard sequence-of-games argument.

Given ${\mathsf{Params}}$, the corrupted sites’ inputs, corrupted secret-key shares, corrupted encryption randomness, and ${\mathsf{pk}}$, the simulator ${{\mathcal{S}}}_{B}$ proceeds as follows. For notational simplicity, ${\rho }_{k}^{{\rm{enc}}}$ denotes the full encryption random tape of corrupted site *k*, whose per-block components are written as ${\rho }_{k,j}^{{\rm{enc}}}$ when individual ciphertext blocks are referenced.

(i) It sets $$\hat{{\mathsf{pk}}}:={\mathsf{pk}}$$.

(ii) For each corrupted site $k\in {{\mathcal{C}}}_{S}$, it computes the packed plaintext vectors ${{\bf{z}}}_{j}^{(k)}$ from the given corrupted inputs and encrypts them using the given randomness:

$${\hat{{\mathsf{ct}}}}_{j}^{(k)}:={E}_{\hat{{\mathsf{pk}}}}({{\bf{z}}}_{j}^{(k)};\,{\rho }_{k,j}^{{\rm{enc}}}),\,j=1,\ldots ,M.$$

Hence the corrupted sites’ ciphertexts are reproduced exactly.

(iii) Since every site packs over the same public alignment grid *T*_all_, each site uploads exactly *M* ciphertext blocks. For each honest site $k\notin {{\mathcal{C}}}_{S}$ and each *j* = 1, …, *M*, the simulator outputs

$${\widetilde{{\mathsf{ct}}}}_{j}^{(k)}\leftarrow {E}_{\tilde{{\mathsf{pk}}}}({{\bf{0}}}_{B}),$$

using fresh randomness.

(iv) For each block index *j*, it computes the aggregate ciphertext by CKKS homomorphic addition:

$${\overline{{\mathsf{ct}}}}_{\Sigma ,j}=\mathop{\bigoplus }\limits_{k=1}^{K}{\overline{{\mathsf{ct}}}}_{j}^{(k)}.$$

Let

$$N=(K-| {{\mathcal{C}}}_{S}| )\cdot M$$

be the total number of honest-site ciphertext blocks. Fix an arbitrary but deterministic ordering of these honest blocks, and define hybrids

$${H}^{(0)},{H}^{(1)},\ldots ,{H}^{(N)},$$

where:

- *H*^(0)^ is the real Phase B transcript;
- in *H*^(*ℓ*)^, the first *ℓ* honest ciphertext blocks in the fixed ordering are replaced by encryptions of **0**_*B*_, while all remaining honest blocks are real encryptions; and
- *H*^(*N*)^ is exactly the simulator output.

The corrupted sites’ inputs, secret-key shares, encryption randomness, and ciphertexts are identical in every hybrid. The only changes occur in the honest ciphertext blocks.

Now consider adjacent hybrids *H*^(*ℓ*−1)^ and *H*^(*ℓ*)^. They differ in exactly one honest ciphertext block, say block (*k*, *j*). In *H*^(*ℓ*−1)^ that block is

$${E}_{{\mathsf{pk}}}\left({{\bf{z}}}_{j}^{(k)}\right),$$

whereas in *H*^(*ℓ*)^ it is

$${E}_{{\mathsf{pk}}}({{\bf{0}}}_{B}).$$

All other ciphertexts, including all corrupted-site ciphertexts and the other honest-site ciphertexts, are unchanged. Therefore they may be treated as auxiliary input in Assumption 2, together with the corrupted secret-key shares.

If some PPT distinguisher separated *H*^(*ℓ*−1)^ from *H*^(*ℓ*)^ with non-negligible advantage, then this would violate Assumption 2 for the plaintext pair

$$\left({{\bf{z}}}_{j}^{(k)},\,{{\bf{0}}}_{B}\right),$$

with the unchanged ciphertext blocks and corrupted shares supplied as auxiliary input. Hence

$${H}^{(\ell -1)}\mathop{\approx }\limits^{c}{H}^{(\ell )}\,{\rm{forevery}}\ell =1,\ldots ,N.$$

Because *N* = poly(*λ*), a standard telescoping hybrid argument gives

$${H}^{(0)}\mathop{\approx }\limits^{c}{H}^{(N)}.$$

Finally, the aggregate ciphertexts are deterministic efficient functions of the uploaded ciphertext blocks. Computational indistinguishability is preserved under such post-processing, so the aggregate ciphertext components remain computationally indistinguishable as well.

Since *H*^(*N*)^ is exactly the transcript output by ${{\mathcal{S}}}_{B}$, we obtain

$${{\mathcal{S}}}_{B}\left({\mathsf{Params}},\,{\{{({d}_{i}^{(k)},{n}_{i}^{(k)})}_{i=1}^{| {T}_{{\rm{all}}}| },{{\mathsf{sk}}}_{k},{\rho }_{k}^{{\rm{enc}}}\}}_{k\in {{\mathcal{C}}}_{S}},\,{\mathsf{pk}}\right)\mathop{\approx }\limits^{c}{{\mathsf{View}}}_{{\mathcal{A}}}^{(B)}(\Pi ),$$

as claimed.

The above argument covers ciphertext *content* privacy. The block count *M*, upload timing, and ciphertext sizes are observable metadata. Because all sites pack over the same global alignment grid *T*_all_, the block count *M* is identical across sites and reveals only ∣*T*_all_∣, which is already public from Phase A. Upload timing may reveal participation ordering and is outside the cryptographic guarantee; it should be addressed at the network layer if needed.

Phase C releases a public result, so the security goal is not full hiding but simulation relative to the information legitimately revealed.

The following Phase C claim is output-relative: it simulates the view of a corrupted decryptor coalition given the aggregate plaintext table recovered inside the decryption committee and the released curve ${\widehat{S}}_{{\rm{HE}}}(t)$. It does *not* assert that a subthreshold coalition can recover the aggregate table on its own. The adversary is modeled relative to the internal decryption outcome available within the committee execution. Informally, ordinary sites receiving only ${\widehat{S}}_{{\rm{HE}}}(t)$ are exposed to less information than committee members, but this is not formalized as a separate theorem. The theorem does not claim security against adaptive decryption queries, which are operationally excluded by policy controls in section “Threat model, threshold, and key management”. The simulated leakage for Phase C is ${{\mathcal{L}}}_{C}=({\mathsf{md}},P,{\widehat{S}}_{{\rm{HE}}}(t))$, together with the corrupted parties’ local keys and local randomness.

The decryption request is accompanied by a signed metadata blob ${\mathsf{md}}$: a coordinator-authenticated statement attesting the cohort identifier, the committed alignment grid *T*_all_, and the ciphertext block identifiers ${\{{{\mathsf{ct}}}_{\Sigma ,\iota }\}}_{\iota \in {\mathcal{I}}}$. The signature is produced under a signing key provisioned separately from the CKKS setup, for example via out-of-band PKI. The Phase C adversarial view includes ${\mathsf{md}}$ directly; the simulator receives ${\mathsf{md}}$ as explicit input and copies it verbatim, requiring no signing capability.

Let ${{\mathsf{View}}}_{{\mathcal{A}}}^{(C)}(\Pi )$ denote the Phase C view of a PPT adversary corrupting ${{\mathcal{C}}}_{R}\subsetneq {\mathcal{R}}$. This view consists of:

- the aggregated ciphertexts ${\{{{\mathsf{ct}}}_{\Sigma ,\iota }\}}_{\iota \in {\mathcal{I}}}$;
- the corrupted decryptors’ secret-key shares ${\{{{\mathsf{sk}}}_{r}\}}_{r\in {{\mathcal{C}}}_{R}}$;
- the corrupted decryptors’ partial decryption shares ${\{{\Delta }_{\iota }^{(r)}\}}_{r\in {{\mathcal{C}}}_{R},\iota \in {\mathcal{I}}}$, computed deterministically from $({{\mathsf{sk}}}_{r},{{\mathsf{ct}}}_{\Sigma ,\iota })$ by the partial decryption algorithm;
- the signed metadata blob ${\mathsf{md}}$;
- the aggregate plaintext table $P:={\{({n}_{i},{d}_{i})\}}_{i=1}^{L}$ produced by threshold decryption; and
- the released survival curve ${\widehat{S}}_{{\rm{HE}}}(t)$, computed deterministically from *P* via the Kaplan–Meier formula.

The aggregate table is the direct plaintext output of the decryption step and is modeled relative to the internal decryption outcome available within the committee execution. In deployments where only ${\widehat{S}}_{{\rm{HE}}}(t)$ is externally published, the table remains internal to the decryptor committee. The Kaplan–Meier estimator depends only on the event-time subset *T* ⊆ *T*_all_, since *d*_*i*_ = 0 for time points without events.

#### Theorem 22

(Phase C privacy relative to the fused plaintext and released output) Assume the threshold decryption mechanism satisfies:

(i) *Threshold confidentiality:* no PPT adversary holding fewer than *θ* secret-key shares can recover the plaintext of a well-formed ciphertext with non-negligible advantage in *λ*.

(ii) *Share privacy:* for any fixed ciphertext collection ${\{{{\mathsf{ct}}}_{\Sigma ,\iota }\}}_{\iota \in {\mathcal{I}}}$, any strict subset ${{\mathcal{C}}}_{R}$ with $| {{\mathcal{C}}}_{R}| < \theta$, and the correct fused plaintext table $P={\{({n}_{i},{d}_{i})\}}_{i=1}^{L}$, there exists a PPT simulator ${{\mathcal{S}}}_{{\rm{share}}}$ such that the simulated honest partial shares are computationally indistinguishable from the real ones:

$${{\mathcal{S}}}_{\mathrm{share}}\left({\{{{\mathsf{sk}}}_{r}\}}_{r\in {{\mathcal{C}}}_{R}},\,{\{{{\mathsf{ct}}}_{\Sigma ,\iota }\}}_{\iota \in {\mathcal{I}}},\,P\right)\,\mathop{\approx }\limits^{c}\,{\left\{{\Delta }_{\iota }^{(r)}\right\}}_{r\notin {{\mathcal{C}}}_{R},\iota \in {\mathcal{I}}}.$$

(iii) *Fusion correctness:* a valid threshold set of partial shares combined by the fusion algorithm yields the correct plaintext.

(iv) *Deterministic partial decryption:* the partial decryption algorithm is deterministic given $({{\mathsf{sk}}}_{r},{{\mathsf{ct}}}_{\Sigma ,\iota })$.

Assumptions (ii)–(iv) are used directly in the simulation proof below. Assumption (i) is not used in the indistinguishability argument itself, but justifies interpreting the simulator input as an upper bound on the information available to any subthreshold coalition.

Then there exists a PPT simulator ${{\mathcal{S}}}_{C}$ such that

$${{\mathcal{S}}}_{C}\,\left({\{{{\mathsf{sk}}}_{r}\}}_{r\in {{\mathcal{C}}}_{R}},\,{\{{{\mathsf{ct}}}_{\Sigma ,\iota }\}}_{\iota \in {\mathcal{I}}},\,{\mathsf{md}},\,P,\,{\widehat{S}}_{{\rm{HE}}}(t)\right)\,\mathop{\approx }\limits^{c}\,{{\mathsf{View}}}_{{\mathcal{A}}}^{(C)}(\Pi ).$$

In the all-of-*R* implementation, this holds for every strict subset ${{\mathcal{C}}}_{R}\subsetneq {\mathcal{R}}$, since no incomplete coalition can complete decryption independently.

*Interpretation*. Theorem 22 is a protocol-level reduction to the assumed security of the threshold decryption mechanism, not a derivation from RLWE alone.

#### Proof.

Construct ${{\mathcal{S}}}_{C}$ as follows, given

$$({\{{{\mathsf{sk}}}_{r}\}}_{r\in {{\mathcal{C}}}_{R}},\,{\{{{\mathsf{ct}}}_{\Sigma ,\iota }\}}_{\iota \in {\mathcal{I}}},\,{\mathsf{md}},\,P,\,{\widehat{S}}_{{\rm{HE}}}(t)).$$

(a) *Metadata*. Copy ${\mathsf{md}}$ verbatim; no signing key is needed. (b) *Corrupted partial shares*. For each $r\in {{\mathcal{C}}}_{R}$ and each $\iota \in {\mathcal{I}}$, compute ${\Delta }_{\iota }^{(r)}$ using ${{\mathsf{sk}}}_{r}$ and ${{\mathsf{ct}}}_{\Sigma ,\iota }$ exactly as in the real protocol. By assumption (iv), this matches the real partial shares with probability 1. (c) *Honest partial shares*. Invoke the share-privacy simulator ${{\mathcal{S}}}_{{\rm{share}}}$ from assumption (ii) with inputs

$$({\{{{\mathsf{sk}}}_{r}\}}_{r\in {{\mathcal{C}}}_{R}},\,{\{{{\mathsf{ct}}}_{\Sigma ,\iota }\}}_{\iota \in {\mathcal{I}}},\,P)$$

to obtain simulated partial shares ${\{{\widetilde{\Delta }}_{\iota }^{(r)}\}}_{r\notin {{\mathcal{C}}}_{R},\iota \in {\mathcal{I}}}$. (d) *Public outputs*. Append *P* and ${\widehat{S}}_{{\rm{HE}}}(t)$.

We compare the simulated transcript ${{\mathcal{S}}}_{C}(\cdot )$ with ${{\mathsf{View}}}_{{\mathcal{A}}}^{(C)}(\Pi )$ component by component.

• *Metadata:* copied exactly, hence identically distributed.

• *Corrupted partial shares:* computed identically to the real protocol by assumption (iv), hence identically distributed.

• *Honest partial shares:* computationally indistinguishable from the real shares by share privacy, assumption (ii).

• *Aggregate table and released curve:* given as simulator input; in the real protocol these are computed from the ciphertexts via threshold decryption, which by fusion correctness, assumption (iii), yields the same aggregate table. The curve ${\widehat{S}}_{{\rm{HE}}}(t)$ is a deterministic function of that table. Hence these components are identically distributed.

Any PPT distinguisher between the simulation and the real Phase C view would therefore distinguish simulated honest shares from real honest shares, violating share privacy, assumption (ii). Hence

$${{\mathcal{S}}}_{C}\,\left({\{{{\mathsf{sk}}}_{r}\}}_{r\in {{\mathcal{C}}}_{R}},\,{\{{{\mathsf{ct}}}_{\Sigma ,\iota }\}}_{\iota \in {\mathcal{I}}},\,{\mathsf{md}},\,P,\,{\widehat{S}}_{{\rm{HE}}}(t)\right)\mathop{\approx }\limits^{c}{{\mathsf{View}}}_{{\mathcal{A}}}^{(C)}(\Pi ).$$

The theorem is strongest when fusion and Kaplan–Meier evaluation are performed inside the decryptor committee and only ${\widehat{S}}_{{\rm{HE}}}(t)$ is released externally. If the combiner is co-located with the coordinator, the coordinator transiently observes *P*; the theorem still characterizes the committee-member transcript while ordinary sites observe only ${\widehat{S}}_{{\rm{HE}}}(t)$. The coordinator’s transient access to the aggregate table is a potential disclosure surface and should be mitigated operationally, for example through hardened execution environments, audit logging, and access controls.

#### Corollary 23

(End-to-end privacy of *Π*_KM−Fed−CKKS_: phase-level leakage summary) Under the assumptions of Theorems 20, 21, and 22, the per-phase privacy guarantees can be jointly interpreted as follows:

(i) **Phase A:** the setup transcript is identically distributed to a simulation that uses no honest-site plaintext data, beyond the intentionally revealed alignment grid *T*_all_ and event-time grid *T*.

(ii) **Phase B:** the encrypted aggregation transcript is computationally indistinguishable from a simulation that uses no honest-site Kaplan–Meier count vectors, beyond publicly observable metadata such as the ciphertext-index set ${\mathcal{I}}$, ciphertext sizes, and upload timing.

(iii) **Phase C:** the decryption transcript of a corrupted committee member is computationally indistinguishable from a simulation that uses only the aggregate plaintext table *P*, the signed metadata, and the released curve ${\widehat{S}}_{{\rm{HE}}}(t)$.

#### Proof.

Items (i)–(iii) are the direct phase-wise consequences of Theorems 20, 21, and 22. The sequential composition discussion above justifies viewing them jointly as an end-to-end phase-level leakage summary, because the following conditions hold: (1) the phases execute sequentially; (2) each phase’s public output, namely $({\mathsf{pk}},{T}_{{\rm{all}}},T)$ from Phase A and the ciphertext blocks from Phase B, is the sole inter-phase communication channel; (3) corrupted parties’ secret-key shares persist as auxiliary adversary state threaded unchanged through the composition and do not affect the public inter-phase outputs; and (4) all individual-phase simulators run in PPT.

When ${{\mathcal{C}}}_{S}\cap {{\mathcal{C}}}_{R}\ne {\rm{\varnothing }}$, the shared secret-key shares appear in the adversary’s input for both Phase B and Phase C. This does not invalidate the per-phase claims: the Phase B simulator takes these shares as explicit input, and the Phase C simulator independently takes its corrupted shares as explicit input. Crucially, Theorem 22 holds for *any* fixed input ciphertext collection ${\{{{\mathsf{ct}}}_{\Sigma ,\iota }\}}_{\iota \in {\mathcal{I}}}$, so the Phase C guarantee remains valid even when the aggregated ciphertexts are simulated outputs from Phase B rather than the real aggregates.

The above analysis establishes simulation-based privacy under a semi-honest, static adversarial model. Extending to the malicious setting would require additional mechanisms such as zero-knowledge proofs of correct encryption or range proofs compatible with CKKS approximate arithmetic, ciphertext validity checks, and verifiable threshold decryption, and is left for future work.

The guarantees are transcript-based and do not eliminate all leakage channels. Upload timing and network-layer metadata may reveal participation ordering and should be mitigated at the transport layer if needed. The plaintext time-grid union reveals the union of local timepoint sets, treated as non-sensitive in the present deployment but potentially requiring a private-set-union protocol in stricter settings. The analysis also excludes coordinator-site collusion; such collusion would enlarge the adversarial view beyond the present model, combining the coordinator’s observed ciphertexts and metadata with corrupted sites’ secret-key shares and plaintext inputs, and would require a separate analysis. Our Phase B claim, therefore, relies on Assumption 2 for the distributed CKKS layer, rather than on a standalone reduction proved in this work.

The Phase C guarantee is relative to the fused plaintext and released output, and depends on trusted execution of the threshold decryption workflow. If intermediate aggregate plaintexts are exposed outside the decryptor committee, for example due to co-location of the combiner with the coordinator, then additional trust assumptions or secure execution environments are required. These considerations highlight that the protocol provides strong cryptographic protection for local Kaplan–Meier contributions, while leaving certain system-level leakage paths and deployment choices to complementary safeguards.

### IID vs. non-IID (practical invariance)

The cohort is fixed centrally, preprocessing is harmonized (units, censoring/ties), and all sites align to the common global alignment grid *T*_all_ in R1, from which the event-time grid *T* ⊆ *T*_all_ is derived. Under these conditions, per-time-point counts add exactly on the event-time grid (${d}_{i}={\sum }_{k}{d}_{i}^{(k)},\,{n}_{i}={\sum }_{k}{n}_{i}^{(k)}$ for *t*_*i*_ ∈ *T*), so the federated KM is *partition-invariant* (Theorem 5): IID vs. non-IID does not change ${\widehat{S}}_{{\rm{HE}}}(t)$. Differences arise only if harmonization or cohort assumptions are violated (section “Discussion”).

### Design choices and guarantees

The number of ciphertext blocks *M* grows stepwise with the length of the grid used for encrypted alignment and depends on the packing layout. In the current implementation, packing is performed over *T*_all_, so

$${M}_{\mathrm{int}}=\left\lceil \frac{2|{T}_{\mathrm{all}}|}{B}\right\rceil \,\mathrm{and}\,{M}_{\mathrm{sep}}=2\left\lceil \frac{|{T}_{\mathrm{all}}|}{B}\right\rceil ,$$

which determine per-site uplink and server additions. If packing were instead performed over the event-time subset *T*, the same formulas would hold with ∣*T*∣ in place of ∣*T*_all_∣. Let *R* denote the committee size (number of decryptors), where *R* ≤ *K*; experiments use *R* = *K* by default, while fixed-*R* variants are included for communication studies. The decryption threshold *θ* determines how many decryptors must participate to recover plaintext; we set *θ* = *R* by default (strict threshold).

Our protocol provides these guarantees under the stated threat model: no party learns other sites’ plaintext contributions. The coordinator sees only ciphertexts in Phase B. In Phase C, if fusion is co-located with the coordinator, it may transiently observe the aggregated plaintext count vectors (*n*_*i*_, *d*_*i*_) over the aligned grid required for Kaplan–Meier evaluation; however, individual site contributions are never decrypted separately, and only the final survival curve ${\widehat{S}}_{{\rm{HE}}}(t)$ is released to sites, eliminating the subtraction-based residualization that is present when (*d*_*i*_, *n*_*i*_) are disclosed. The computational complexity of the protocol is characterized as follows: Phase A builds *T*_all_ in $O({\sum }_{k}| {T}_{k}^{{\rm{all}}}| \log {\sum }_{k}| {T}_{k}^{{\rm{all}}}| )$ time; Phase B performs *O*(*MK*) homomorphic additions with linear-in-*K* uplink; and Phase C performs *O*(*RM*) share generations, *O*(*M*) fusions, and *O*(∣*T*_all_∣) decoding work, followed by an *O*(∣*T*∣) Kaplan–Meier pass on the event-time grid.

With the same event-time grid *T* and add-only aggregation, ${\widehat{S}}_{{\rm{HE}}}(t)$ matches the pooled oracle up to CKKS-level numerical noise; validation tolerances appear in section “Results”.

### Datasets

We evaluate our approach on two datasets:

• **NCCTG Lung Cancer** contains 228 observations with survival times and censoring indicators (status = 1 censored, =2 event).

• **Synthetic Breast Cancer (IKNL)** contains 60,000 synthetic observations designed to mimic real-world survival data.

### Partitioning and heterogeneity

Each dataset is horizontally partitioned among *K* clients. We first shuffle the full dataset (fixed seed for reproducibility) and split rows into *K* subsets of approximately equal size while retaining all features; this approximates an i.i.d. partition.

To study non-IID effects, we induce *outcome heterogeneity* via the event indicator. Before splitting we: (i) standardize the event/status column to binary event ∈ {0, 1} (map {1, 2} as 1 → 0 censored, 2 → 1 event; otherwise declared “event” values map to 1 and all others to 0); (ii) enforce strictly positive survival times (drop non-positive entries); and (iii) log configuration for reproducibility.

Let *K* be the number of clients. We split by event ∈ {0, 1} and allocate each group with symmetric Dirichlet weights (strong heterogeneity):

$${{\bf{w}}}^{(g)} \sim {\rm{Dirichlet}}({\underbrace{\alpha ,\ldots ,\alpha }}_{K}),\,\alpha =0.2,\,g\in \{0,1\}.$$

Rows in group *g* are randomly permuted and assigned multinomially with probabilities **w**^(*g*)^; we then enforce a “no-empty-clients” constraint by reassigning surplus rows until every client has at least one row (this may soften the strongest skews but preserves the heterogeneity type). As *α* → *∞* the split tends to i.i.d.

This construction preserves the centralized multiset of rows and hence the union of survival times *T*_all_. The event-time grid *T* ⊆ *T*_all_ derived from that union is therefore also preserved. At each *t*_*i*_ ∈ *T*, per-client at-risk and event counts sum to centralized totals. Consequently, under exact aggregation/decryption the federated KM equals the centralized estimator, and under CKKS it converges to it with negligible numerical error (section “Theoretical Results”). For plaintext baselines we also vary client-level overlap (None/Small/Large) to analyze reconstruction risk; these overlap settings do not affect the encrypted protocol.

### Evaluation protocol

We repeat each configuration 10 times to visualize curve variability, quantify numerical and statistical agreement between encrypted and pooled (oracle) pipelines, and measure overhead. We explicitly include non-IID label splits (Dirichlet *α* = 0.2) and overlap regimes to stress heterogeneity; as predicted by partition invariance (Theorem 5), the HE-federated Kaplan–Meier (KM) matches the pooled oracle under both IID and non-IID partitions. Data partitioning follows section “Partitioning and heterogeneity”.

### Single-machine environment and timing

All experiments run on a MacBook Pro (Apple M3 Pro, 36 GB RAM, macOS Sonoma 14.5). The experiments were implemented in Python 3.12.2 using OpenFHE (C++ back end) via Python bindings. Unless stated otherwise, the reported wall-clock runtime measures the end-to-end execution of the experimental benchmark pipeline, including cryptographic setup, federated aggregation, threshold decryption, Kaplan–Meier evaluation, and associated implementation overhead. Each point shows the sample mean with 95% CIs based on the normal approximation across 10 replicates.

### Homomorphic-encryption parameters

We use OpenFHE ^12^ multiparty (threshold) APIs for CKKS. Phase B (R2) performs slotwise additions (and occasional rotations) only; no ciphertext–ciphertext multiplications are used, so precision is governed by the initial scale (no rescaling/bootstrapping). Unless otherwise stated, experiments use the strict all-of-*R* setting *θ* = *R*; for fidelity and runtime studies we set *R* = *K*, while fixed-*R* variants are used only in communication-scaling studies.

In the current implementation, we encode at-risk counts and event counts over the full alignment grid *T*_all_. Using two layouts:

$$\begin{array}{rcl}{\bf{\text{Interleaved :}}}\,({n}_{1},{d}_{1},{n}_{2},{d}_{2},\ldots ) & \Rightarrow & {M}_{\mathrm{int}}=\left\lceil \frac{2|{T}_{\mathrm{all}}|}{B}\right\rceil ,\\ {\bf{\text{Separate :}}}\,({d}_{1},\ldots ,{d}_{|{T}_{\mathrm{all}}|})\,\mathrm{and}\,({n}_{1},\ldots ,{n}_{|{T}_{\mathrm{all}}|}) & \Rightarrow & {M}_{\mathrm{sep}}=2\left\lceil \frac{|{T}_{\mathrm{all}}|}{B}\right\rceil .\end{array}$$

If packing were instead performed on the event-time subset *T*, the same formulas would hold with ∣*T*∣ replacing ∣*T*_all_∣.

With *B* = 8192 the stepwise dependence on the packed grid length is:

$$L=1000:\,(1,2),\,L=5000:\,(2,2),\,L=10,000:\,(3,4)\,\,{\rm{for}}\,\,({M}_{{\rm{int}}},{M}_{{\rm{sep}}}),$$

where *L* denotes the grid length used for packing. In our implementation *L* = ∣*T*_all_∣. Interleaving hence gives a 2 × reduction when *L* ≤ *B*/2, ties at *L* ∈ (*B*/2, *B*], and a 4/3 × reduction on (*B*, 1.5*B*] (as detailed in Section S6 of the Supplementary Information).

We implement per-client uplink accounting. Encrypted payload per client in Phase B (R2) is

$${\rm{he}}\_{\rm{uplink}}\,=\,M\times {\mathtt{ct}}\_{\mathtt{bytes}}.$$

Decryptor-share payload is

$${\rm{shares}}\_{\rm{uplink}}\,=\,M\times R\times {\mathtt{share}}\_{\mathtt{bytes}}.$$

Thus total encrypted uplink to the coordinator is linear in *K* (sites), while share traffic is linear in *R* (decryptors) and independent of *K*.

For fidelity/statistics/runtime experiments we set *R* = *K* (all clients act as decryptors). For the *communication-scalability* study only, we fix *R* ∈ {5, 9, 25} independent of *K* to expose the *R*-linearity of share traffic. This changes communication tallies only; numerical accuracy is unaffected.

We compute restricted mean survival time (RMST(*τ*)) as a left-Riemann sum over the event-time grid *T* up to horizon *τ*:

$${\rm{RMST}}(\tau )=\mathop{\sum }\limits_{{t}_{i}\le \tau }\widehat{S}({t}_{i-1})\,\Delta {t}_{i},\,\Delta {t}_{i}={t}_{i}-{t}_{i-1},\,{t}_{0}=0.$$

Unless stated otherwise we set $\tau =\max T$.

We report two summary tolerances:

$$\mathop{\max }\limits_{t\in T}\left|{\widehat{S}}_{\mathrm{HE}}(t)-{\widehat{S}}_{\mathrm{oracle}}(t)\right|\le 1{0}^{-8},\,\frac{{\sum }_{t\in T}\left|{\widehat{S}}_{\mathrm{HE}}(t)-{\widehat{S}}_{\mathrm{oracle}}(t)\right|}{{\sum }_{t\in T}\left|{\widehat{S}}_{\mathrm{oracle}}(t)\right|}\le 1{0}^{-9}.$$

Bands use pooled Greenwood SE (coverage and standardized sup-gap); all figures show mean with 95% CIs across the 10 repeats. Table 7 summarizes the CKKS parameter instantiation used throughout the experiments. Table 8 summarizes the equivalence metrics used to compare the HE-federated Kaplan-Meier estimator with the pooled oracle estimator, together with their definitions and pre-specified pass thresholds.

**Table 7:** CKKS instantiation used throughout

Parameter	Value/Rationale
Scheme	CKKS (OpenFHE, multiparty/threshold APIs)
Ring degree *n*	16,384 (meets ≈128-bit RLWE security with our ∣*Q*∣)
Slot budget *B*	*n*/2 = 8192 complex slots per ciphertext
Total modulus ∣*Q*∣	≈438 bits (NTT-friendly RNS primes)
Initial scale	2^40^ (preserves ~9–10 decimal digits)
Galois keys	Powers-of-two rotations (no relinearization keys)
Ciphertext size	ct_bytes ≈ 1,794,048 bytes (measured)
Share size	share_bytes ≈ 897,024 bytes (measured)

**Table 8:** Equivalence metrics, definitions, and pass thresholds used to judge agreement between HE-federated and pooled (oracle) estimators

Acr.	Metric	Definition	Threshold
TAAE(*S*)	Time-avg ∣Δ*S*∣	TAAE(*S*) = IAE(*S*)/*τ*^a^	≤10^−9^
IAE(*S*)	Int ∣Δ*S*∣	${\rm{IAE}}(S)=\int | {\widehat{S}}_{{\rm{HE}}}(t)-{\widehat{S}}_{{\rm{oracle}}}(t)| \,dt$	≤10^−6^
∣Δ RMST∣	Abs RMST gap	∣RMST_HE_(*τ*) − RMST_oracle_(*τ*)∣	≤10^−6^
Δ RMST_rel_	Rel RMST gap	$\frac{| {{\rm{RMST}}}_{{\rm{HE}}}(\tau )-{{\rm{RMST}}}_{{\rm{oracle}}}(\tau )| }{{{\rm{RMST}}}_{{\rm{oracle}}}(\tau )}$	≤10^−6^
supZ	Supremum *z*	$\mathop{\sup }\limits_{t\in T}\frac{| {\hat{S}}_{{\rm{HE}}}(t)-{\hat{S}}_{{\rm{oracle}}}(t)| }{\widehat{{\rm{SE}}}[{\hat{S}}_{{\rm{oracle}}}(t)]}$	≤10^−6^
Coverage	95% coverage	$\frac{1}{| T| }{\sum }_{t\in T}{\bf{1}}\{\hat{{S}_{{\rm{HE}}}}(t)\in [{\hat{S}}_{{\rm{oracle}}}(t)\pm 1.96\,\widehat{{\rm{SE}}}(t)]\}$	≥0.99
$\mathop{\max }\limits_{t}| \Delta H|$	Max ∣Δ*H*∣	$\mathop{\max }\limits_{t\in T}| {H}_{{\rm{HE}}}(t)-{H}_{{\rm{oracle}}}(t)|$	≤10^−8^
IAE_*H*_	Int ∣Δ*H*∣	∫∣*H*_HE_(*t*) − *H*_oracle_(*t*)∣ *dt*	≤10^−7^

*τ* is the horizon; Greenwood SE for bands.

### Communication scalability

We analyze communication using closed-form counts consistent with section “Communication complexity”. Let *K* denote the number of sites, *T*_all_ the union of all unique survival times across sites (including censoring times), *T* ⊆ *T*_all_ the event-time grid used for Kaplan–Meier aggregation, *R* ≤ *K* the decryptor committee size, *n* the CKKS ring degree, and *B* = *n*/2 the slot capacity per ciphertext. Unless stated otherwise, payload sizes are reported in MB (decimal units, 10^6^ bytes).

**Plain protocol**. Our plaintext two-round protocol operates as follows. In Round 1 (R1), each site uploads its local set of unique observed survival times ${T}_{k}^{{\rm{all}}}$ (including both event and censoring times). The coordinator computes the union

$${T}_{\mathrm{all}}=\mathop{\bigcup }\limits_{k=1}^{K}{T}_{k}^{\mathrm{all}}$$

and broadcasts *T*_all_ to all sites. The event-time subset *T* = {*t* ∈ *T*_all_: ∃ an even tat *t*} is not constructed explicitly; it is handled implicitly because *d*_*i*_ = 0 at censoring-only times.

In Round 2 (R2), each site uploads aligned counts

$${\left\{\left({n}_{i}^{(k)},{d}_{i}^{(k)}\right)\right\}}_{{t}_{i}\in {T}_{\mathrm{all}}}.$$

The total number of uploaded plaintext scalars is therefore

$$\mathop{\underbrace{\mathop{\sum }\limits_{k=1}^{K}\left|{T}_{k}^{\mathrm{all}}\right|}}\limits_{{\rm{R}}1\mathrm{upload}}\,+\,\mathop{\underbrace{2K| {T}_{\mathrm{all}}| }}\limits_{{\rm{R}}2\mathrm{counts}(\mathrm{at}-\mathrm{riskandevents})}.$$

The downlink consists of broadcasting *T*_all_, giving

$$K| {T}_{{\rm{all}}}|$$

plaintext scalars in total.

The R1 uplink satisfies

$$\mathop{\sum }\limits_{k=1}^{K}\left|{T}_{k}^{\mathrm{all}}\right|\,\le \,K\left|{T}_{\mathrm{all}}\right|.$$

The R1 uplink bounds (in bytes, with 8 bytes per scalar) are therefore

$${{\rm{uplink}}}_{\min }^{{\rm{R1}}}=| {T}_{{\rm{all}}}| \cdot 8,\,{{\rm{uplink}}}_{\max }^{{\rm{R1}}}=K| {T}_{{\rm{all}}}| \cdot 8.$$

Combining R1 and R2 yields the min–max uplink bounds

$${{\rm{uplink}}}_{\min }=(| {T}_{{\rm{all}}}| +2K| {T}_{{\rm{all}}}| )\cdot 8,\,{{\rm{uplink}}}_{\max }=(K| {T}_{{\rm{all}}}| +2K| {T}_{{\rm{all}}}| )\cdot 8=3K| {T}_{{\rm{all}}}| \cdot 8.$$

The total downlink is

$$K| {T}_{{\rm{all}}}| \cdot 8.$$

*Event-time-only variant*. If a deployment filters to the event-time subset *T* before R2, the R2 uplink reduces to 2*K*∣*T*∣ scalars; R1 communication is unchanged.

**CKKS with multiparty decryption**. In the encrypted protocol we retain R1 in plaintext and encrypt only R2. In the current implementation, each site encrypts two length-∣*T*_all_∣ vectors containing at-risk and event counts aligned to the full grid *T*_all_, with *d*_*i*_ = 0 at non-event times. With CKKS packing and slot capacity *B* = *n*/2, the number of ciphertexts per client is

$$M=\left\{\begin{array}{ll}2\lceil | {T}_{\mathrm{all}}| /B\rceil , & \mathrm{separate}\,\mathrm{packing},\\ \lceil 2| {T}_{\mathrm{all}}| /B\rceil , & \mathrm{interleaved}\,\mathrm{packing}.\end{array}\right.$$

If encryption were applied only to the event-time subset *T*, the same formulas would hold with ∣*T*∣ replacing ∣*T*_all_∣. This is not the current implementation; rather, it is an event-time-only packing variant used for the communication simulations in section “Results”, which instantiate these formulas with *L* = ∣*T*∣.

Using the conservative byte model

$${\mathtt{ct}}\_{\mathtt{bytes}}\approx \frac{2n| Q| }{8},\,{\mathtt{share}}\_{\mathtt{bytes}}\approx \frac{n| Q| }{8},$$

the encrypted R2 uplink is

$${\rm{HE}}\,{\rm{R2}}\,{\rm{uplink}}=KM\cdot {\mathtt{ct}}\_{\mathtt{bytes}},$$

and the decryption-share traffic is

$${\mathrm{shares}}\,{\mathrm{uplink}}=R\,M\cdot {\mathtt{share}}\_{\mathtt{bytes}}.$$

Dividing by 10^6^ converts these values to MB. We instantiate these formulas on the grid in Table 9 and report: (i) plaintext uplink and downlink including the R1 min–max band, (ii) encrypted R2 uplink in MB, and (iii) decryption-share traffic in MB.

**Table 9:** Parameter grid used to instantiate the communication formulas

Symbol	Description	Values (grid)
*K*	Number of clients	{10, 50, 100, 200, 300, 500}
∣*T*_all_∣	Number of global unique survival times	depends on dataset and censoring pattern
∣*T*∣	Number of global event times	{1000, 5000, 10,000}
*R*	Committee size	{5, 9, 25}
*n*	CKKS ring degree	{16,384}
∣*Q*∣	Total modulus bitlength	{438} bits

**Assumptions**. The analysis assumes: (i) payload-only accounting with transport and TLS framing omitted, (ii) R1 remains plaintext in both the plain and HE protocols, (iii) R2 uses add-only encrypted aggregation, (iv) ciphertext sizes follow the uncompressed model above, which gives a conservative upper bound, and (v) sites align on a common global alignment grid *T*_all_, from which the event-time grid *T* used for Kaplan–Meier evaluation is derived.

In the current implementation, R2 uses *T*_all_ as the alignment grid passed to each client, so encrypted count vectors are packed over *T*_all_ rather than over the event-time subset *T*. Counts are therefore uploaded for every time in *T*_all_, with *d*_*i*_ = 0 at non-event times. This does not change the Kaplan–Meier estimator because inserting zero-event time points refines the grid without altering the product. However, the encrypted R2 uplink in the implementation scales with *M*(∣*T*_all_∣) rather than *M*(∣*T*∣). Filtering to event times before encryption would therefore reduce the number of ciphertexts whenever ∣*T*_all_∣ > ∣*T*∣.

## Supplementary information

Supplementary Information

## Data Availability

The NCCTG lung cancer dataset used in this study is publicly available in the R survival package and can be accessed at https://rdrr.io/cran/survival/man/lung.html. For our experiments, we downloaded the dataset as a CSV file and processed it using Python. The synthetic breast cancer dataset used in this study is a controlled-access synthetic dataset provided by IKNL. Information about access is available at https://iknl.nl/international/data-request. Researchers may request access from IKNL in accordance with its data request procedures.
