# Supplementary material for: A multiparty homomorphic encryption approach to confidential federated Kaplan–Meier survival analysis
Source: NPJ Digit Med. 2026 Jun 8;9:775. doi: 10.1038/s41746-026-02790-4 (PMC13645743; doi:10.1038/s41746-026-02790-4)
Supplement: Supplementary file 1 [file 41746_2026_2790_MOESM1_ESM.pdf]

Supplementary Proofs for:
A Multiparty Homomorphic Encryption Approach to Confidential Federated Kaplan–Meier Survival Analysis

Overview

This document provides complete proofs for all theorems, lemmas, propositions, and corollaries stated in the main manuscript, with the exception of the security analysis results (Phase A, B, C privacy theorems and the end-to-end privacy corollary), whose proofs already appear in the main text. We preserve the notation and conventions of the main paper; every theorem-like statement below is identical to its counterpart in the main text.

Standing conventions (reproduced from the main paper).

- K sites hold a horizontal partition of a fixed centralized multiset of rows (IID or non-IID, with or without overlap). "Horizontal partition" means each row of the multiset is assigned to exactly one site; "overlap" refers to the possibility that the underlying data-generating process produces duplicate records across institutions, so the centralized multiset may contain copies of the same physical record. Under this convention the partition is disjoint over the multiset.
- $T_{\text{all}} = \bigcup_{k=1}^{K} T_k^{\text{all}}$ is the global alignment grid; $T = \{t \in T_{\text{all}} : \exists \text{ an event at } t\} = \bigcup_{k=1}^{K} T_k^{\text{event}}$ is the event-time grid with $|T|$ points. The sets T_k^{event} and T are theoretical objects used in KM analysis; the current implementation uploads only T_k^{all}, constructs only T_{all}, and handles T implicitly because $d_i = 0$ at censoring-only times.
- For each $t_i \in T$, $d_i^{(k)}$ is the number of events and $n_i^{(k)}$ is the number at risk at site k; aggregates are $d_i = \sum_k d_i^{(k)}$ and $n_i = \sum_k n_i^{(k)}$.
- $h_i = d_i/n_i$ is the discrete hazard at t_i; $H(t) = \sum_{t_i \le t} h_i$ is the cumulative hazard.
- $\hat{S}^\star(t) = \prod_{t_i \le t}(1 - h_i)$, $\quad n_i > 0$, is the pooled Kaplan–Meier estimator.
- All sites share the same alignment grid and tie convention: n_i is the count at risk just *before* t_i; events are applied at t_i; censorings at t_i affect only subsequent risk sets.
- CKKS slot capacity is $B = n/2$; L is the packed grid length. In the current implementation $L = |T_{\text{all}}|$; in an event-time-only variant $L = |T|$.
- $M_0 = \lceil L/B \rceil,\ M_{\text{int}} = \lceil 2L/B \rceil,\ M_{\text{sep}} = 2\lceil L/B \rceil$.

S1 Model and Basic Identities

Lemma 1 (Global grid invariance)**.** *For any horizontal partition of a fixed centralized multiset of rows (IID or non-IID, with or without overlap), the union of all unique observed times*

$$T_{\text{all}} = \bigcup_{k=1}^{K} T_k^{\text{all}}$$

is invariant. The derived event-time grid

$$T = \{\, t \in T_{\text{all}} : \exists \textit{ an event at } t \,\}$$

and its cardinality $|T|$ are therefore also invariant.

Proof. The centralized multiset of rows is fixed. Each row carries an observed time value and an event indicator. Under a horizontal partition, each row of the multiset is assigned to exactly one site k. (When the multiset contains duplicate records, each copy is assigned independently; the multiset itself is unchanged.)

Step 1: Invariance of T_{all}. Let $\mathcal{T}$ denote the set of distinct observed time values appearing in at least one row of the centralized multiset. Under any partition, every row with time value $t \in \mathcal{T}$ is assigned to some site k, so $t \in T_k^{\text{all}} \subseteq T_{\text{all}}$. Conversely, every $t \in T_{\text{all}}$ must originate from some row in the multiset, so $t \in \mathcal{T}$. Hence $T_{\text{all}} = \mathcal{T}$, which is determined entirely by the centralized multiset and is therefore partition-invariant.

Step 2: Invariance of T. A time $t \in T_{\text{all}}$ belongs to T if and only if at least one row in the centralized multiset has observed time equal to t and event indicator equal to 1. This condition depends only on the centralized multiset, not on how its rows are distributed among sites. Hence T is partition-invariant, and so is $|T|$. □

Lemma 2 (Additivity of counts)**.** *For every $t_i \in T$, if local counting rules are aligned to the same global grid and tie convention, then*

$$d_i = \sum_{k=1}^{K} d_i^{(k)} \qquad \textit{and} \qquad n_i = \sum_{k=1}^{K} n_i^{(k)}.$$

Proof. Under the standing convention, a horizontal partition assigns each row of the centralized multiset to exactly one site. (If the underlying data contains duplicate records across institutions, those duplicates appear as distinct rows in the centralized multiset and are assigned independently; see the overlap discussion in the standing conventions.)

Because all sites use the same global grid T_{all} and the same tie convention:

(i) $d_i^{(k)}$ counts the rows assigned to site k with observed time equal to t_i and event indicator 1. Since every row of the multiset is assigned to exactly one site, the sets of rows counted by $d_i^{(1)}, \ldots, d_i^{(K)}$ are pairwise disjoint and their union is the set of all rows in the multiset with time t_i and event 1. Therefore $\sum_k d_i^{(k)} = d_i$.

(ii) $n_i^{(k)}$ counts the rows assigned to site k with observed time $\geq t_i$. By the same disjointness argument, $\sum_k n_i^{(k)} = n_i$. □

Lemma 3 (Grid refinement invariance)**.** *Let T be the set of event times and let $\tilde{T} \supseteq T$ be any refinement obtained by inserting time points with zero events. With consistent tie conventions, the KM estimator evaluated on $\tilde{T}$ equals the KM estimator on T at all $t \in T$.*

Proof. The Kaplan–Meier estimator on a time grid G is

$$\hat{S}_G(t) = \prod_{\substack{t_j \in G \\ t_j \leq t}} \left(1 - \frac{d_j}{n_j}\right), \qquad n_j > 0.$$

Write $\tilde{T} = T \cup T'$ where $T' = \tilde{T} \setminus T$ consists of time points at which no events occur, i.e. $d_j = 0$ for all $t_j \in T'$. Every $t_j \in T'$ appears in the data (otherwise it would not be in $\tilde{T}$), so $n_j \geq 1 > 0$ and the factor $1 - d_j/n_j = 1 - 0/n_j = 1$ is well-defined.

Therefore, for any $t \in T$,

$$\hat{S}_{\tilde{T}}(t) = \prod_{\substack{t_j \in \tilde{T} \\ t_j \leq t}} \left(1 - \frac{d_j}{n_j}\right) = \underbrace{\prod_{\substack{t_j \in T' \\ t_j \leq t}} 1}_{=1} \cdot \prod_{\substack{t_j \in T \\ t_j \leq t}} \left(1 - \frac{d_j}{n_j}\right) = \hat{S}_T(t).$$

It remains to verify that the at-risk counts at event times $t_i \in T$ are unchanged by refinement. By the standing tie convention, n_i is the number of individuals with observed time $\geq t_i$. Inserting additional grid points does not change which individuals satisfy this condition, so n_i is unaffected. □

S2 Correctness Theorems

Theorem 4 (Plain federated KM equals pooled oracle)**.** *Running R1–R2 in plaintext yields* $\hat{S}_{\text{plain}}(t) \equiv \hat{S}^{\star}(t)$ *for any partition of the same centralized multiset of rows.*

Proof. Round 1 (R1). Each site k uploads T_k^{all} to the coordinator, which forms $T_{\text{all}} = \bigcup_k T_k^{\text{all}}$. By Lemma 1, T_{all} is partition-invariant, and the theoretical event-time grid $T = \{t \in T_{\text{all}} : \exists \text{ an event at } t\}$ is therefore also partition-invariant.

Round 2 (R2). Each site k computes local counts $(d_i^{(k)}, n_i^{(k)})$ for $t_i \in T_{\text{all}}$ and uploads them. The coordinator aggregates: $d_i = \sum_k d_i^{(k)}$, $n_i = \sum_k n_i^{(k)}$. By Lemma 2, these equal the centralized counts.

The plaintext federated KM estimator is $\hat{S}_{\text{plain}}(t) = \prod_{t_i \leq t}(1 - d_i/n_i) = \hat{S}^{\star}(t)$, where the product may be taken over T_{all} or equivalently over T (by Lemma 3, since $d_i = 0$ at non-event times). □

Theorem 5 (Exact HE yields identical KM)**.** *If R2 uses exact homomorphic addition and threshold decryption returns the exact aggregated counts, then* $\hat{S}_{\text{HE-exact}}(t) \equiv \hat{S}^{\star}(t)$ *for any partition. This remains true whether alignment is performed directly on* T *or on any refinement* $\tilde{T} \supseteq T$*, including* T_{all}*.*

Proof. Step 1: Aggregation is exact. Each site k encodes its local count vectors $(d_i^{(k)}, n_i^{(k)})$ into plaintext vectors and encrypts them under the joint public key. The coordinator computes homomorphic sums $\mathsf{ct}_{\Sigma} = \bigoplus_{k=1}^{K} \mathsf{ct}^{(k)}$. Under exact homomorphic addition, the underlying plaintext is the componentwise sum. Threshold decryption recovers this plaintext exactly. By Lemma 2, the recovered counts equal the centralized aggregates.

Step 2: Alignment on T. With exact counts on the event-time grid T, the estimator is $\hat{S}_{\text{HE-exact}}(t) = \prod_{t_i \leq t}(1 - d_i/n_i) = \hat{S}^{\star}(t)$.

Step 3: Alignment on a refinement. If alignment uses $\tilde{T} \supseteq T$ (e.g. T_{all}), the count vectors include $d_j = 0$ at non-event times. Exact aggregation preserves these zeros. By Lemma 3, the KM estimator on $\tilde{T}$ equals $\hat{S}^{\star}(t)$ at all $t \in T$. □

S3 CKKS Perturbation Bound and Uniform Convergence

We model CKKS and threshold outputs on the event-time grid as $\widehat{d_i} = d_i + \Delta d_i$ and $\widehat{n}_i = n_i + \Delta n_i$ with deterministic error envelopes $\varepsilon_d(i) \geq |\Delta d_i|$ and $\varepsilon_n(i) \geq |\Delta n_i|$.

We first state a helper lemma used in the main proof.

Lemma 6 (Multiplicative product bound)**.** *For any reals* $r_1, \ldots, r_m$*,*

$$\left|\prod_{i=1}^{m}(1 - r_i) - 1\right| \;\leq\; \exp\!\Big(\sum_{i=1}^{m}|r_i|\Big) - 1.$$

Proof. By induction on m.

Base case ($m = 1$). $|1 - r_1 - 1| = |r_1| \leq e^{|r_1|} - 1$, since $e^x - 1 \geq x$ for all $x \geq 0$.

Inductive step. Let $P_m = \prod_{i=1}^{m}(1 - r_i)$ and $S_m = \sum_{i=1}^{m} |r_i|$. Suppose $|P_m - 1| \leq e^{S_m} - 1$. Then $|P_m| \leq 1 + |P_m - 1| \leq e^{S_m}$. Now

$$\begin{aligned}
|P_{m+1} - 1| &= |P_m(1 - r_{m+1}) - 1| \\
&= |P_m - 1 - P_m r_{m+1}| \\
&\leq |P_m - 1| + |P_m|\,|r_{m+1}| \\
&\leq (e^{S_m} - 1) + e^{S_m}\,|r_{m+1}| \\
&= e^{S_m}(1 + |r_{m+1}|) - 1 \\
&\leq e^{S_m} \cdot e^{|r_{m+1}|} - 1 = e^{S_{m+1}} - 1,
\end{aligned}$$

where the last inequality uses $1 + x \leq e^x$ for $x \geq 0$. □

Theorem 7 (CKKS perturbation bound)**.** *Assume* $0 \leq \varepsilon_n(i) < n_i$ *(so* $\widehat{n}_i > 0$*); define* $h_i = d_i/n_i$ *and* $h_{\max} = \max_{t_j \leq t} h_j < 1$*. Set*

$$\delta_i \;:=\; \frac{\varepsilon_d(i)}{n_i - \varepsilon_n(i)} \;+\; \frac{d_i}{n_i} \cdot \frac{\varepsilon_n(i)}{n_i - \varepsilon_n(i)}.$$

Then, for all t*,*

$$\left|\hat{S}_{\text{HE-ckks}}(t) - \hat{S}^{\star}(t)\right| \;\leq\; \hat{S}^{\star}(t)\left(\exp\!\Big(\frac{1}{1 - h_{\max}} \sum_{t_i \leq t} \delta_i\Big) - 1\right).$$

Proof. Write $\widehat{d}_i = d_i + \Delta d_i$ and $\widehat{n}_i = n_i + \Delta n_i$ with $|\Delta d_i| \leq \varepsilon_d(i)$ and $|\Delta n_i| \leq \varepsilon_n(i)$. Define the perturbed hazard $\widehat{h}_i = \widehat{d}_i/\widehat{n}_i$.

Step 1: Ratio representation. The perturbed estimator satisfies $\hat{S}_{\text{HE-ckks}}(t) = \prod_{t_i \leq t}(1 - \widehat{h}_i)$. Define

$$r_i := \frac{\widehat{h}_i - h_i}{1 - h_i},$$

so that $(1 - \widehat{h}_i)/(1 - h_i) = 1 - r_i$ and $\hat{S}_{\text{HE-ckks}}(t)/\hat{S}^{\star}(t) = \prod_{t_i \leq t}(1 - r_i)$.

Step 2: Hazard perturbation bound.

$$\widehat{h}_i - h_i = \frac{d_i + \Delta d_i}{n_i + \Delta n_i} - \frac{d_i}{n_i} = \frac{n_i \Delta d_i - d_i \Delta n_i}{n_i(n_i + \Delta n_i)}.$$

Since $0 \leq \varepsilon_n(i) < n_i$ implies $n_i + \Delta n_i \geq n_i - \varepsilon_n(i) > 0$, we have $|n_i + \Delta n_i| \geq n_i - \varepsilon_n(i)$. Applying the triangle inequality to the numerator:

$$\begin{aligned}
|\widehat{h}_i - h_i| &\leq \frac{n_i\,\varepsilon_d(i) + d_i\,\varepsilon_n(i)}{n_i\,(n_i - \varepsilon_n(i))} \\
&= \frac{\varepsilon_d(i)}{n_i - \varepsilon_n(i)} + \frac{d_i}{n_i} \cdot \frac{\varepsilon_n(i)}{n_i - \varepsilon_n(i)} \;=\; \delta_i. \qquad (1)
\end{aligned}$$

Step 3: Bound on $|r_i|$*.* From Step 2,

$$|r_i| = \frac{|\widehat{h}_i - h_i|}{1 - h_i} \leq \frac{\delta_i}{1 - h_i} \leq \frac{\delta_i}{1 - h_{\max}},$$

where the last inequality uses $h_i \leq h_{\max}$.

Step 4: Product bound. By Lemma 6,

$$\left|\frac{\hat{S}_{\text{HE-ckks}}(t)}{\hat{S}^{\star}(t)} - 1\right| = \left|\prod_{t_i \leq t}(1 - r_i) - 1\right| \leq \exp\!\Big(\sum_{t_i \leq t} |r_i|\Big) - 1 \leq \exp\!\Big(\frac{1}{1 - h_{\max}} \sum_{t_i \leq t} \delta_i\Big) - 1.$$

Step 5: Conclusion. Multiplying both sides by $\hat{S}^\star(t) \geq 0$:

$$|\hat{S}_{\text{HE-ckks}}(t) - \hat{S}^\star(t)| \leq \hat{S}^\star(t) \left(\exp\Big(\frac{1}{1-h_{\max}} \sum_{t_i \leq t} \delta_i \Big) - 1 \right). \qquad \square$$

Remark 8 (Terminal jump with $h_i = 1$). If $d_i = n_i$ at a terminal time point (so $h_i = 1$ and $\hat{S}^\star$ drops to 0), the stated bound applies on indices with $h_j < 1$. At and after the terminal jump, the trivial bound $|\hat{S}_{\text{HE-ckks}}(t) - \hat{S}^\star(t)| = \hat{S}_{\text{HE-ckks}}(t)$ holds.

Corollary 9 (Uniform convergence on T). *If $\varepsilon_d(i), \varepsilon_n(i) \to 0$ for all $t_i \in T$, then $\hat{S}_{\text{HE-ckks}}(t) \to \hat{S}^\star(t)$ uniformly on T.*

Proof. Let $\bar{h} := \max_{t_j \in T} h_j$. We treat the two cases $\bar{h} < 1$ and $\bar{h} = 1$ separately.

Case 1: $\bar{h} < 1$ (no terminal jump). By definition, $\delta_i = (\varepsilon_d(i) + h_i\,\varepsilon_n(i))/(n_i - \varepsilon_n(i))$. As $\varepsilon_d(i) \to 0$ and $\varepsilon_n(i) \to 0$ with $n_i > 0$ and $h_i \geq 0$ fixed, each $\delta_i \to 0$. Since $|T|$ is finite, $\sum_{t_i \in T} \delta_i \to 0$.

For every $t \in T$, Theorem 7 gives

$$|\hat{S}_{\text{HE-ckks}}(t) - \hat{S}^\star(t)| \leq \hat{S}^\star(t) \left(\exp\Big(\frac{1}{1-\bar{h}} \sum_{t_i \in T} \delta_i \Big) - 1 \right) \leq \exp\Big(\frac{1}{1-\bar{h}} \sum_{t_i \in T} \delta_i \Big) - 1,$$

using $h_{\max}(t) \leq \bar{h}$ for all t and $\hat{S}^\star(t) \leq 1$. The right-hand side is independent of t and tends to 0, so the convergence is uniform on T.

Case 2: $\bar{h} = 1$ (terminal jump). Let $t_\tau \in T$ be the first event time at which $h_\tau = 1$ (i.e. $d_\tau = n_\tau$). For every $t < t_\tau$, $h_{\max}(t) < 1$, so Case 1 applies and $|\hat{S}_{\text{HE-ckks}}(t) - \hat{S}^\star(t)| \to 0$. For every $t \geq t_\tau$, $\hat{S}^\star(t) = 0$ because the product includes the factor $1 - h_\tau = 0$. Hence $|\hat{S}_{\text{HE-ckks}}(t) - \hat{S}^\star(t)| = |\hat{S}_{\text{HE-ckks}}(t)|$. Now

$$\hat{S}_{\text{HE-ckks}}(t) = \prod_{t_i \leq t} (1 - \widehat{h}_i).$$

As $\varepsilon_d(\tau), \varepsilon_n(\tau) \to 0$, the perturbed hazard $\widehat{h}_\tau \to h_\tau = 1$, so $1 - \widehat{h}_\tau \to 0$. Because every other factor satisfies $|1 - \widehat{h}_i| \leq 1 + |\widehat{h}_i|$, which is bounded (since all perturbed hazards converge to their finite limits), the product tends to 0. Hence $|\hat{S}_{\text{HE-ckks}}(t)| \to 0$ for every $t \geq t_\tau$.

Combining the two sub-cases: $|\hat{S}_{\text{HE-ckks}}(t) - \hat{S}^\star(t)| \to 0$ at every $t \in T$. Since T is finite, pointwise convergence on T is equivalent to uniform convergence on T. $\square$

S4 Identifiability from Public Outputs

Proposition 10 (Hazards are identified; counts are not). *Assume T contains the event times and $\hat{S}(0) = 1$ is known. Then the per-time-point hazard ratios are determined by*

$$h_i = 1 - \frac{\hat{S}(t_i)}{\hat{S}(t_{i-1})}.$$

However, without additional side information (e.g., initial at-risk size, censoring schedule), the integer pairs (d_i, n_i) consistent with these ratios are not unique; there exist infinitely many real-valued (d_i, n_i) solutions and, typically, multiple integer completions. Hence publishing $\hat{S}(t)$ alone reveals d_i/n_i but not (d_i, n_i) nor any per-site split.

Proof. Part 1: Hazards are determined. The KM estimator is a step function with jumps only at event times, satisfying $\hat{S}(t_0) = 1$ and $\hat{S}(t_i) = \hat{S}(t_{i-1})(1 - h_i)$ for each $t_i \in T$. Since $\hat{S}(t_{i-1}) > 0$ whenever $h_j < 1$ for all $j < i$,

$$h_i = 1 - \frac{\hat{S}(t_i)}{\hat{S}(t_{i-1})}$$

is uniquely determined from the published curve values at consecutive event times.

Part 2: Counts are not determined (per-time-point). At each t_i, knowing $h_i = d_i/n_i$ constrains (d_i, n_i) to the ray $\{(\lambda h_i, \lambda) : \lambda > 0\}$. Any $\lambda > 0$ gives a valid real-valued pair, so there are infinitely many. For integer solutions with $h_i = p/q$ in lowest terms, every $(d_i, n_i) = (mp, mq)$ with $m \in \mathbb{Z}_{>0}$ is valid. Without the initial at-risk count or censoring schedule, the multiplier m cannot be recovered from h_i alone.

Part 3: Full-table and per-site non-uniqueness. For a full KM-compatible table $(d_1, n_1), \ldots, (d_{|T|}, n_{|T|})$, the at-risk counts satisfy the recursion $n_{i+1} = n_i - d_i - c_i$ where $c_i \geq 0$ denotes the number of censorings in $[t_i, t_{i+1})$. Knowing all h_i values fixes the *ratios* d_i/n_i but not the initial at-risk count n_1 nor the censoring counts $c_1, \ldots, c_{|T|-1}$. Different choices of $(n_1, c_1, \ldots, c_{|T|-1})$ that are mutually consistent with the hazard ratios yield distinct full tables. Since the initial at-risk count n_1 is a free positive-integer parameter and each c_i is a free nonnegative-integer parameter, multiple full-table completions exist in general.

Even if the aggregate (d_i, n_i) were known, the decomposition $d_i = \sum_k d_i^{(k)}$ with $d_i^{(k)} \geq 0$ admits many solutions for $K \geq 2$. Publishing $\hat{S}(t)$ alone reveals only h_i, which provides strictly less information than the aggregate counts. □

S5 Noise Envelopes and Utility Propositions

We restate the noise model from the main paper.

Assumption 1 (Add-only CKKS noise and share aggregation)**.** *(i) For a ciphertext contributed by site k using M_k ciphertext blocks at fixed scale, the encode–encrypt perturbation satisfies $\|\Delta_k(\cdot)\| = O(M_k \log q_{\max})$, where $q_{\max} := \max_\ell q_\ell$ is the largest RNS limb modulus.*

(ii) Threshold-share fusion aggregates zero-mean, independent share errors across decryptors with variance proxy σ^2, contributing $O(\sigma\sqrt{R})$ to each block's error.

Proposition 11 (Utility envelope: coarse worst case)**.** *Under Assumption 1, there exist non-negative $\varepsilon_d(i), \varepsilon_n(i)$ with*

$$\varepsilon_d(i), \varepsilon_n(i) \in O\big(\log(q_{\max}) \textstyle\sum_{k=1}^K M_k\big) + O(\sigma\sqrt{R})$$

for which

$$\Delta S(t) := \big|\hat{S}^\star(t) - \hat{S}_{\mathrm{HE}}(t)\big| \leq \hat{S}^\star(t) \cdot \sum_{t_i \leq t} \frac{O\big(\log(q_{\max}) \sum_k M_k\big) + O(\sigma\sqrt{R})}{n_i - \varepsilon_n(i)},$$

reducing to the large-n_i form with denominator n_i.

Proof. Under Assumption 1, the total perturbation on each slot after homomorphic addition and threshold fusion comprises two additive components. Encryption noise sums linearly over sites (by linearity of homomorphic addition), contributing $O(\sum_{k=1}^K M_k \log q_{\max})$ per slot. Fusion of R independent share errors with variance proxy σ^2 contributes $O(\sigma\sqrt{R})$. Define

$$\varepsilon := C\Big(\log(q_{\max}) \sum_{k=1}^K M_k + \sigma\sqrt{R}\Big)$$

for a sufficiently large constant $C > 0$ (depending on the CKKS parameter set but not on K, R, or the data), and set $\varepsilon_d(i) = \varepsilon_n(i) = \varepsilon$ for all i. Then $|\Delta d_i| \le \varepsilon$ and $|\Delta n_i| \le \varepsilon$.

Apply Theorem 7 with these envelopes. The δ_i is

$$\delta_i = \frac{\varepsilon + h_i\,\varepsilon}{n_i - \varepsilon} = \frac{\varepsilon(1+h_i)}{n_i - \varepsilon} \le \frac{2\varepsilon}{n_i - \varepsilon},$$

using $h_i \le 1$. The CKKS bound gives

$$\Delta S(t) \le \hat{S}^\star(t) \left(\exp\Big(\frac{1}{1-h_{\max}} \sum_{t_i \le t} \frac{2\varepsilon}{n_i - \varepsilon} \Big) - 1 \right).$$

Write $n_{\min} := \min_{t_i \in T} n_i$ and assume $\varepsilon < n_{\min}/2$ (which holds for sufficiently small CKKS noise). Then each term satisfies $\varepsilon/(n_i - \varepsilon) \le 2\varepsilon/n_i$, so the exponent is at most

$$x_0 := \frac{4\,|T|\,\varepsilon}{(1-h_{\max})\,n_{\min}},$$

which is finite for fixed CKKS parameters (ring degree n, modulus chain $|Q|$), fixed committee size R, and finite event-time grid T. Applying $e^x - 1 \le x\,e^x$ for $x \ge 0$ with $x \le x_0$:

$$\Delta S(t) \le \hat{S}^\star(t) \cdot \frac{C'}{1-h_{\max}} \sum_{t_i \le t} \frac{\varepsilon}{n_i - \varepsilon}$$

for $C' := e^{x_0} > 0$. For fixed CKKS parameters, fixed R, and finite T, the quantities $C'/(1-h_{\max})$ and C' are bounded constants independent of the summation index, so they can be absorbed into the $O(\cdot)$ notation. Therefore

$$\Delta S(t) \le \hat{S}^\star(t) \cdot \sum_{t_i \le t} \frac{O(\varepsilon)}{n_i - \varepsilon},$$

which is the stated bound with $O(\varepsilon) = O(\log(q_{\max}) \sum_k M_k) + O(\sigma\sqrt{R})$ and denominator $n_i - \varepsilon_n(i) = n_i - \varepsilon$.

For the large-n_i form: when $\varepsilon \ll n_i$, $n_i - \varepsilon = n_i(1-\varepsilon/n_i)$, so $1/(n_i - \varepsilon) = (1 + O(\varepsilon/n_i))/n_i = O(1/n_i)$. Hence the summands reduce to $O(\varepsilon)/n_i$. □

Proposition 12 (Bounded at-risk counts). *If $n_i \ge c > 0$ for all t_i, then*

$$\Delta S(t) \le \frac{1}{c} \sum_{t_i \le t} \frac{O\big(\log(q_{\max}) \sum_k M_k\big) + O(\sigma\sqrt{R})}{1 - \big(O\big(\log(q_{\max}) \sum_k M_k\big) + O(\sigma\sqrt{R})\big)/\,n_i},$$

which further reduces to

$$\frac{1}{c} \sum_{t_i \le t} \Big(O\Big(\log(q_{\max}) \sum_k M_k\Big) + O(\sigma\sqrt{R}) \Big)$$

for large n_i.

Proof. From Proposition 11 with $\hat{S}^\star(t) \le 1$ and writing $\varepsilon = O(\log(q_{\max}) \sum_k M_k) + O(\sigma\sqrt{R})$:

$$\Delta S(t) \le \sum_{t_i \le t} \frac{O(\varepsilon)}{n_i - \varepsilon}.$$

Factor the denominator: $n_i - \varepsilon = n_i(1 - \varepsilon/n_i)$, so

$$\frac{O(\varepsilon)}{n_i - \varepsilon} = \frac{O(\varepsilon)}{n_i} \cdot \frac{1}{1 - \varepsilon/n_i}.$$

Since $n_i \geq c$ and $O(\varepsilon)$ absorbs constants:

$$\frac{O(\varepsilon)}{n_i} \leq \frac{O(\varepsilon)}{c}.$$

Combining, each summand is bounded by $\frac{O(\varepsilon)}{c} \cdot \frac{1}{1-\varepsilon/n_i}$, giving

$$\Delta S(t) \leq \frac{1}{c} \sum_{t_i \leq t} \frac{O(\varepsilon)}{1 - O(\varepsilon)/n_i}.$$

For large n_i (i.e. $\varepsilon \ll n_i$), $1/(1 - \varepsilon/n_i) \to 1$, so the bound reduces to $(1/c) \sum_{t_i \leq t} O(\varepsilon)$. □

Proposition 13 (Error scaling with client count)**.** *Under Assumption 1,*

$$\varepsilon_{\text{total}} = \sum_{k=1}^{K} O(M_k \log q_{\max}) + O(\sigma\sqrt{R}).$$

Proof. The aggregated ciphertext for a single block is the homomorphic sum of K individual ciphertexts. By Assumption 1(i), site k contributes $O(M_k \log q_{\max})$ noise per slot. Since homomorphic addition adds the noise components, the aggregated encryption noise is $\sum_{k=1}^{K} O(M_k \log q_{\max})$.

By Assumption 1(ii), threshold fusion adds an independent $O(\sigma\sqrt{R})$ term from aggregating R independent share errors with standard deviation σ. Since the two noise sources enter additively (encryption noise into the ciphertext, fusion noise at decryption), the total perturbation per slot is their sum. □

S6 Packing Optimality

Lemma 14 (Packing lower bound and interleaving optimality)**.** *Any packing that avoids cross-ciphertext multiplies and carries both streams over length L requires at least $\lceil 2L/B \rceil$ ciphertexts. Interleaved co-packing attains this bound with $M_{\text{int}} = \lceil 2L/B \rceil$, while separate packing uses $M_{\text{sep}} = 2\lceil L/B \rceil \geq \lceil 2L/B \rceil$.*

Proof. Lower bound. The two streams $(d_1, \dots, d_L)$ and $(n_1, \dots, n_L)$ comprise $2L$ scalar values. Each ciphertext provides B slots. Under add-only aggregation (no cross-ciphertext multiplies or rotations combining values from different ciphertexts), each of the $2L$ values must occupy at least one slot. By the pigeonhole principle, the minimum number of ciphertexts is $\lceil 2L/B \rceil$.

Interleaving attains the bound. Interleaved packing assigns the $2L$ values $n_1, d_1, n_2, d_2, \dots, n_L, d_L$ sequentially to slots, filling B slots per ciphertext, using exactly $\lceil 2L/B \rceil$ ciphertexts.

Separate packing uses weakly more. Separate packing assigns the d-stream to $\lceil L/B \rceil$ ciphertexts and the n-stream to another $\lceil L/B \rceil$, for $M_{\text{sep}} = 2\lceil L/B \rceil$.

Inequality $2\lceil x \rceil \geq \lceil 2x \rceil$. Write $x = m + f$ with $m = \lfloor x \rfloor$ and $f \in [0, 1)$. Then $\lceil x \rceil = m + \lceil f \rceil$ and $\lceil 2x \rceil = 2m + \lceil 2f \rceil$. We verify $2\lceil f \rceil \geq \lceil 2f \rceil$ by cases: if $f = 0$, both sides are 0; if $0 < f \leq 1/2$, $\lceil 2f \rceil = 1 \leq 2 = 2\lceil f \rceil$; if $1/2 < f < 1$, $\lceil 2f \rceil = 2 = 2\lceil f \rceil$. In every case, $2\lceil x \rceil \geq \lceil 2x \rceil$. Applying this with $x = L/B$ gives $M_{\text{sep}} \geq M_{\text{int}}$. □

Corollary 15 (Interleaving advantage)**.** *Let $B = n/2$ be the CKKS slot budget and let L denote the length of the two streams $(d_\bullet, n_\bullet)$. Separate packing uses $M_{\text{sep}} = 2\lceil L/B \rceil$ ciphertexts; interleaving uses $M_{\text{int}} = \lceil 2L/B \rceil$. Then $M_{\text{int}} \leq M_{\text{sep}}$ with $M_{\text{sep}} - M_{\text{int}} \in \{0, 1\}$ and*

$$\frac{M_{\text{sep}}}{M_{\text{int}}} \in [1,\, 2].$$

More precisely:

(i) $M_{\text{sep}}/M_{\text{int}} = 2$ *when* $L \le B/2$.

(ii) $M_{\text{sep}}/M_{\text{int}} = (2c+2)/(2c+1) \in (1, 4/3]$ *when* $L = cB + r$ *with* $c \ge 1$ *and* $0 < r \le B/2$.

(iii) $M_{\text{sep}}/M_{\text{int}} = 1$ *when* $L = cB + r$ *with* $c \ge 1$ *and* $B/2 < r < B$.

Proof. $M_{\text{int}} \le M_{\text{sep}}$ follows from Lemma 14.

Write $L = qB + r$ with $q \ge 0$ and $0 \le r < B$.

Formulas for M_{int} *and* M_{sep}. $\lceil L/B \rceil = q + \mathbf{1}_{r>0}$, so $M_{\text{sep}} = 2q + 2 \cdot \mathbf{1}_{r>0}$. For $M_{\text{int}} = \lceil 2L/B \rceil = 2q + \lceil 2r/B \rceil$:

$$\lceil 2r/B \rceil = \begin{cases} 0 & r = 0, \\ 1 & 0 < r \le B/2, \\ 2 & B/2 < r < B. \end{cases}$$

Therefore:

$$M_{\text{sep}} - M_{\text{int}} = \begin{cases} 0 & r = 0, \\ 1 & 0 < r \le B/2, \\ 0 & B/2 < r < B, \end{cases}$$

confirming $M_{\text{sep}} - M_{\text{int}} \in \{0, 1\}$.

Case (i): $L \le B/2$. Then $q = 0$, $r = L > 0$, so $M_{\text{int}} = 1$, $M_{\text{sep}} = 2$, and $M_{\text{sep}}/M_{\text{int}} = 2$.

Case (ii): $L = cB + r$, $c \ge 1$, $0 < r \le B/2$. $M_{\text{int}} = 2c + 1$, $M_{\text{sep}} = 2c + 2$, so $M_{\text{sep}}/M_{\text{int}} = (2c+2)/(2c+1)$. At $c = 1$ this is 4/3; it decreases toward 1 as $c \to \infty$.

Case (iii): $L = cB + r$, $c \ge 1$, $B/2 < r < B$. $M_{\text{int}} = 2c + 2 = M_{\text{sep}}$, so $M_{\text{sep}}/M_{\text{int}} = 1$.

Combining all cases: $M_{\text{sep}}/M_{\text{int}} \in [1, 2]$. □

S7 Communication Complexity

Theorem 16 (Communication: implemented plain federated KM)**.** *In the implemented protocol: R1 uplink:* $\sum_{k=1}^{K} |T_k^{\text{all}}|$ *scalars. R1 downlink:* $K\,|T_{\text{all}}|$ *scalars. R2 uplink:* $2K\,|T_{\text{all}}|$ *scalars. In an event-time-only variant where R2 counts are aligned over* T *instead of* T_{all}*, R2 uplink reduces to* $2K\,|T|$ *scalars.*

Proof. R1 uplink. Each site k uploads T_k^{all} ($|T_k^{\text{all}}|$ scalars). Summing: $\sum_k |T_k^{\text{all}}|$.

R1 downlink. The coordinator broadcasts T_{all} to each of K sites: $K|T_{\text{all}}|$ scalars.

R2 uplink (implementation). Each site uploads $(n_i^{(k)}, d_i^{(k)})$ for $t_i \in T_{\text{all}}$: $2|T_{\text{all}}|$ scalars per site, $2K|T_{\text{all}}|$ total.

R2 uplink (event-time-only variant). If counts are aligned over T instead of T_{all}, each site uploads $2|T|$ scalars, giving $2K|T|$ total. □

Theorem 17 (Communication: CKKS + threshold)**.** *If each client uploads* $M \in \{M_{\text{sep}}, M_{\text{int}}\}$ *ciphertexts in R2 (the value of* M *is identical across sites since all pack over the same global alignment grid* T_{all}*), the total R2 uplink is* KM *ciphertexts. Threshold decryption with a committee of size* R *requires* RM *partial shares, where* $M \in \{M_{\text{sep}}, M_{\text{int}}\}$ *is the number of aggregated blocks to be decrypted. Equivalently,*

$$M_{\text{int}} = \left\lceil \frac{2L}{B} \right\rceil, \qquad M_{\text{sep}} = 2\left\lceil \frac{L}{B} \right\rceil,$$

where L *is the packed grid length.*

Proof. R2 uplink. R1 remains in plaintext. In R2, each site encrypts its count vectors into M ciphertext blocks. All sites pack over the same grid with the same slot capacity B, so M is identical across sites. Total R2 uplink: KM ciphertexts.

For interleaved packing, $2L$ values fill $\lceil 2L/B \rceil$ ciphertexts (Lemma 14), giving $M = M_{\text{int}}$. For separate packing, two streams of length L require $2\lceil L/B \rceil$ ciphertexts, giving $M = M_{\text{sep}}$.

Decryption shares. Each of R decryptors produces one partial share per aggregated block, yielding RM shares total. □

Corollary 18 (Scaling laws)**.** *Implemented plain: $\Theta(K)$ for fixed $|T_{\text{all}}|$ and $\Theta(|T_{\text{all}}|)$ for fixed K; in an event-time-only variant, the same asymptotics hold with $|T|$ replacing $|T_{\text{all}}|$.*
CKKS: R2 uplink is linear in K and in $\lceil L/B \rceil$; share traffic is linear in R and in $\lceil L/B \rceil$, where L is the packed grid length. Interleaving satisfies $\lceil 2L/B \rceil \le 2\lceil L/B \rceil$.

Proof. Plain (implementation). By Theorem 16, R2 uplink is $2K|T_{\text{all}}|$: $\Theta(K)$ for fixed $|T_{\text{all}}|$ and $\Theta(|T_{\text{all}}|)$ for fixed K. In an event-time-only variant, R2 uplink is $2K|T|$, and the same asymptotics hold with $|T|$ replacing $|T_{\text{all}}|$.

CKKS. By Theorem 17, R2 uplink is KM ciphertexts with $M = \Theta(\lceil L/B \rceil)$ (both M_{int} and M_{sep} are within a factor of 2 of $\lceil L/B \rceil$). Hence R2 uplink is linear in K and in $\lceil L/B \rceil$. Share traffic RM is linear in both R and $\lceil L/B \rceil$. The inequality $\lceil 2L/B \rceil \le 2\lceil L/B \rceil$ was proved in Lemma 14. □

S8 Computational Complexity

Proposition 19 (Computational cost law)**.** *The per-round work satisfies*

$$W(mode) = \alpha K M_\star + \beta R M_\star + \gamma M_\star + \delta_1 L + \delta_2 |T|,$$

where $M_\star = M_{\text{int}}$ or M_{sep} depending on packing, L is the packed grid length, and $\alpha, \beta, \gamma, \delta_1, \delta_2 > 0$ are mode-independent constants. In the current implementation, $L = |T_{\text{all}}|$; in an event-time-only packing variant, $L = |T|$.

Proof. We identify five computational components of the encrypted pipeline.

Term 1 ($\alpha K M_\star$): server homomorphic addition. For each of $M_\star$ ciphertext blocks, the coordinator adds K ciphertexts ($K-1$ pairwise additions). Each addition has cost proportional to the ciphertext size (fixed by the CKKS parameters). Total: $\Theta(KM_\star)$.

Term 2 ($\beta R M_\star$): partial-share generation. Each of R decryptors produces one partial share per aggregated block. Per-share cost is a fixed function of the CKKS parameters. Total: $\Theta(RM_\star)$.

Term 3 ($\gamma M_\star$): fusion. For each of $M_\star$ blocks, the combiner fuses R partial shares. The per-block fusion cost depends on R and the CKKS parameters, both of which are fixed independently of the packing mode. Writing this per-block cost as γ: total is $\gamma M_\star$.

Term 4 ($\delta_1 L$): packed-vector decode. After fusion, the combiner decodes CKKS plaintext slots. The total number of slots is proportional to L: cost $\Theta(L)$.

Term 5 ($\delta_2|T|$): KM pass. The Kaplan–Meier product $\hat{S}(t_i) = \hat{S}(t_{i-1})(1 - d_i/n_i)$ iterates over $|T|$ event times: cost $\Theta(|T|)$.

Summing the five terms yields the stated cost model. The constants depend on the CKKS parameter set but not on the choice between interleaved and separate packing ("mode-independent"). □

Corollary 20 (Runtime impact of interleaving)**.** *With W_{sep} and W_{int} the total work under separate vs. interleaved packing,*

$$\frac{W_{\text{sep}}}{W_{\text{int}}} = \frac{(\alpha K + \beta R + \gamma) M_{\text{sep}} + \delta_1 L + \delta_2 |T|}{(\alpha K + \beta R + \gamma) M_{\text{int}} + \delta_1 L + \delta_2 |T|} \le \frac{M_{\text{sep}}}{M_{\text{int}}} \le 2,$$

where the last inequality follows from Corollary 15. In the three regimes:

(a) If $L \le B/2$*, then* $W_{\text{sep}}/W_{\text{int}} \le 2$ *(idealized* 50% *speedup upper bound).*

(b) If $(M_{\text{sep}}, M_{\text{int}}) = (4, 3)$*, then* $W_{\text{sep}}/W_{\text{int}} \le 4/3$ *(up to* 25%*).*

(c) If $M_{\text{sep}} = M_{\text{int}}$*, packing does not change runtime.*

Moreover, the fixed terms $\delta_1 L + \delta_2 |T|$ *strictly reduce the realized ratio below* $M_{\text{sep}}/M_{\text{int}}$ *whenever they are positive.*

Proof. By Proposition 19, define $A := \alpha K + \beta R + \gamma > 0$ and $F := \delta_1 L + \delta_2 |T| \ge 0$. Then

$$\frac{W_{\text{sep}}}{W_{\text{int}}} = \frac{A\, M_{\text{sep}} + F}{A\, M_{\text{int}} + F}.$$

Upper bound. Since $M_{\text{sep}} \ge M_{\text{int}} \ge 1$ (Corollary 15):

$$\frac{A\, M_{\text{sep}} + F}{A\, M_{\text{int}} + F} \le \frac{A\, M_{\text{sep}} + F \cdot (M_{\text{sep}}/M_{\text{int}})}{A\, M_{\text{int}} + F} = \frac{M_{\text{sep}}}{M_{\text{int}}},$$

where the inequality uses $F \le F \cdot (M_{\text{sep}}/M_{\text{int}})$ since $M_{\text{sep}}/M_{\text{int}} \ge 1$. By Corollary 15, $M_{\text{sep}}/M_{\text{int}} \le 2$.

Strict reduction when $F > 0$*.* If $F > 0$ and $M_{\text{sep}} > M_{\text{int}}$, then for $a > b > 0$ and $c > 0$: $(a + c)/(b + c) < a/b$ (adding a positive constant to both numerator and denominator pulls the ratio toward 1). Hence $W_{\text{sep}}/W_{\text{int}} < M_{\text{sep}}/M_{\text{int}}$.

The three cases follow by substituting the values of $M_{\text{sep}}/M_{\text{int}}$ from Corollary 15. □

S9 NCCTG Lung Cancer Dataset

Setup. We reuse the same protocol, CKKS parameters, packing modes (interleaved vs. separate), and evaluation criteria as in the main synthetic experiments. We sweep the number of providers $K \in \{2, 10, 30, 50, 70, 90, 100\}$ under non-IID label splits and report means (and CIs per K) for all metrics used in the paper.

Figure S1 summarizes eight diagnostics for NCCTG lung (non-IID). Overlays in Figure S2 show HE curves vs. pooled oracle. Figure S3 shows runtime vs. K.

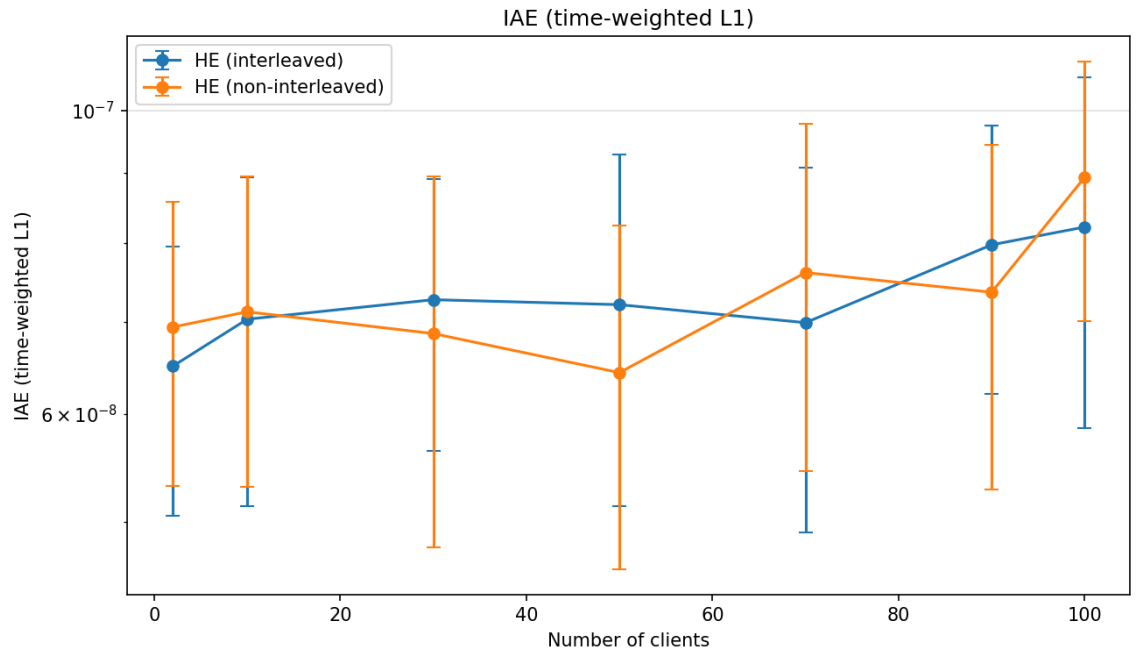

(a) IAE(S)

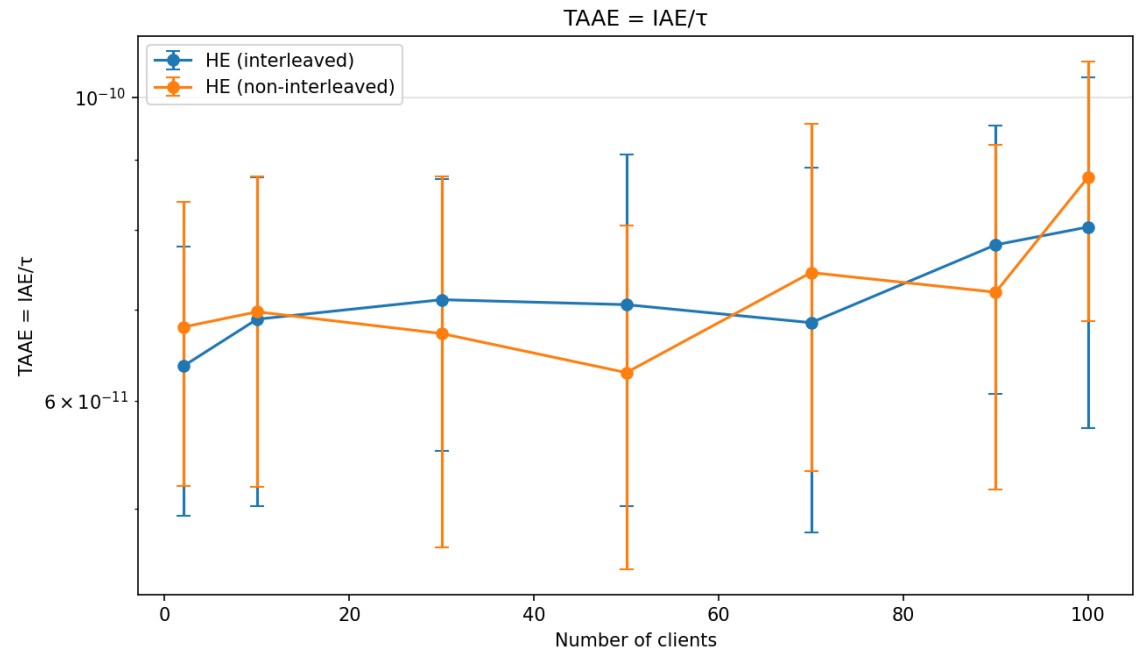

(b) TAAE(S)

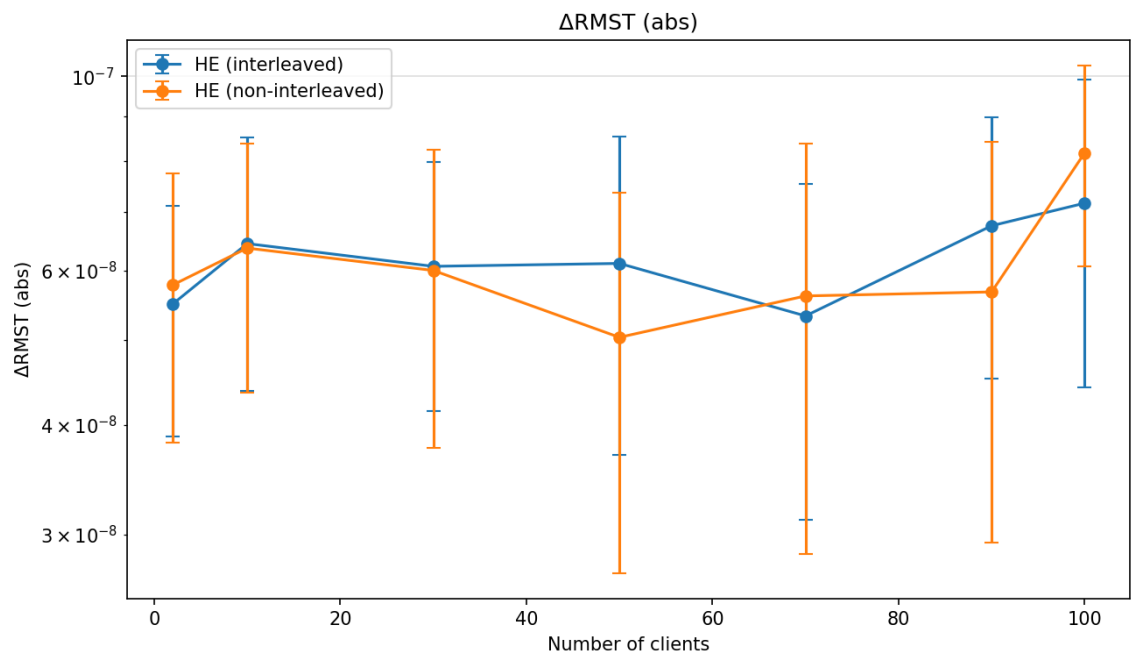

(c) $|\Delta\text{RMST}|$

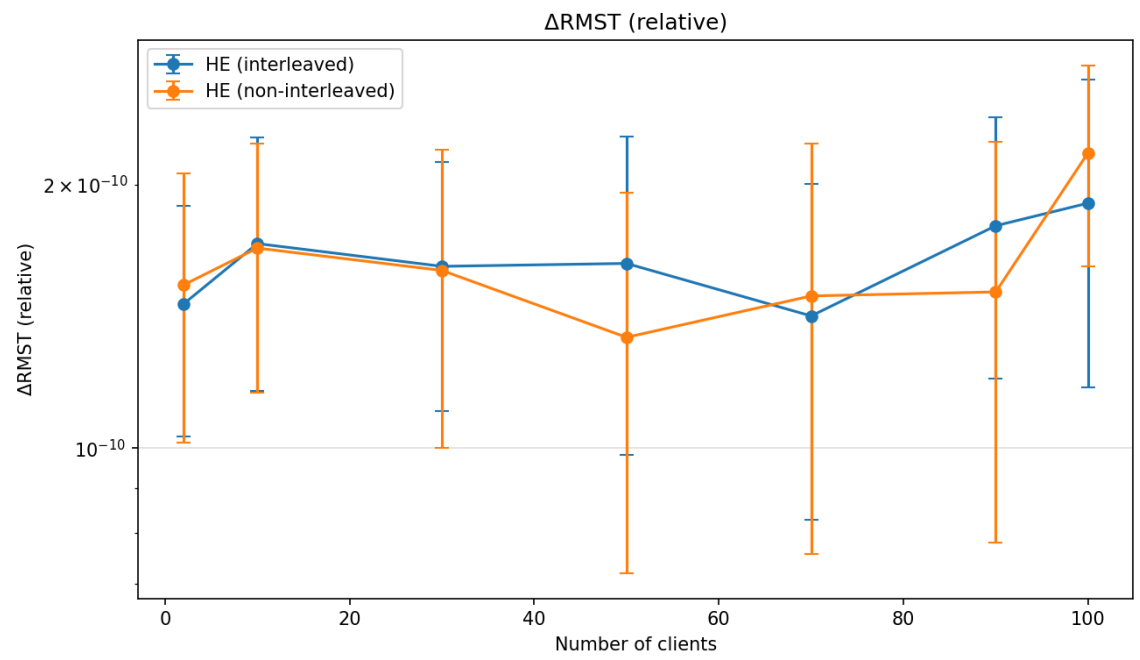

(d) $\Delta\text{RMST}_{\text{rel}}$

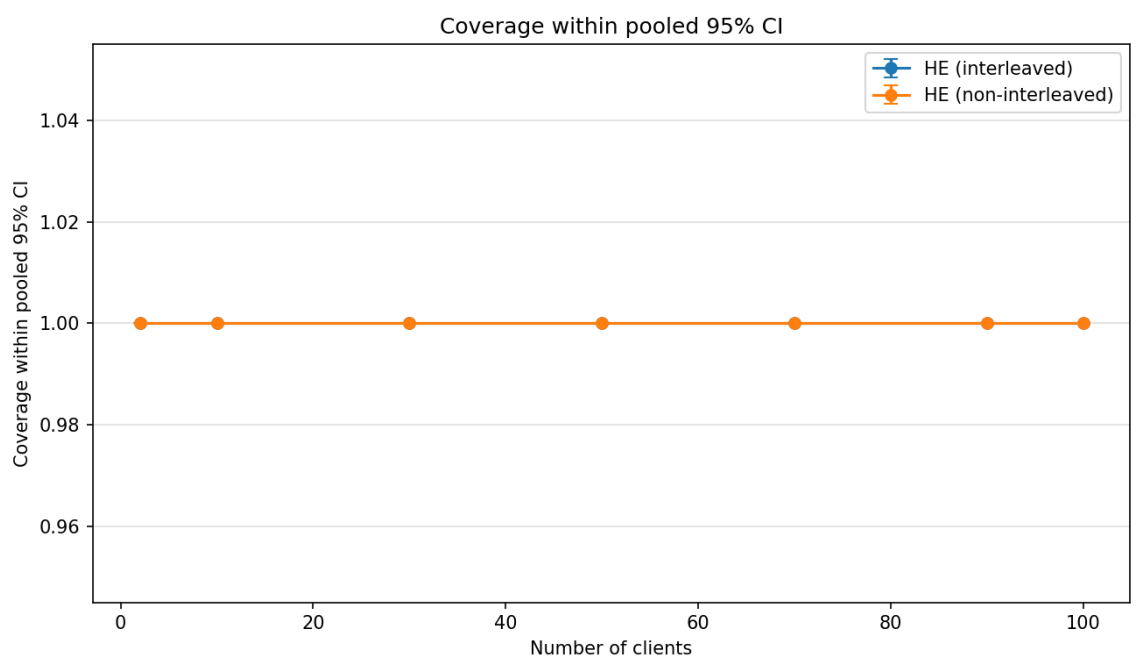

(e) 95% coverage

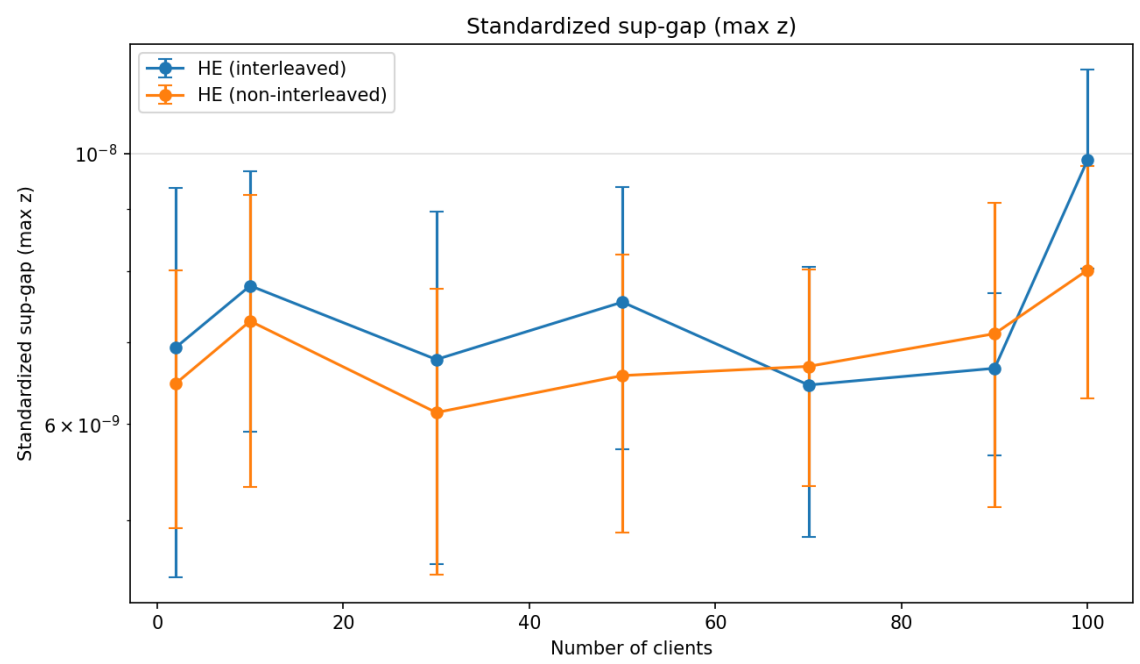

(f) Standardized sup-gap supZ

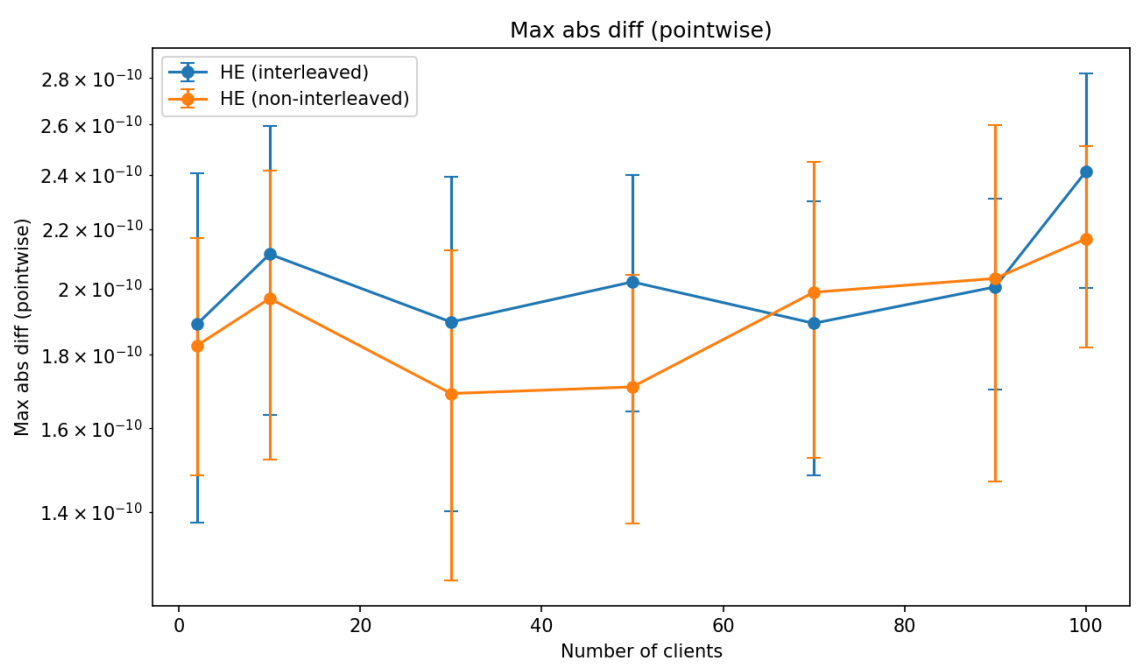

(g) $\max_t |H_{\text{HE}} - H_{\text{oracle}}|$

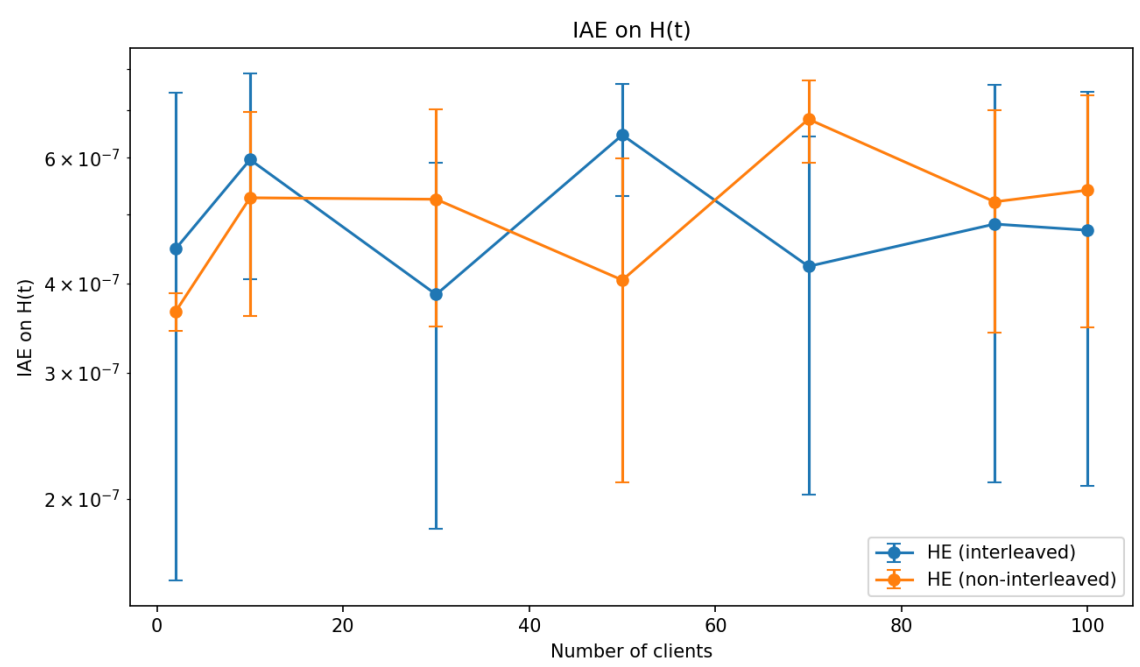

(h) IAE_H

Figure S1: NCCTG lung cancer (non-IID): numerical fidelity of HE–federated KM vs. pooled oracle across K.

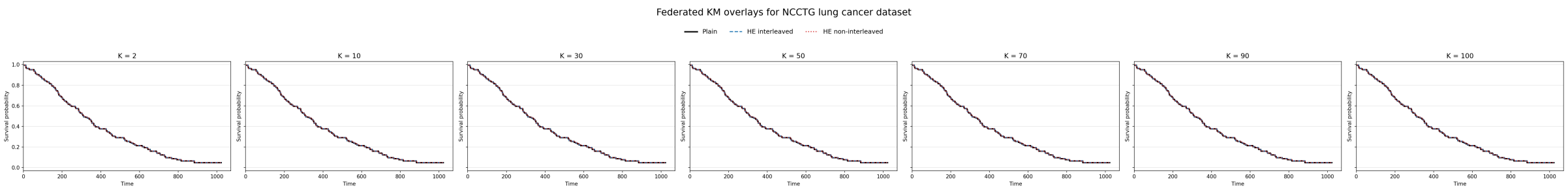

Figure S2: NCCTG lung cancer (non-IID): KM overlays (Plain, HE–interleaved, HE–separate).

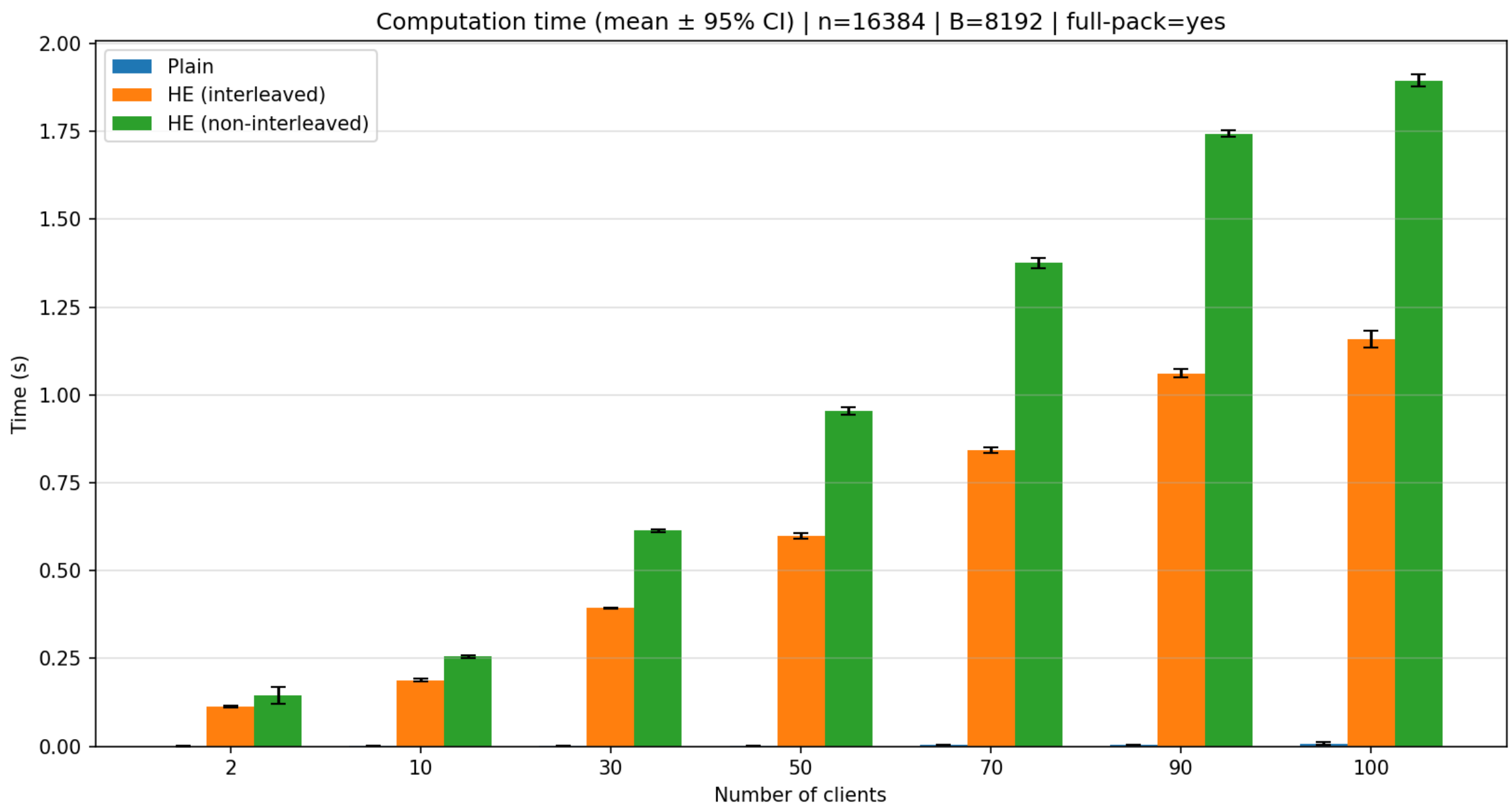

Figure S3: NCCTG lung cancer (non-IID): runtime vs. number of providers K.
